# A candidate multi-epitope vaccine against SARS-CoV-2

**DOI:** 10.1038/s41598-020-67749-1

**Published:** 2020-07-02

**Authors:** Tamalika Kar, Utkarsh Narsaria, Srijita Basak, Debashrito Deb, Filippo Castiglione, David M. Mueller, Anurag P. Srivastava

**Affiliations:** 1 Department of Life Sciences, Garden City University, Bangalore, Karnataka India; 2 Institute for Applied Computing, National Research Council of Italy, Via dei Taurini, Rome, Italy; 3 Center for Genetic Diseases, The Chicago Medical School, Rosalind Franklin University of Medicine and Science, North Chicago, USA

**Keywords:** Biochemistry, Computational biology and bioinformatics, Immunology

## Abstract

In the past two decades, 7 coronaviruses have infected the human population, with two major outbreaks caused by SARS-CoV and MERS-CoV in the year 2002 and 2012, respectively. Currently, the entire world is facing a pandemic of another coronavirus, SARS-CoV-2, with a high fatality rate. The spike glycoprotein of SARS-CoV-2 mediates entry of virus into the host cell and is one of the most important antigenic determinants, making it a potential candidate for a vaccine. In this study, we have computationally designed a multi-epitope vaccine using spike glycoprotein of SARS-CoV-2. The overall quality of the candidate vaccine was validated in silico and Molecular Dynamics Simulation confirmed the stability of the designed vaccine. Docking studies revealed stable interactions of the vaccine with Toll-Like Receptors and MHC Receptors. The in silico cloning and codon optimization supported the proficient expression of the designed vaccine in *E. coli* expression system. The efficiency of the candidate vaccine to trigger an effective immune response was assessed by an in silico immune simulation. The computational analyses suggest that the designed multi-epitope vaccine is structurally stable which can induce specific immune responses and thus, can be a potential vaccine candidate against SARS-CoV-2.

## Introduction

Wuhan, a city in China, witnessed the outbreak of a febrile respiratory illness on 19th December 2019 due to the coronavirus provisionally named as 2019-nCoV and later SARS-CoV-2^1,2^. The disease caused by this coronavirus was named as COVID-19^1,2^. Since then, the world is experiencing a grave situation of global public health emergency due to the viral pandemic of severe febrile pneumonia like respiratory syndrome caused by the novel coronavirus^2^. Coronaviruses are known to have caused three epidemics in the last two decades, namely COVID-19 in 2019/20, Severe Acute Respiratory Syndrome (SARS) in 2002, and Middle East Respiratory Syndrome (MERS) in 2012^3^. As of June 3rd 2020, total cases of SARS-CoV-2 confirmed globally by World Health Organization are 6,287,771 with 379,941 reported deaths (https://www.who.int/emergencies/diseases/novel-coronavirus-2019/situation-reports).

Human coronavirus (H-CoV) is a member of *Coronaviridae* family, a virus family characterized with the largest RNA genome (26–32 kb), among all of the viruses known till date^4–6^. A lipid envelope bilayer containing the spike and membrane proteins surround the positive stranded RNA genome of this virus^7^. The spike protein binds to the host cell receptors and releases the viral genome into the host cell, thereby facilitating the viral replication^8^. Coronaviruses (CoVs) are mostly associated with respiratory illness and common cold^9^, but can also cause infections in Central Nervous System (CNS)^10^. To date, four genera of coronaviruses (α, β, γ, δ) have been identified^11^. Human coronaviruses (H-CoVs) belong to α (HCoV-229E and NL63) and β (MERS-CoV, SARS-CoV, HCoV-OC43, HCoV-HKU1 and SARS-CoV-2) genera of coronavirus, respectively^11^.

In late December 2019, patients with Acute Respiratory Distress Syndrome (ARDS) along with cough, fever and dyspnoea due to an unknown microbial infection were recorded in Wuhan, China^12^. Viral genome sequencing of five pneumonia patients, hospitalized between 18th December and 29th December 2019, reported the presence of a previously unknown β-CoV strain in all of the 5 hospitalized patients^12^. There was around 88% sequence similarity between the novel β-CoV strain and two bat-derived severe acute respiratory syndromes (SARS)-like coronaviruses namely, bat-SL-CoVZC45 and bat-SL-CoVZXC21, while MERS-CoV displayed a sequence identity of about 50% with the novel β-CoV^12^.

Coronavirus infection in humans is primarily guided by interactions between envelope anchored spike glycoprotein (S-protein) of CoV and angiotensin converting enzyme 2 (ACE2) of the host cell receptor^13,14^. The viral RNA genome is released into the cytoplasm once the virus enters the cells and is then translated into two polyproteins and structural proteins, after which the viral genome starts to replicate^11^. The S protein is composed of two subunits, one subunit, S1, is the Receptor Binding Domain (RBD) and the other subunit, S2, is responsible for the fusion of viral membrane and the host cellular membrane^15^. An overall 75% sequence similarity was seen between SARS-CoV-2 and previously identified SARS-CoV spike protein^16,17^. In addition, it is also reported, that the coronavirus S protein is a major determinant of virus entry into host cells^3^. Hence, the spike like glycoprotein is a potent choice for vaccine designing.

**Figure 1:**
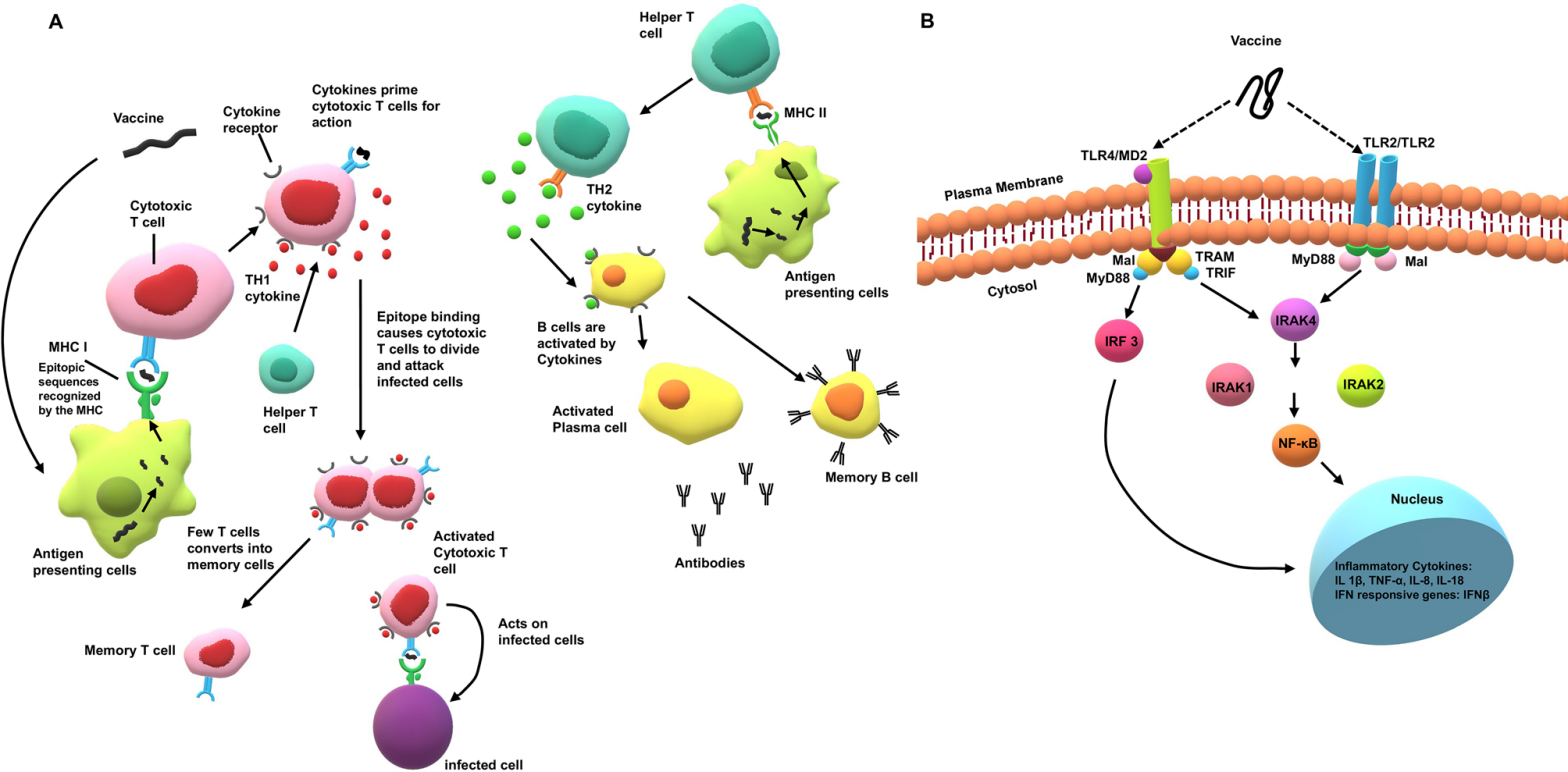
(**A**) The designed multi-epitope vaccine has the capacity to trigger both humoral and cell mediated immunity. The vaccine is processed in the antigen presenting cells (APCs) and the antigenic epitopes are recognized by MHC I receptors which further stimulates cytotoxic T cell (T_c_ cell) development. T_c_ cells trigger cytokine production which causes cytotoxic T cells to divide and attack the infected cells. The activated T cells also differentiate into memory T cells. Similarly, vaccine antigen is processed and presented in context of MHC class II molecule. B cells differentiate into plasma cells and memory B cells upon activation by cytokines. Further, the activated B cell or plasma cell produces the neutralizing antibodies responsible for clearing an infection. (**B**) TLR signal transduction pathway: TLR 2 homodimer utilizes MyD88 and MAL as primary adapters to activate NF-κB that triggers inflammatory cytokine secretion. TLR4 uses four primary adapters namely MyD88, MAL, TRIF and TRAM for NF-κB secretion which in turn induce inflammatory cytokine secretion activating IFN pathway.

The vaccine candidate once introduced into the body is detected by the host innate immune system by using pattern recognition receptors (PRRs) to identify the pathogen‐associated molecular patterns (PAMPs). The pathogen-associated patterns contained in vaccine antigens attract dendritic cells, monocytes, and neutrophils that patrol throughout the body^18^. Through the pattern-recognition receptors (among which the Toll-like receptors play an important role) the host cells sense the potential danger when they detect a pathogen and become activated^18^. Elicitation of sufficient “danger signals” by the vaccine antigens or adjuvants activate monocytes and dendritic cells. They modulate their surface molecule's expression, and develop pro inflammatory cytokines and chemokines resulting in the extravasation and attraction of monocytes, granulocytes, and natural killer cells. This leads to the generation of an inflammatory microenvironment where the monocytes differentiate into macrophages and immature dendritic cells are activated^19^. This activation alters the expression of the homing receptors at the cell surface and triggers the migration of dendritic cells towards the lymph nodes where the activation of T and B lymphocyte takes place. On contact with naïve T cells, the T cells differentiate into regulatory CD4^+^ cells that maintain immune tolerance^20^. The immature dendritic cells recognize the protein vaccine antigen and then migrate towards the lymph node. During this migration, the dendritic cells mature and their surface expression of molecules changes^21^. Simultaneously, processing of antigens into smaller fragments occur which is then displayed at the cell surface in the grooves of MHC (human leukocyte antigen [HLA] in humans) molecules. The peptides from the antigens that are produced in the cytosol of infected cells are presented by MHC class I molecules and phagocytised antigens are essentially displayed on MHC class II molecules^22–25^. The antigenic peptides displayed by class II MHC molecules are recognized by CD4^+^ T cells whereas, CD8^+^ T cells bind to class I MHC-peptide complexes^26^. Activated CD4^+^ T cells secrete cytokines and are responsible for the further activation of B cells required for proper antibody generation^27^ (Fig. 1).

**Figure 2:**
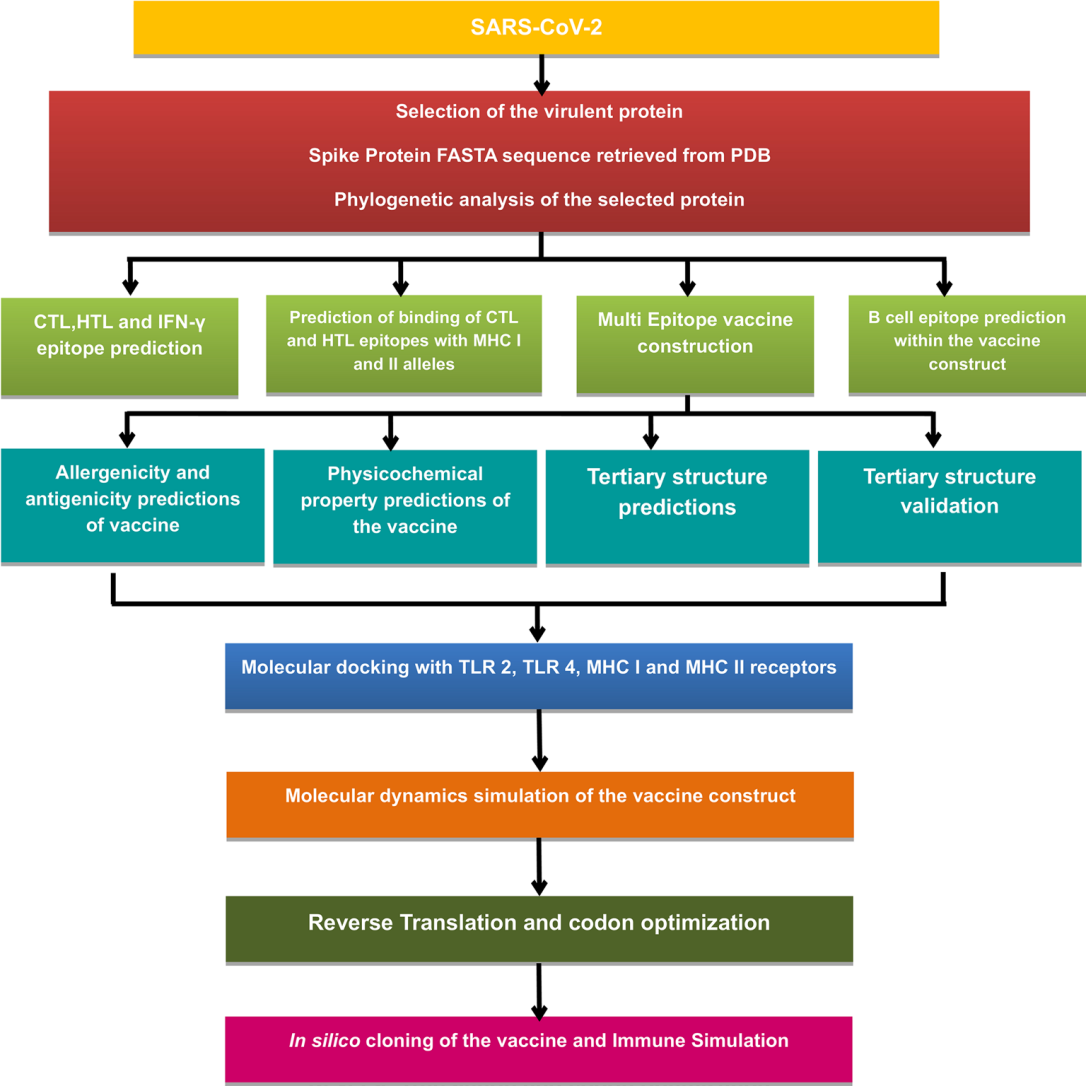
Flowchart for the designed study. The entire approach used in the study comprises of several phases, which involves identifying the target protein and its phylogenetic analysis. Epitope predictions from the chosen protein (CTL, HTL, IFN-γ and B cell epitopes); vaccine construction and its quality check. Molecular Docking with immune cell receptor, followed by MDS to check vaccine’s stability. Lastly, codon adaptation and immune simulation to understand how the vaccine elicits an immune response.

The conventional method of vaccine designing, involving entire organisms or large proteins lead to unnecessary antigenic load along with increased chances of allergenic responses^28^. This problem can be overcome by peptide based vaccines comprising short immunogenic peptide fragments with the ability to elicit strong and targeted immune responses, avoiding the chances of allergenic reactions^28^. Recent advancements in computational biology have opened up new doors for designing effective vaccines in silico^29–31^. In this study, the in silico approach has been applied for attaining a multi-epitope vaccine against SARS-CoV-2 that comprises of spike glycoprotein epitopes which induces the activation of cytotoxic T lymphocytes (CTLs), helper T lymphocytes (HTLs) and interferon-γ (IFN-γ) (Fig. 2).

## Results

### Sequence retrieval and phylogenetic analysis

**Figure 3:**
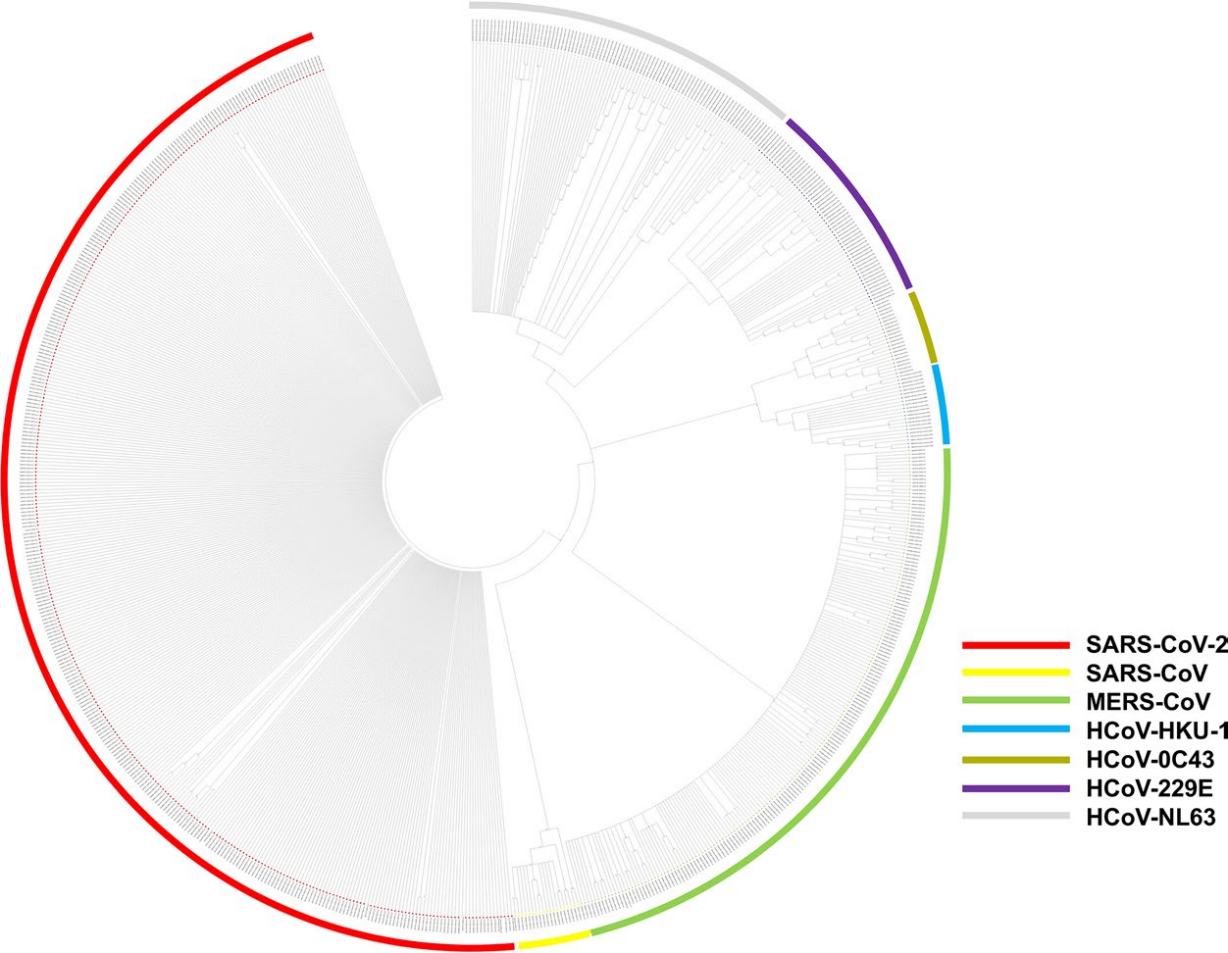
Phylogenetic analysis of spike glycoprotein of 7 coronaviruses (HCoV-NL63, HCoV-229E, HCoV-OC43, HKU-1, MERS-CoV, SARS-CoV and SARS-CoV-2) infecting humans. SARS-CoV-2 has shown very low rate of diversification.

The spike glycoprotein sequence of SARS-CoV-2 was retrieved from PDB (6VSB). Phylogenetic analysis of the SARS-CoV-2 glycoprotein was performed in order to check the evolutionary relationship of SARS-CoV-2 with other coronaviruses (HCoV-NL63, HCoV-229E, HCoV-0C43, HKU-1, MERS-CoV, SARS-CoV) (Fig. 3). The analysis revealed that the glycoprotein variants of SARS-CoV-2 clustered together in a single clade, having the most common ancestry with SARS-CoV and MERS-CoV (Fig. 3). The variants of SARS-CoV-2 that clustered together had very less branching, indicating minimum variations. Hence, the vaccine designed against one strain can be used for all the other strains of SARS-CoV-2. Similarly, the phylogenetic analysis of different SARS-CoV-2 strains isolated from different countries was conducted to determine if a single vaccine can be used against all the different strains of the virus isolated from various parts of the world (Supplementary Fig. S1). The results indicated that all the glycoproteins of different strains of SARS-CoV-2, isolated from different countries were closely related to one another, suggesting that a vaccine designed against one strain would be effective against all the other strains of viruses isolated from different countries (Supplementary Fig. S1).

### T cell epitope prediction

**Figure 4:**
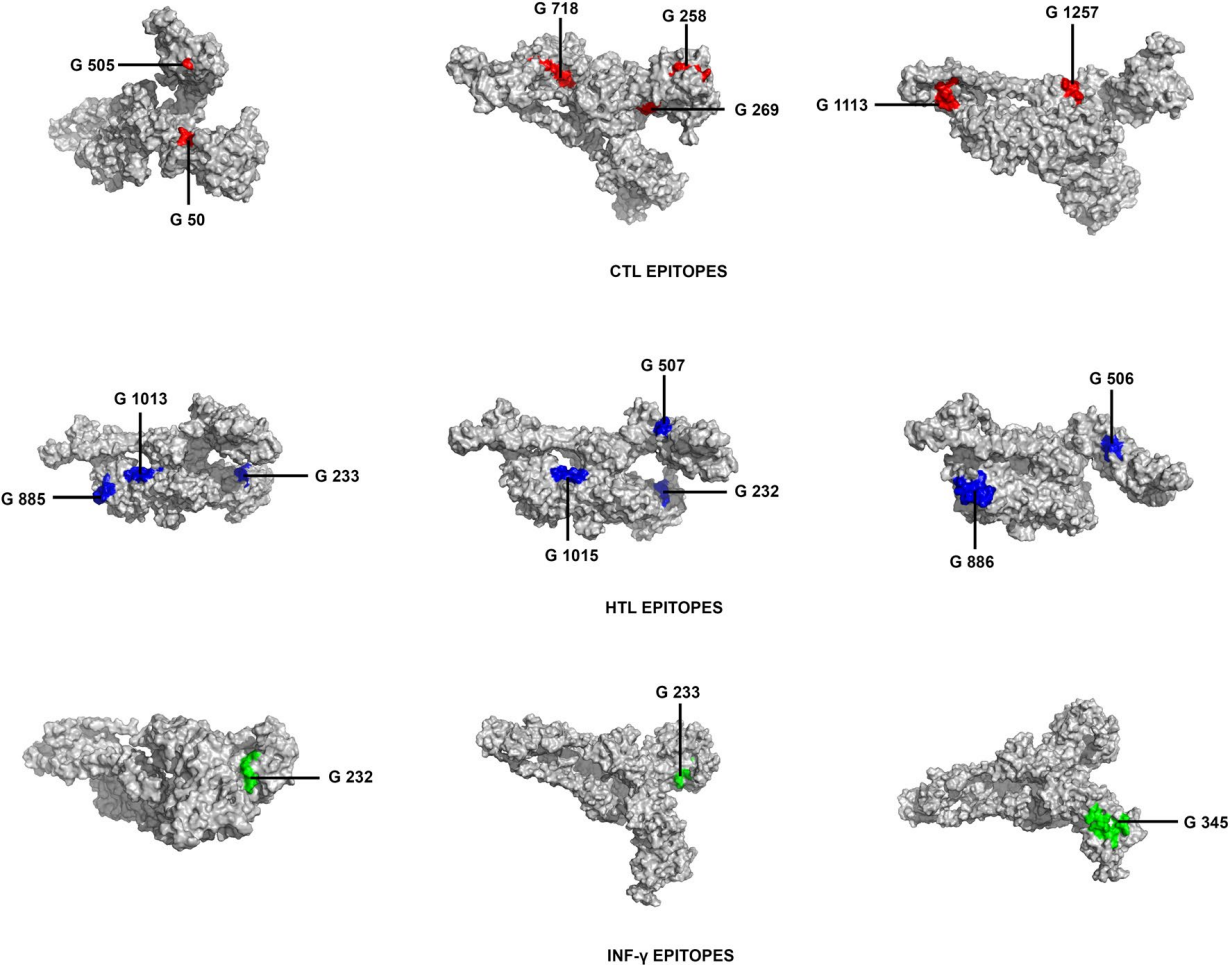
Tertiary structure of the spike protein with CTL epitopes marked by red colour, HTL epitopes are marked by blue colour and IFN-γ epitopes marked by green colour, showing their surface positions.

An ideal prophylactic vaccine should mimic the natural immunity induced by an infection with the generation of a long-lasting adaptive immunity, where both CTL and HTL epitopes play an important role^32^. The CTL epitopes are responsible for developing long lasting cellular immunity which has the ability to eliminate the circulating virus and the virus infected cells^33^. On the other hand, HTL epitopes play a crucial role in generating both humoral and cellular immune responses. These epitopes elicit a CD4^+^ helper response, which is not only necessary for the development of protective CD8^+^ T-cell memory but also activation of B-cells for antibody generation^34,35^. Therefore, an effective vaccine candidate should consist of the important CTL and HTL receptor specific epitopes. In this study, CTL epitopes were predicted using NetCTL1.2 and IEDB consensus methods whereas, HTL epitopes were predicted using NetMHC II pan 3.2 server as shown in Tables 1 and 2 (Supplementary Table S1, S2). In order to identify the best epitopes, the predicted epitopes were subjected to various immune filters and those having high binding affinity to MHC class I and class II alleles were selected. The criteria for screening out the epitopes were: they should be promiscuous, should be antigenic and should be immunogenic. The antigenicity of the epitopes was predicted using VaxiJen v2.0 and immunogenicity was predicted using IEDB class I immunogenicity server. The 3D structure of spike glycoprotein was modelled using I-TASSER and the epitopes considered for vaccine construction were visualized on the same (Fig. 4).

**Table 1:** CTL epitopes predicted using NetCTL 1.2 showing promiscuity.

Epitopes	Supertype	MHC class I allele	Binding score	IC50	Position	Prediction score	Immunogenicity score	Antigenicity score
QIITTDNTF	A24,A26,B58,B62	HLA-B*15:01	1.3	66.32	1,113	0.7939	0.15816	0.4253
		HLA-A*32:01	1.7	472.54				
YQPYRVVVL	A2,A24,B8,B39,B62	HLA-B*15:01	1.2	131.99	505	0.8143	0.1409	0.5964
		HLA-A*02:06	1.615	99.74				
FTISVTTEI	A2,A26,B58	HLA-A*68:02	0.2	3.05	718	1.1808	0.04473	0.8535
		HLA-B*58:01	0.4	48.78				
		HLA-A*02:06	0.6	8.29				
		HLA-A*26:01	0.615	481.17				
		HLA-A*02:01	0.8	25.37				
		HLA-A*02:03	0.94	9.07				
YLQPRTFLL	A2,B8,B39	HLA-A*02:01	0.3	5.36	269	1.5152	0.1305	0.4532
		HLA-A*02:06	0.96	16.55				
		HLA-B*08:01	1.0	147.76				
		HLA-A*02:03	1.005	15.24				
		HLA-A*24:02	1.115	406.74				
		HLA-A*23:01	1.275	278.62				
HSAWSHPQF	A1A24,B39,B58,B62	HLA-B*58:01	0.5	17.5	1,257	0.8279	0.0279	0.8569
		HLA-B*35:01	1.5	287.84				
STQDLFLPF	A1,A26,A24,B62	HLA-A*32:01	0.2	17.27	50	1.0468	0.06828	0.6619
		HLA-B*15:01	0.3	13.32				
		HLA-A*26:01	0.46	437.88				
		HLA-A*23:01	1.415	394.77				
WTAGAAAYY	A1,A26,B58,B62	HLA-A*26:01	0.11	11.63	258	3.1128	0.15259	0.6306
		HLA-A*30:02	0.115	16.16				
		HLA-A*01:01	0.17	12.27				
		HLA-A*68:01	1.185	30.13				
		HLA-B*35:01	1.2	66.67				
		HLA-B*15:01	1.6	132.2				

Epitopes with IC50 value < 500 nm were considered good binders towards specific alleles. VaxiJen v2.0 was used for predicting antigenicity scores keeping a threshold of 0.4.

**Table 2:** HTL epitopes showing promiscuity, as predicted using NetMHC II pan 3.2 server.

Epitopes	Position	Allele	Score	Antigenicity score
INITRFQTLLALHRS	233	DRB1*01:01	1.00	0.418
		DRB1*04:01	0.80	
		DRB1*04:05	0.25	
		DRB1*08:02	1.60	
		DRB1*11:01	0.60	
		DRB1*12:01	0.90	
		DRB1*15:01	0.30	
		DRB4*01:01	0.50	
		DPA1*02:01-DPB1*05:01	0.40	
		DPA1*02:01-DPB1*14:01	0.70	
		DRB5*01:01	0.12	
GINITRFQTLLALHR	232	DRB1*01:01	1.60	0.5582
		DRB4*01:01	0.50	
		DRB5*01:01	0.30	
		DPA1*03:01-DPB1*04:02	2.00	
		DPA1*02:01-DPB1*05:01	0.50	
		DPA1*02:01-DPB1*14:01	1.00	
		DPA1*02:01-DPB1*01:01	1.60	
		DRB1*04:01	1.00	
		DRB1*04:05	0.25	
		DRB1*11:01	1.30	
		DRB1*12:01	0.80	
		DRB1*15:01	0.25	
GWTFGAGAALQIPFA	885	DRB1*01:01	2.00	0.4665
		DRB1*09:01	0.20	
		DQA1*03:01-DQB1*03:02	0.60	
		DQA1*04:01-DQB1*04:02	0.40	
		DQA1*01:02-DQB1*06:02	0.60	
		DQA1*05:01-DQB1*03:01	0.10	
IRAAEIRASANLAAT	1,013	DRB1*04:01	1.40	0.6785
		DRB1*08:02	1.20	
		DRB1*13:02	1.90	
		DPA1*02:01-DPB1*14:01	0.80	
		DQA1*01:02-DQB1*06:02	0.30	
		DQA1*05:01-DQB1*03:01	1.00	
AAEIRASANLAATKM	1,015	DRB1*04:01	0.70	0.7125
		DRB1*08:02	0.70	
		DRB1*13:01	1.10	
		DPA1*02:01-DPB1*14:01	0.50	
		DQA1*01:02-DQB1*06:02	1.30	
		DRB3*02:02	1.10	
WTFGAGAALQIPFAM	886	DRB1*09:01	0.40	0.6670
		DQA1*03:01-DQB1*03:02	0.80	
		DQA1*04:01-DQB1*04:02	0.50	
		DQA1*01:02-DQB1*06:02	0.50	
		DQA1*05:01-DQB1*03:01	0.17	
QPYRVVVLSFELLHA	506	DPA1*02:01-DPB1*01:01	0.70	0.9109
		DPA1*01:03-DPB1*04:01	1.10	
		DPA1*03:01-DPB1*04:02	0.50	
		DPA1*02:01-DPB1*05:01	0.80	
		DPA1*01:03-DPB1*02:01	1.10	
PYRVVVLSFELLHAP	507	DPA1*02:01-DPB1*01:01	0.80	0.8161
		DPA1*01:03-DPB1*02:01	1.30	
		DPA1*03:01-DPB1*04:02	0.60	
		DPA1*02:01-DPB1*05:01	0.80	
		DPA1*01:03-DPB1*04:01	1.30	

VaxiJen v2.0 was used for predicting antigenicity scores keeping a threshold of 0.4.

### Multi-epitope vaccine construct, structural modeling, refinement and validation

**Figure 5:**
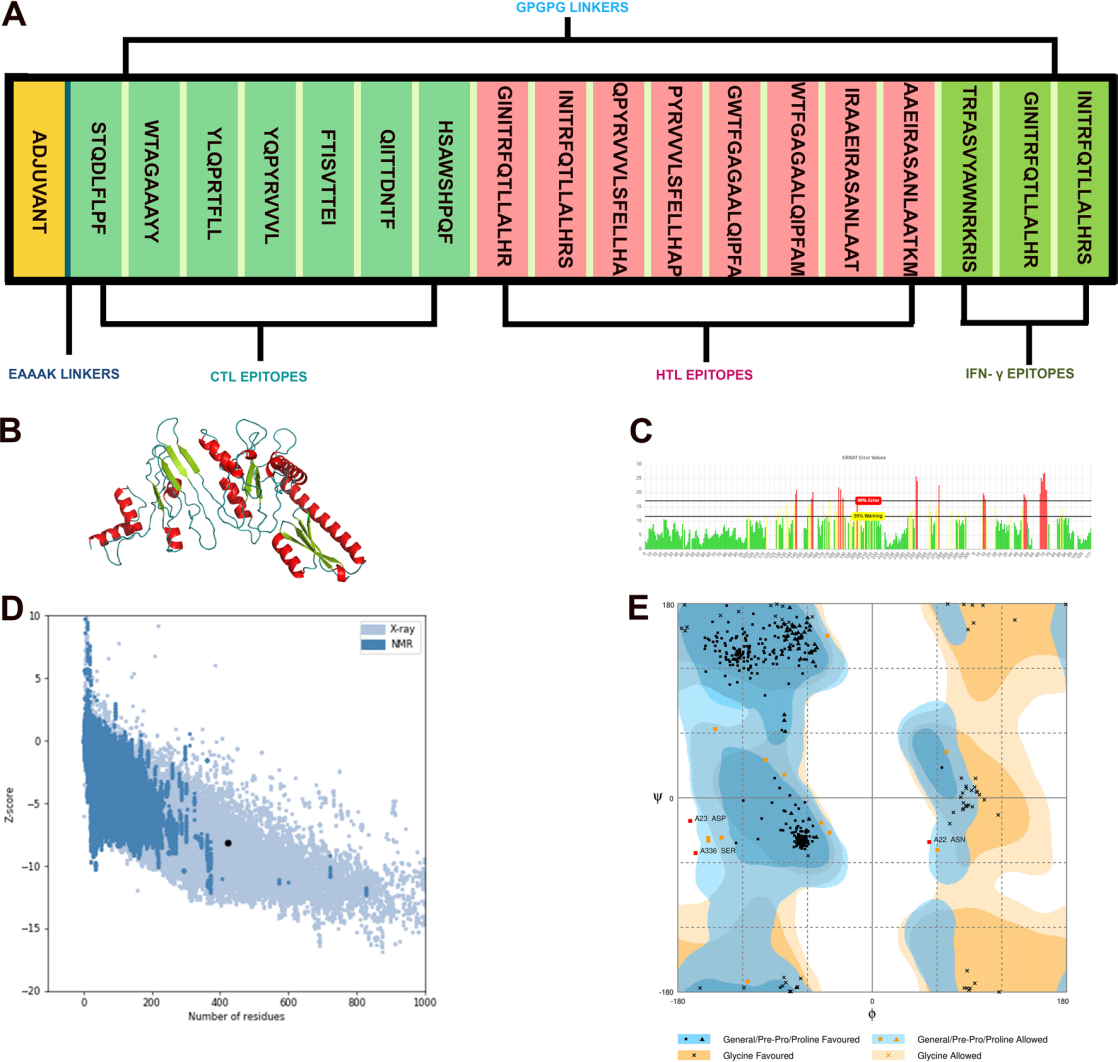
(**A**) Linear vaccine construct with CTL, HTL and IFN-γ depicted in sea green, pink and green boxes, respectively. EAAAK linker (deep blue) was used for linking the adjuvant and GPGPG linkers (pale green) were used for linking the epitopes. (**B**) 3D model of the final vaccine construct. Red, Limon and Blue represent the helical, sheet and loop region, respectively. (**C**) Validation of the vaccine structure by ERRAT with a score of 74.2947. (**D**) Validation of the structure with a Z-score of − 8.1 using ProSA. (**E**) Ramachandran plot analysing using RAMPAGE 96.4%, 2.9% and 0.7% in the favoured, allowed and outlier region, respectively.

The main criteria used for designing the linear vaccine construct were: 1. It should contain overlapping HTL and CTL epitopes (Supplementary Table S3), 2. It must be immunogenic, antigenic, but not an allergen, 3. It should have high affinity to HLA alleles and should be promiscuous. On basis of these parameters, a linear vaccine was constructed including 7 CTL, 8 HTL and 3 IFN-γ (Tables 1, 2, Supplementary Table S4) epitopes joined by GPGPG linkers which prevent the formation of junctional epitopes and also facilitate the immune processing of antigen^68^. Cholera Toxin B (CTB) adjuvant was attached to the N-terminal of the construct via EAAAK linker (Fig. 5A) in order to boost a long lasting immune response. The final vaccine construct consisted of 422 amino acids with a molecular weight of 44.15 kDa. The 3D models of the vaccine were generated using trRosetta server. In order to validate the structural quality of the predicted model, Ramachandran plot, Z-score and ERRAT analyses were performed. Amongst the predicted models, the best model was chosen (Fig. 5B) that had a Z-score of − 8.1, which was within the range of scores of comparable size proteins, indicating the reliability of the predicted model^36^ (Fig. 5D). The modelled structure was evaluated using RAMPAGE and was used for the generation of Ramachandran plot. The Ramachandran plot analysis of the 3D-model of the vaccine showed that 96.4% residues lied in favoured region, 2.9% residues in allowed and 0.7% residues in outlier regions, respectively which verifies the overall quality of the vaccine construct (Fig. 5E). Ideally for a model to be reliable, at least 90% of its residues should lie in the favoured region^37^. The total number of residues present in the favoured region for our 3D model was within the range of the ideal value (more than 90%), which confirms its reliability. The ERRAT score revealed after ERRAT analysis was 74.2947, representing the percentage of the protein falling below the rejection limit of 95%^38^ (Fig. 5C). Generally, an ERRAT score greater than 50 represents a good quality model^39^ and so, the score 74.2947 further validates our modelled structure.

### Immunogenic, allergenic and physicochemical evaluation of the vaccine construct

Immunogenicity is the ability to induce humoral and cellular immune responses while antigenicity is the ability to recognize a specific antigen accompanied by an immune response. Therefore, the vaccine candidate should be antigenic as well as immunogenic in nature^40^. The multi-epitope vaccine construct was found to be immunogenic as predicted by IEDB class I immunogenicity tool with a score of 6.65414 and as per the instruction of IEDB, higher score indicates a greater probability of eliciting an immune response. VaxiJen v2.0 confirmed the antigenicity of the vaccine with a score of 0.5107 (a score > 0.4 is considered to be antigenic). Allergenicity was checked in order to ensure that the candidate vaccine does not stimulate any allergic reactions once introduced into the body. The vaccine candidate was found to be non-allergen as predicted by AllerTOP and AllergenFP web servers. Evaluation of various physicochemical properties is essential for determining the safety and efficacy of the candidate vaccine^41^. Hence, various chemical and physical parameters associated with the vaccine were predicted in this study using ExPASy (Supplementary Material 1). The theoretical pI of the vaccine was found to be 9.96. The aliphatic index of the vaccine is 78.74 which suggest the vaccine to be of thermostable nature, higher the aliphatic index of a protein, greater is its thermostability^89^. The estimated half-life of the vaccine as predicted by ExPASy is 30 h in mammalian reticulocytes, > 20 h in yeast and > 10 h in *Escherichia coli*. The Grand average hydropathicity (GRAVY) score is − 0.088 (lower the GRAVY score, better is the solubility), which indicates the candidate vaccine is of hydrophilic nature, meaning, it can perform interaction with aqueous environment. The instability index of vaccine candidate was found to be 31.04, indicating stable nature of the protein. Generally a protein whose instability index is < 40 is classified as a stable protein^89^. Since the designed vaccine does not contain any transmembrane helices, no expression difficulties are to be anticipated in the production of vaccine (Supplementary Fig. S3). Also, the absence of signal peptides in the vaccine construct signifies prevention of protein localization (Supplementary Fig. S2).

### B cell epitope prediction

B cell epitopes have the ability to elicit humoral immunity as they are recognized by the B-cell receptors or secreted antibodies^42^. The presence of these epitopes in the designed vaccine play an important role in triggering efficient immune response. Therefore, in this study, the linear/continuous and conformational/discontinuous B cell epitopes were predicted by the ElliPro server using default parameters (Tables 3, 4). The visualisation of B cell epitopes in the final vaccine construct was done using PyMOL (The PyMOL Molecular Graphics System, Version 2.0 Schrödinger, LLC.) (Supplementary Fig. S4).

**Table 3:** Conformational/ discontinuous B cell epitopes in the multi-epitope vaccine, predicted by ElliPro server.

Discontinuous epitopes	Score
R(334), KMGPGPGTRFAS(361–372), YAWNRK(374–379), ISGPGPGGINITRFQTLLAL(381–400), RGPGPGINI(402–410), RFQTLLAL(412–419), RS(421–422)	0.766
M(1), DLCAEYHNTQIH(8–19), FSYTESLAGKREMAII(26–41), F(43), NGATFQVEVPGSQHIDSQKKAIERMKDTLRIA(45–76), LT(78–79), AKVEKLCV(81–88), NNK(90–92), PHAIAA(94–99), SM(101–102)	0.752
HAGPGPGPY(261–269), AGPGPGW(302–308)	0.647
L(114), YYGPGPGYL(131–139), GPGPGF(161–166), DNTFGPGPGHS(185–195), S(198)	0.608
FAMGPGPGIRA(320–330)	0.601
LPFGPGPGWT(116–125), W(197), FGPGPG(202–207)	0.579
ATGPGPGAAE(341–350)	0.522

**Table 4:** Linear/continuous B cell epitopes in the Vaccine construct, predicted by ElliPro server.

Linear epitopes	Position	Score
FSYTESLAGKREMAII	26	0.824
AWNRKRISGPGPGGINITRFQTLLALHRGPGPGINITRFQTLLALHRS	375	0.81
GATFQVEVPGSQHIDSQKKAIERMKDTLRIAYLTEAKVEKLCVWNNKTPHAIAAISM	46	0.745
HAGPGPGPY	261	0.731
KMGPGPGTRFA	361	0.721
DLCAEYHNTQIH	8	0.718
FGPGPGWT	118	0.666
YYGPGPGYL	131	0.655
FAMGPGPGIR	320	0.618
TFGPGPGHSAWSHPQFGPGP	187	0.602
AGPGPG	302	0.561
ATGPGPGAA	341	0.546
GPGPG	161	0.543
HRGPGPG	221	0.526

### Population coverage

The distribution and expression of HLA alleles may vary across the world based on the difference between regions and ethnicities^43^. In addition, successful vaccine development demands the assessment of HLA allele distribution around the world population^44^. Therefore, this study was conducted in order to evaluate if the vaccine designed against SARS-CoV-2 will be effective for the world population. The selected epitopes in our study showed total world population coverage of 95.78% (Table 5). In addition, the epitopes showed 97.47%, 97.26%, 84.84%, 87.66% and 90.77% coverage in Europe, United States, China, South Asia and Oceania, respectively (Table 5) (Supplementary Fig. S5). The results suggest that the designed multi-epitope vaccine can be used to tackle SARS-CoV-2 globally.

**Table 5:** Population coverage of the selected epitopes of the vaccine construct, as predicted by IEDB server.

Population/area	Coverage	Average hit	pc90
World	95.78	4.29	1.78
Europe	97.47	4.69	2.14
United States	97.26	4.69	2.14
China	84.84	3.17	0.66
South Asia	87.66	3.1	0.81
Oceania	90.77	2.79	1.04

*pc* population coverage.

### Molecular docking analysis

#### Docking of the vaccine with TLRs

In order to generate a stable immune response, it is important for the vaccine to interact with target immune cell receptors. For studying such interactions, molecular docking studies were performed with Toll-like receptors. Toll-like receptors (TLRs) have a central role in innate immunity as they detect conserved pathogen-associated molecular patterns (PAMPs) on a range of microbes, including viruses, leading to innate immune activation and orchestration of the adaptive immune response^45^. TLR4 and TLR2 have also been implicated in the recognition of viral structural proteins leading to inflammatory cytokine production^46^. In addition, several studies on SARS-CoV have shown the importance of TLR4 and TLR2 in generation of an effective immune response^47–49^. Therefore, molecular docking studies of the vaccine candidate with TLR4/TLR2 were conducted.

#### Docking of the vaccine with TLR4

**Figure 6:**
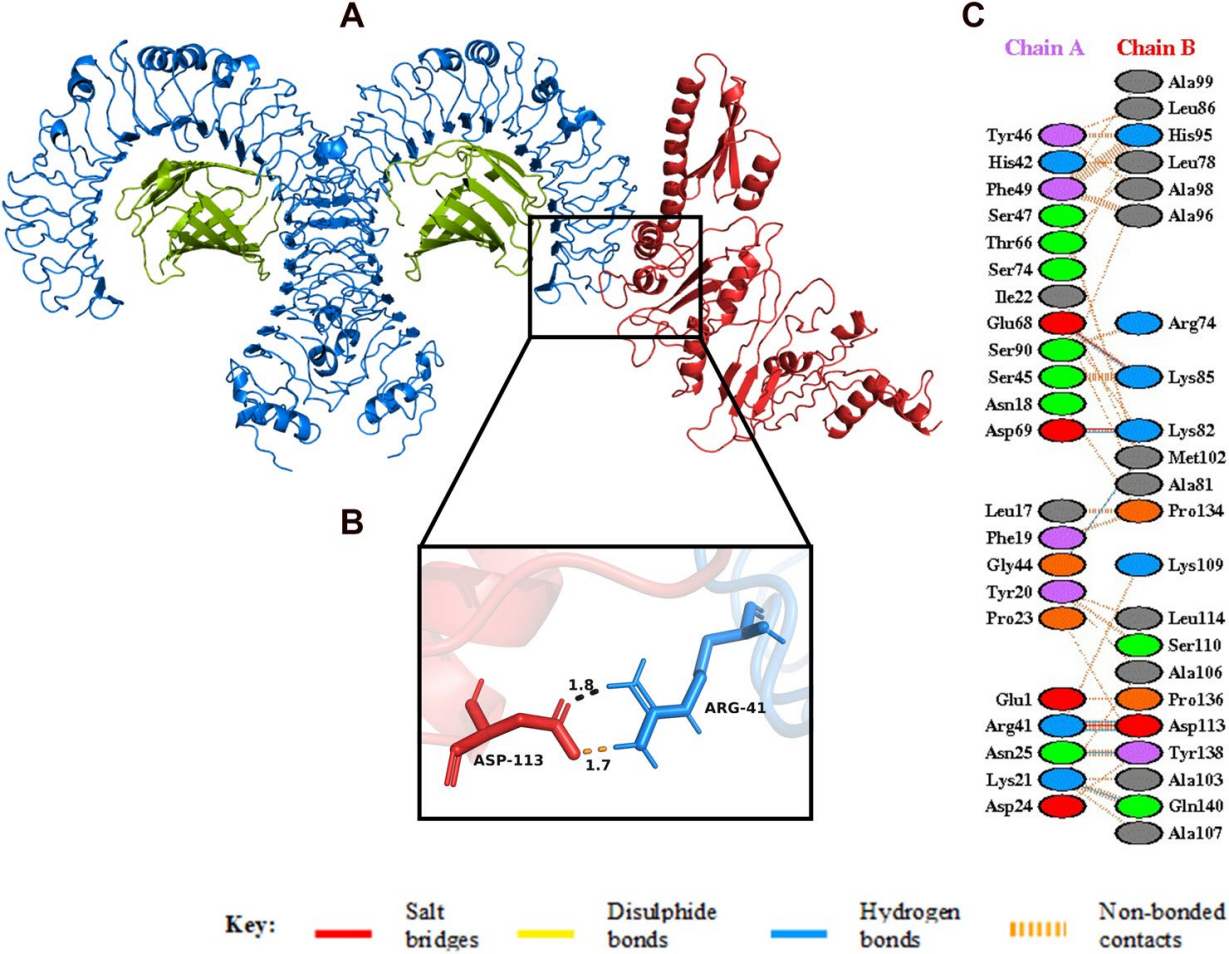
(**A**) Figure obtained after molecular docking, showing TLR4/MD2-vaccine docked complex. Vaccine construct is shown in red colour while TLR4 dimer is shown in blue colour and MD2 co-receptor shown in green colour. (**B**) Interacting residues between docked TLR4/MD2 tetramer (chain **A**) and vaccine (chain **B**). (**C**) Few prominent hydrogen bonds within vaccine-TLR4 complex are focused.

HADDOCK clustered 33 structures in 7 cluster(s), which represents 16.5% of the water refined HADDOCK generated models. The top cluster with the lowest HADDOCK score is the most reliable cluster of all. A representative model of the top cluster was subjected to further refinement using HADDOCK refinement server, where 20 structures were clustered into one cluster, resulting in 100% of the water refined models generated by HADDOCK. The statistics of the refined model are presented in the Table 6, and the structural analysis of the refined model is shown in Supplementary Fig. S7. The Haddock score of − 130.9 ± 10.1 suggest a good binding affinity between the vaccine and the receptor, negative score indicates better docking. A buried surface area (BSA) of 2,204.4 ± 22.4 Å^2^ indicates close proximity and a less water-exposed protein surface^50^. In addition, RMSD scores are also considered as an important parameter for evaluation of efficient docking studies, as it allows us to identify the complex with the lowest energy and least structural deviation. The low RMSD score of the docked complex (Table 6) indicates a good quality model. The predicted interaction of the amino acids and a detailed overview of the molecular docking are given in Supplementary Material 2 and Supplementary Fig. S8, respectively. Also, Ramachandran plot analysis was carried out for structural validation of the docked complex (Supplementary Fig. S6). The docked complex along with some prominent hydrogen bonds is shown in Fig. 6.

**Table 6:** Table showing statistics of best refined docked TLR4/MD2 and vaccine complex.

**Vaccine-TLR4**	
HADDOCK score (a.u)	− 130.9 ± 10.1
Cluster size	20
RMSD from the overall lowest-energy structure (Å)	0.3 ± 0.2
Van der Waals energy (kcal mol^−1^)	− 72.4 ± 1.3
Electrostatic energy (kcal mol^−1^)	− 238.9 ± 12.2
Desolvation energy (kcal mol^−1^)	− 10.9 ± 13.2
Restraints violation energy (kcal mol^−1^)	1.1 ± 0.44
Buried surface area (Å^2^)	2,204.4 ± 22.4

Smaller HADDOCK score represents strong protein interaction which is expressed in arbitrary units (a.u).

#### Docking of vaccine with TLR2

**Figure 7:**
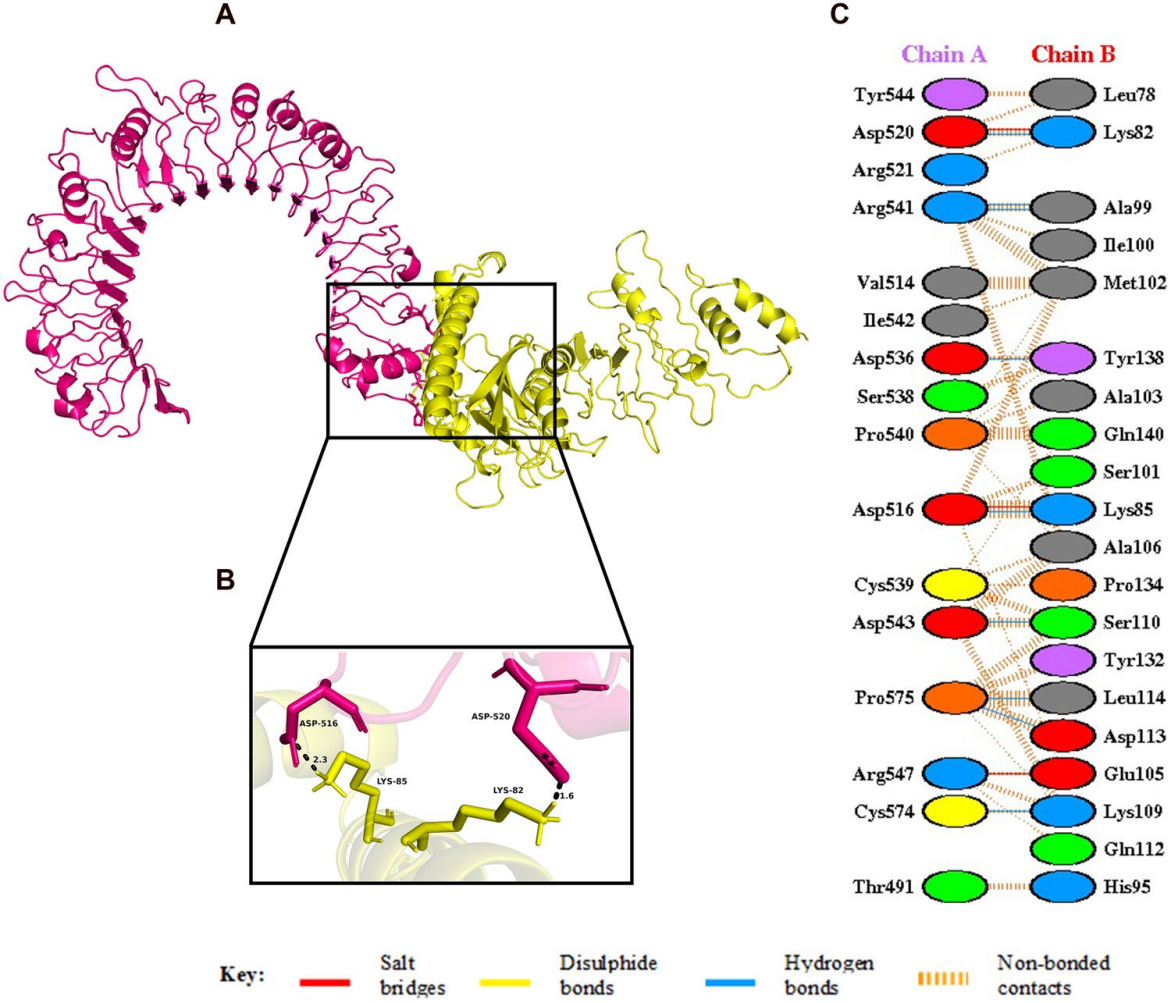
(**A**) Figure obtained after molecular docking, showing TLR2-vaccine docked complex. Vaccine construct is shown in yellow colour while TLR2 is shown in hot pink colour. (**B**) Interacting residues between docked TLR2 (chain **A**) and vaccine (chain **B**). (**C**) Few prominent hydrogen bonds within vaccine-TLR2 complex are focused.

HADDOCK clustered 80 structures in 11 cluster (s), which represents 40.0% of the water refined HADDOCK generated models. The structure with the lowest HADDOCK score was chosen as the top cluster. A representative model of the top cluster was subjected to further refinement using HADDOCK refinement server, where 20 structures were clustered into one cluster, resulting in 100% of the water refined models generated by HADDOCK. The statistics of the refined model are presented in the Table 7, and the structural analysis of the refined model is shown in Supplementary Fig. S10. There was a good binding affinity between the vaccine and the receptor which is evident from the negative HADDOCK score of − 112.0 ± 2.8^50^. The other docking scores as shown in Table 7 suggest a stable binding between the vaccine and the TLR2 receptor. The predicted interaction of the amino acids and a detailed overview of the molecular docking are given in Supplementary Material 3 and Supplementary Fig. S11, respectively. Also, Ramachandran plot analysis was carried out for structural validation of the docked complex (Supplementary Fig. S9). The docked complex along with some prominent hydrogen bonds is shown in Fig. 7.

**Table 7:** Table showing statistics of best refined docked TLR2 and vaccine complex.

**Vaccine-TLR2**	
HADDOCK score (a.u)	− 112.0 ± 2.8
Cluster size	20
RMSD from the overall lowest-energy structure (Å)	0.3 ± 0.2
Van der Waals energy (kcal mol^−1^)	− 73.2 ± 5.2
Electrostatic energy (kcal mol^−1^)	− 319.7 ± 32.7
Desolvation energy (kcal mol^−1^)	25.1 ± 4.3
Restraints violation energy (kcal mol^−1^)	0.0 ± 0.00
Buried surface area (Å^2^)	2094.7 ± 24.1

Smaller HADDOCK score represents strong protein interaction which is expressed in arbitrary units (a.u).

#### Docking of vaccine with MHC class I and class II receptors

The multi-epitope vaccine construct consisting of CTL and HTL epitopes interact with MHC class I and MHC class II receptors, forming epitope-MHC complex which activate the CTLs and HTLs required for immune response generation^51^. In order to study these interactions the Molecular Docking Analysis of the vaccine with MHC class I and class II receptors was performed.

#### Docking of vaccine with MHC class I receptor

**Figure 8:**
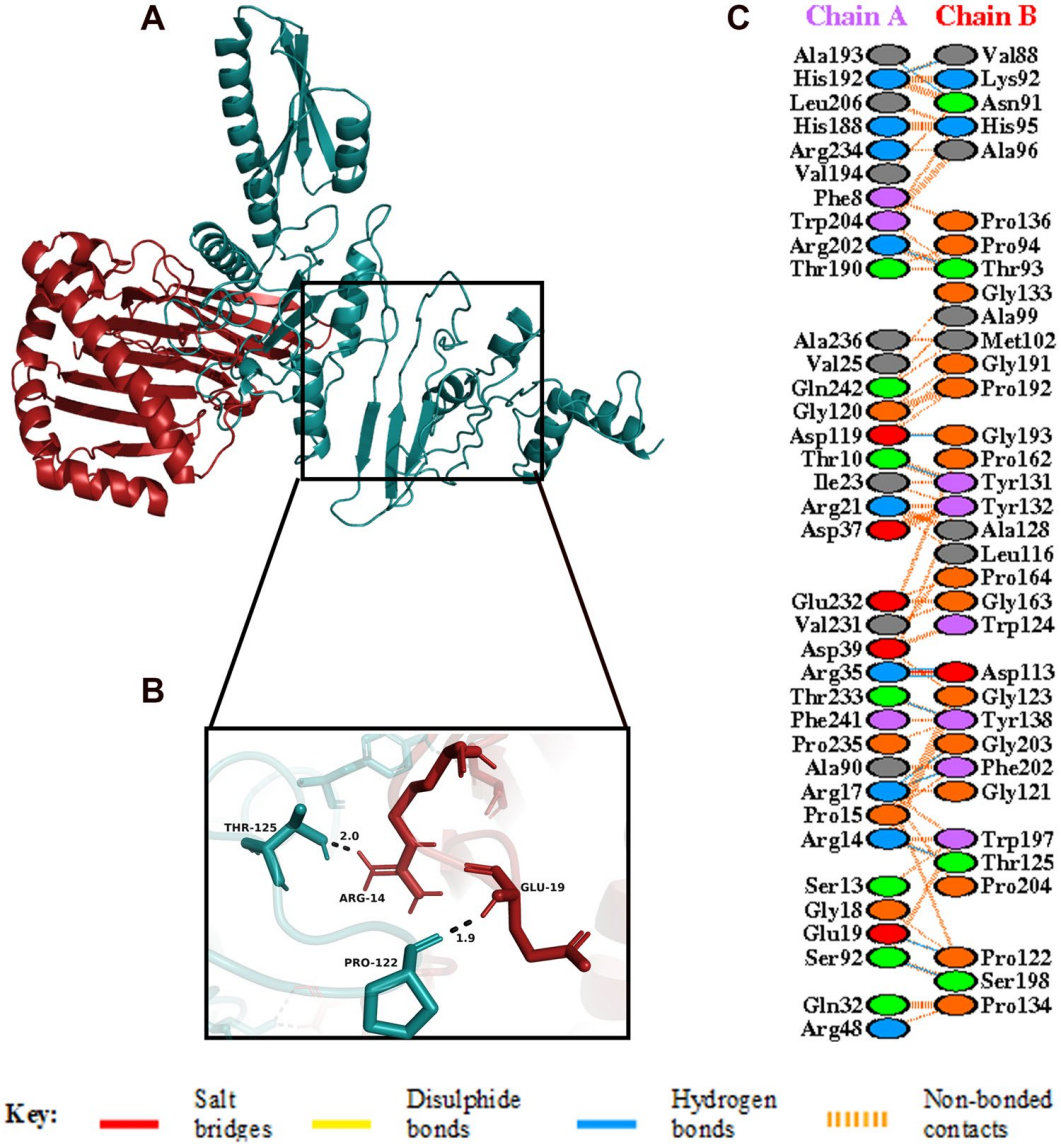
(**A**) Figure obtained after molecular docking, showing MHC I-vaccine docked complex. Vaccine construct is shown in deep teal colour while MHC I is shown in fire brick colour. (**B**) Interacting residues between docked MHC I (chain **A**) and vaccine (chain **B**). (**C**) Few prominent hydrogen bonds within vaccine-MHC I complex are focused.

HADDOCK clustered 120 structures in 12 cluster(s), which represents 60.0% of the water refined HADDOCK generated models. The structure with the lowest HADDOCK score was chosen as the top cluster. A representative model of the top cluster was subjected to further refinement using HADDOCK refinement server, where 20 structures were clustered into one cluster, resulting in 100% of the water refined models generated by HADDOCK. The statistics of the refined model are presented in the Table 8, and the structural analysis of the refined model is shown in Supplementary Fig. S13. The statistics of the refined docked complex indicates a strong binding affinity between the vaccine and MHC class I receptor. The low HADDOCK score of − 214.7 ± 4.1 indicates the docking to be effective and, the lower value of RMSD (Table 8) suggest stability of the docked complex. The predicted interaction of the amino acids and a detailed overview of the molecular docking are given in Supplementary Material 4 and Supplementary Fig. S14, respectively. Also, Ramachandran plot analysis was carried out for structural validation of the docked complex (Supplementary Fig. S12). The docked complex along with some prominent hydrogen bonds is shown in Fig. 8.

**Table 8:** Table showing statistics of best refined docked MHC class I and vaccine complex.

**Vaccine-MHC I**	
HADDOCK score (a.u)	− 214.7 ± 4.1
Cluster size	20
RMSD from the overall lowest-energy structure (Å)	0.3 ± 0.2
Van der Waals energy (kcal mol^−1^)	− 138.5 ± 2.2
Electrostatic energy (kcal mol^−1^)	− 156.3 ± 16.9
Desolvation energy (kcal mol^−1^)	− 45.0 ± 5.8
Restraints violation energy (kcal mol^−1^)	0.0 ± 0.00
Buried Surface Area (Å^2^)	3,585.9 ± 60.3

Smaller HADDOCK score represents strong protein interaction which is expressed in arbitrary units (a.u).

#### Docking of vaccine with MHC class II receptor

**Figure 9:**
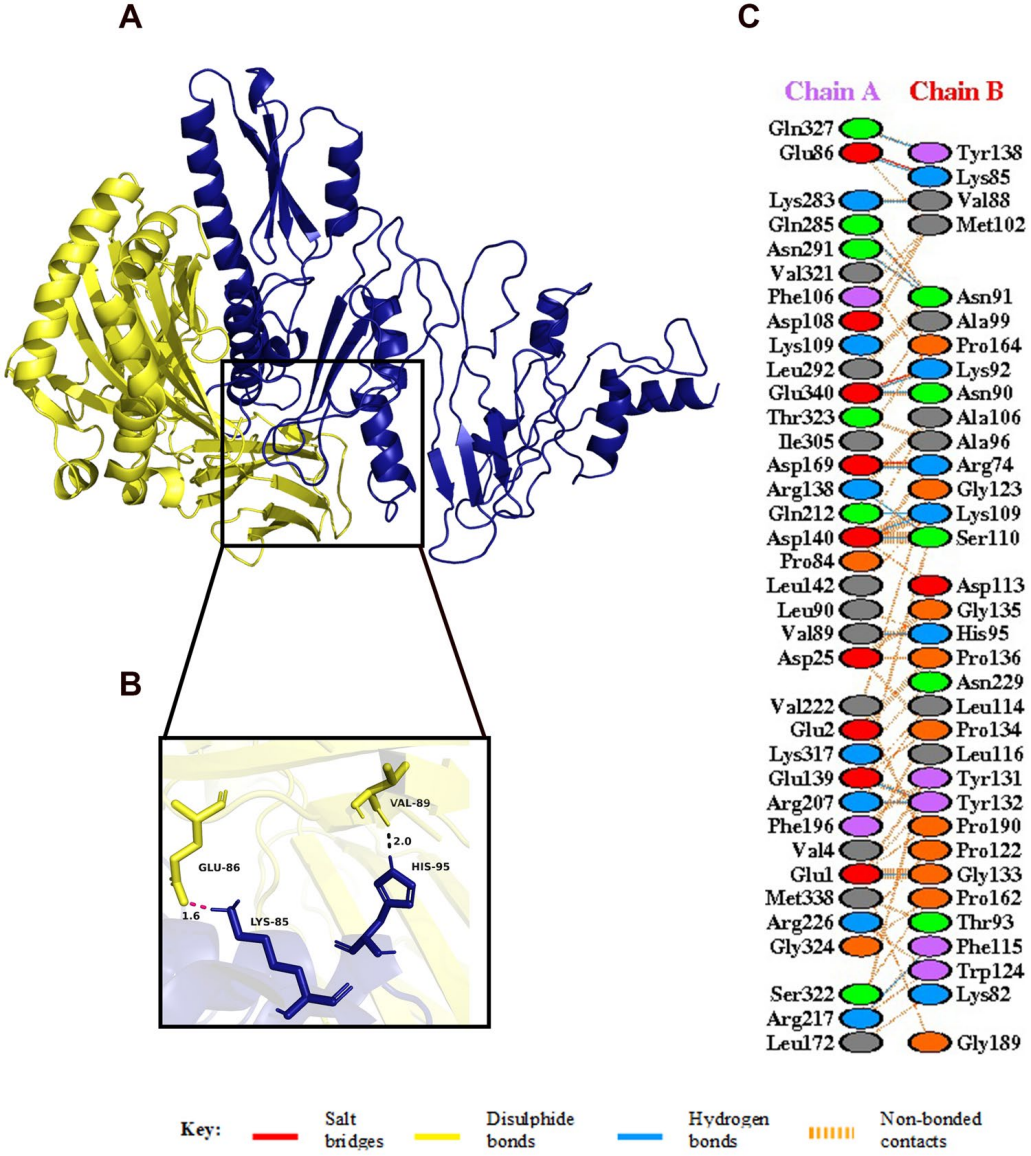
(**A**) Figure obtained after molecular docking, showing MHC II-vaccine docked complex. Vaccine construct is shown in blue colour while MHC II is shown in yellow colour. (**B**) Interacting residues between docked MHC II (chain **A**) and vaccine (chain B). (**C**) Few prominent hydrogen bonds within vaccine-MHC II complex are focused.

HADDOCK clustered 64 structures in 9 cluster (s), which represents 32% of the water refined HADDOCK generated models. The structure with the lowest HADDOCK score was chosen as the top cluster. A representative model of the top cluster was subjected to further refinement using HADDOCK refinement server, where 20 structures were clustered into one cluster, resulting 100% of the water refined HADDOCK generated models. The statistics of the refined model as presented in Table 9 suggest a good docking score (because of low HADDOCK score), thereby confirming a stable and efficient docking of the vaccine and the MHC class II receptor. In addition, the structural analysis of the refined model is shown in Supplementary Fig. S16. The predicted interaction of the amino acids and a detailed overview of the molecular docking are given in Supplementary Material 5 and Supplementary Fig. S17, respectively. Also, Ramachandran plot analysis was carried out for structural validation of the docked complex (Supplementary Fig. S15). The docked complex along with some prominent hydrogen bonds is shown in Fig. 9.

**Table 9:** Table showing statistics of best refined docked MHC class II and vaccine complex.

**Vaccine-MHC II**	
HADDOCK score (a.u)	− 212.1 ± 2.2
Cluster size	20
RMSD from the overall lowest-energy structure (Å)	0.3 ± 0.2
Van der Waals energy (kcal mol^−1^)	− 132.5 ± 3.2
Electrostatic energy (kcal mol^−1^)	− 394.9 ± 42.3
Desolvation energy (kcal mol^−1^)	− 0.6 ± 4.4
Restraints violation energy (kcal mol^−1^)	0.2 ± 0.27
Buried surface area (Å^2^)	4,276.9 ± 43.1

Smaller HADDOCK score represents strong protein interaction which is expressed in arbitrary units (a.u).

### Binding affinity analysis

The binding affinity of a complex, or the Gibbs free energy (∆G) in terms of thermodynamics, is a crucial quantity for determining whether an interaction will actually occur or not in the cell at specific conditions^52^. Therefore, the binding affinity of the 4 docked complexes was analysed using PRODIGY web server. The ΔG values for the vaccine-TLR4, vaccine-TLR2, vaccine-MHC class I and vaccine-MHC class II receptor was found to be − 10.3 kcal mol^−1^, − 11.2 kcal mol^−1^, − 13.5 kcal mol^−1^, − 16.0 kcal mol^−1^, respectively (Table 10). The results revealed that all of the 4 dockings were energetically feasible, as indicated by the negative values of Gibbs free energy (ΔG). The dissociation constant (K_d_) of the docked complexes are shown in Table 10.

**Table 10:** Binding affinities of the docked complexes of the vaccine with TLR4, TLR2, MHC I and MHC II, as predicted by PRODIGY server.

Complexes	Gibbs free energy (kcal mol^−1^)	Kd (M)
Vaccine-TLR4	− 10.3	5.3E-08
Vaccine-TLR2	− 11.2	1.3E-08
Vaccine-MHC class I receptor	− 13.5	2.9E-10
Vaccine-MHC class II receptor	− 16	5.0E-12

### Energy minimization and molecular dynamics simulation of the vaccine construct

**Figure 10:**
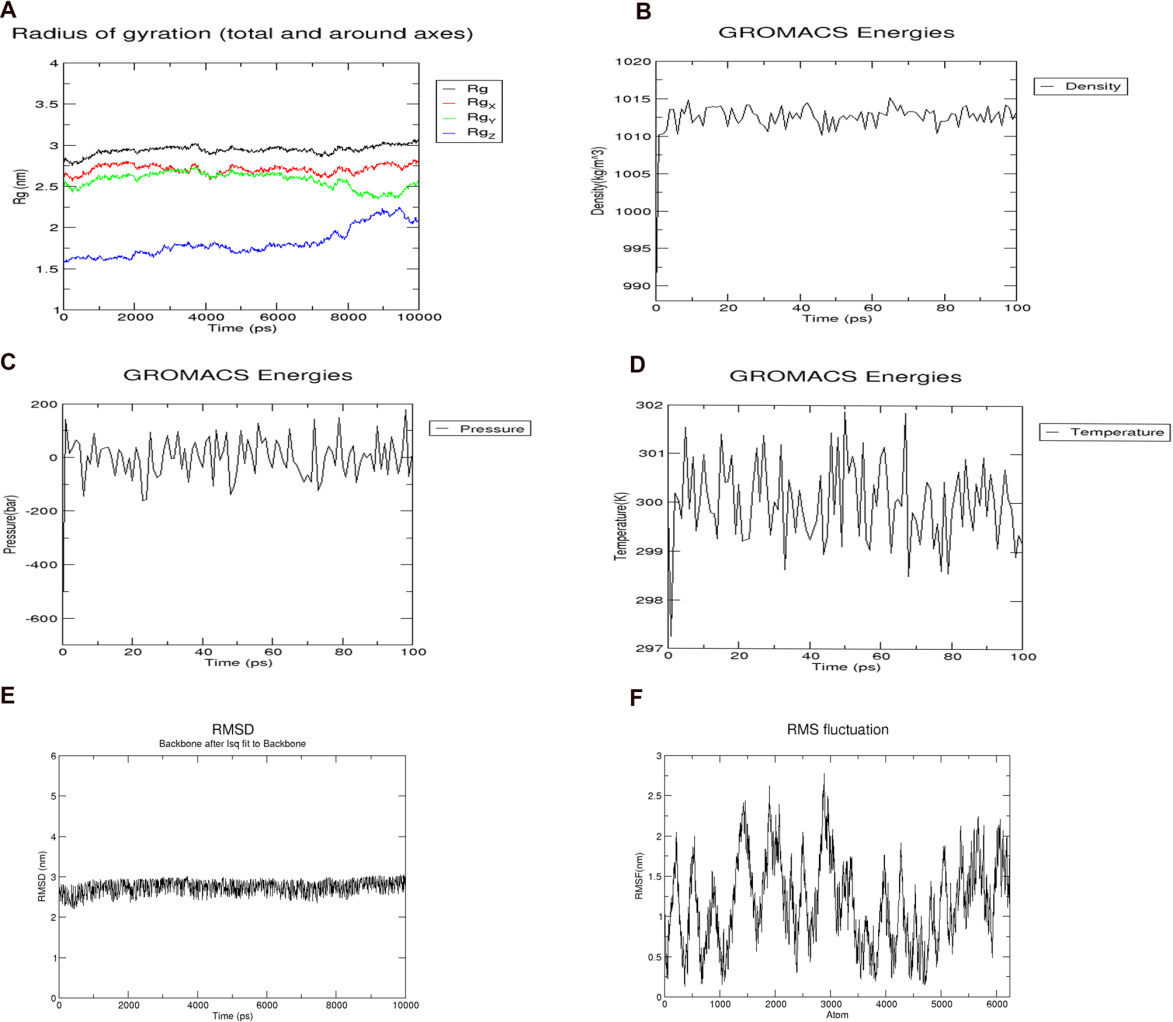
(**A**) Radius of Gyration plot showing compactness of the vaccine around its axes. (**B**) Graph showing density of the system during simulation. (**C**) Graph showing the pressure of the system during simulation. (**D**) Graph showing the equilibrated temperature during energy minimisation. **(E**) RMSD plot of the vaccine construct indicating stability. (**F**) RMSF plot of the vaccine construct showing high fluctuations, indicating high flexibility.

Molecular dynamics simulation (MDS) is essential to determine the stability of a protein at different thermobaric conditions. In order to check the protein stability, energy minimization for the vaccine was conducted using the steepest descent algorithm of GROMACS. Once, the force reaches < 1000 kJ/mol, the protein is considered to be energy minimised. The energy minimisation for the vaccine construct was conducted for 2,262 steps where the force reached < 1000 kJ/mol. The potential energy of the system was computed to be − 3.0e + 06 kJ/mol with a total drift of − 3.8 × 10^5^ kJ/mol and the average potential energy was − 2.9e + 06 kJ/mol. After 50,000 steps of NVT the average temperature was 299.8 K with a drift of 1.0 K (Fig. 10D). The average density of the system computed was 1,012.5 kg/m^3^ with a total drift of 1.3 kg/m^3^ (Fig. 10B). The pressure of the system was found to be 1.6 bar with a total drift of 4.2 bar (Fig. 10C). Trajectory analysis was performed after a simulation period of 10 ns in order to check the stability and flexibility of the vaccine candidate. The plot for the radius of gyration showed the compactness of the protein around its axes (Fig. 10A). A plot of RMSD backbone revealed very mild fluctuations, indicating the stability of the vaccine over time (Fig. 10E). The high peaks in the RMSF plot suggested a high degree of flexibility in the vaccine construct (Fig. 10F).

### Reverse translation, codon optimization and in silico cloning of the vaccine-

**Figure 11:**
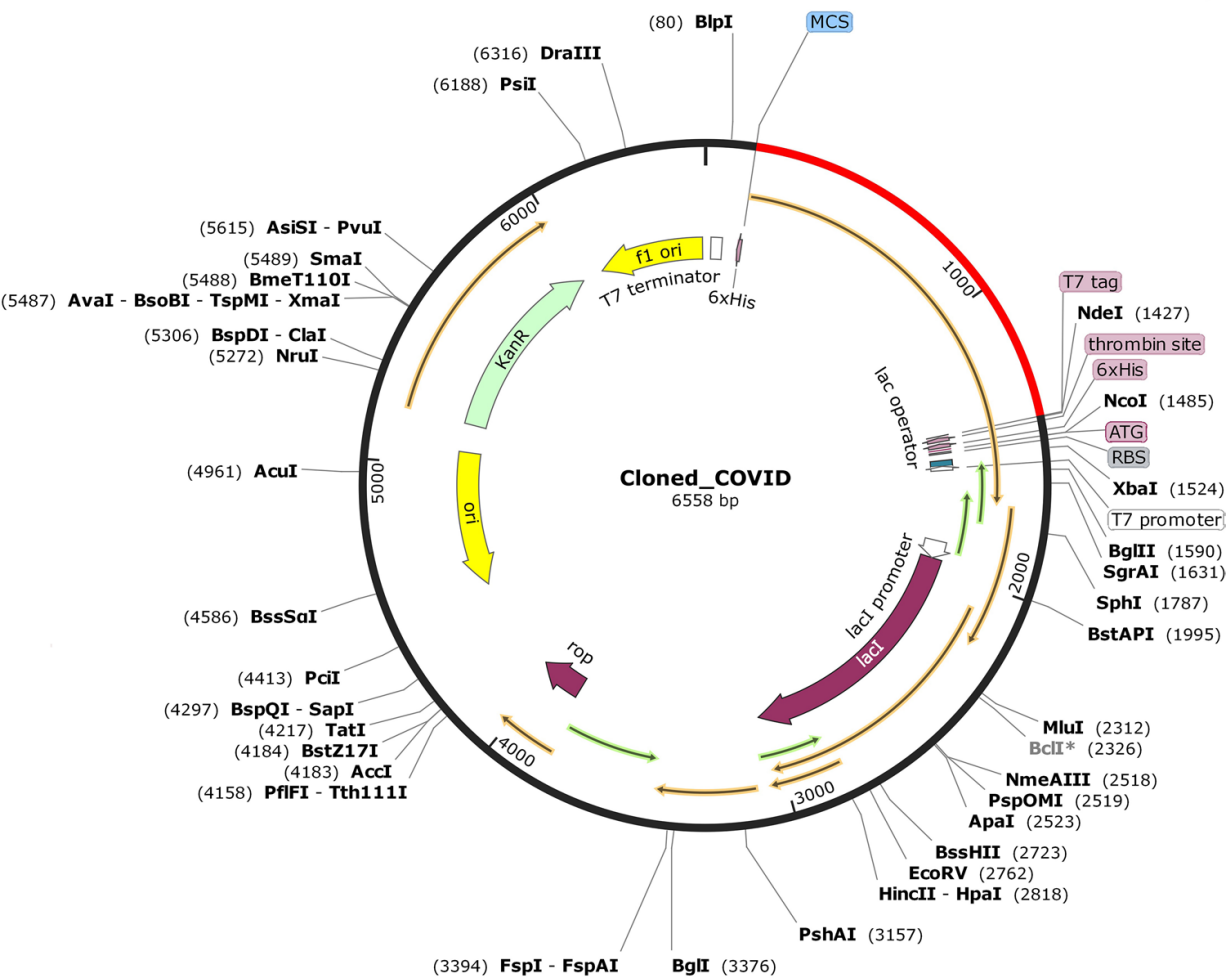
In silico restriction cloning. The red coloured portion represents the codon optimised multi-epitope vaccine inserted into the pET-28a (+) expression vector which is represented in black colour.

In silico cloning was performed so that the vaccine candidate could be expressed into the *E. coli* expression system. Therefore, it was necessary to optimize the codon respective to the vaccine construct as per the usage of *E. coli* expression system, in order to ensure efficient translation and increased protein production. For optimizing the codon usage of the designed vaccine construct for maximal protein expression in *E. coli* K-12 strain, JCat tool was used. The generated cDNA sequence after codon optimization was 1,266 nucleotides long (Supplementary Material SM6). Generally, a codon adaptation index (CAI) value > 0.8 and the GC content between 30 and 70% are considered for a good protein expression in the host system. Our vaccine had a codon adaptation index (CAI) of 1.0 and GC content of the reverse translated vaccine was 58.53%. These results support a proficient expression of the designed vaccine in *E. coli* K-12 strain. Finally, the recombinant plasmid was designed by inserting the adapted codon sequences into pET-28a (+) vector using SnapGene software, computationally (Fig. 11). This study was conducted in order to design an effective cloning strategy for the candidate vaccine.

### Immune simulation

**Figure 12:**
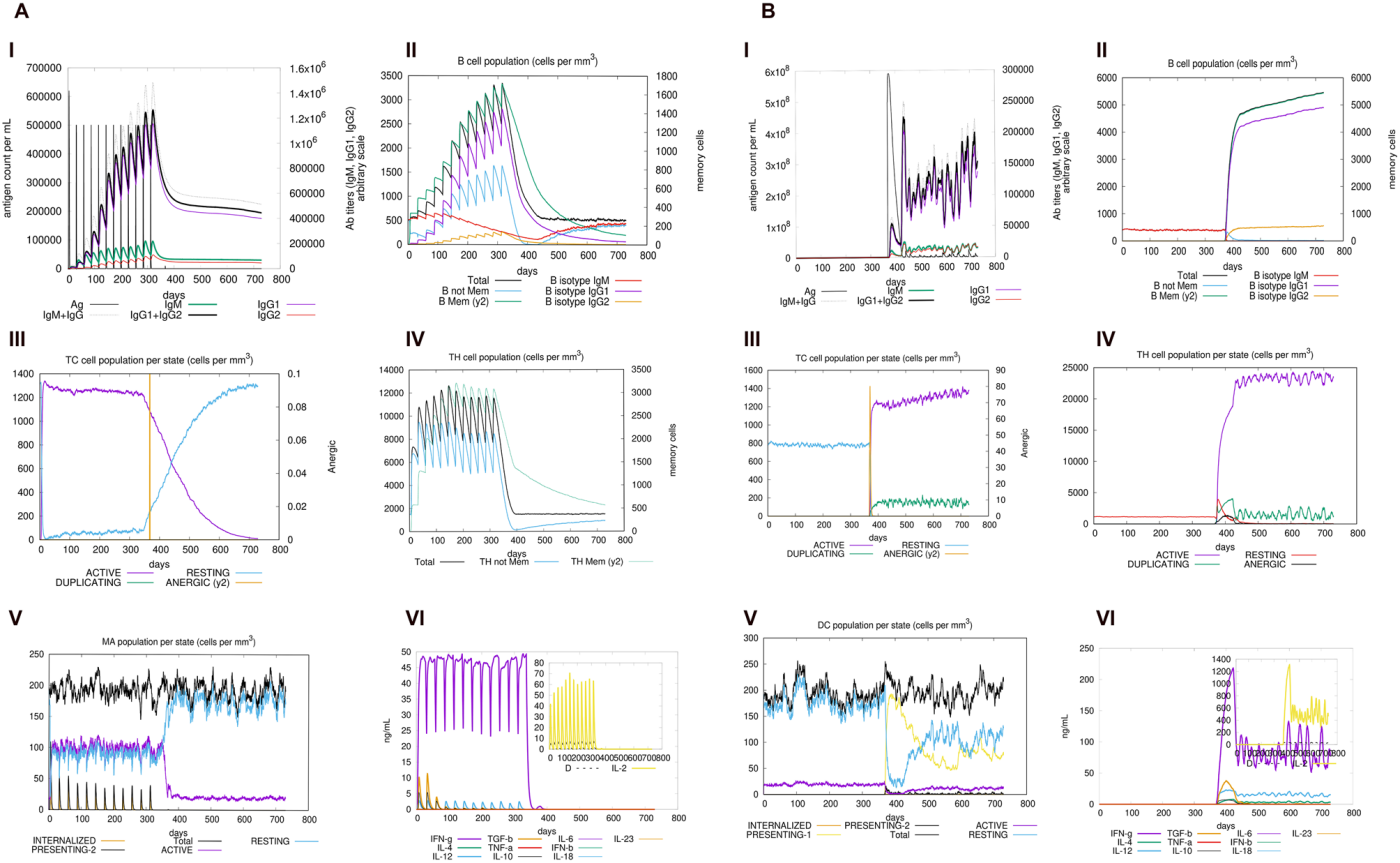
(**A**) The vaccine is injected in 12 doses on a period of 12 months. (**Ai**) shows the rise of antigen concentration and relative antibodies responses. The infection with a live-replicating virus is performed two months after last vaccine inoculation. The virus is cleared with no delay due to the presence of protective IgGs thus showing the efficacy of the vaccination. (**Aii**) shows the corresponding count of antibody generating plasma cells while (**Aiii–Av**) show the activity (detailed in terms of counts and activation states) of cytotoxic T cells, helper T cells and macrophages respectively. (**Avi**) shows the cytokine concentration during the whole simulated period evidencing, in particular, a high level of pro-inflammatory IFN-g, TNF-b and IL-10 evidencing the reaction to the vaccine (**B**) Shows the control case of a simulation of infection by means of one injection of a "live-replicating” virus and without prior vaccination. The virus here is injected at the same time as in the previous simulation that is shown in panels (**Ai–Avi**) to make it easy to compare the various plots. In this case we observed the unstopped growth of the viral load (**Bi**) attesting that a naïve (yet present) host response (**Bii–Bvi**) is not able to eliminate the virus thus confirming the efficacy of the vaccine in preventing the viral explosion.

An in silico immune response was generated using the C-IMMSIM immune server in order to assess the immunogenic profile of multi-epitope vaccine^53^ (Fig. 12). The secondary and tertiary responses generated by the simulation were significantly higher when compared to the primary response. The secondary and tertiary responses revealed a decrease in the antigenic concentration with normal high levels of immunoglobulin activity (i.e., IgG1 + IgG2, IgM, and IgG + IgM antibodies). In addition, multiple long lasting B cell isotypes were found, suggesting possible isotype switching potentials and memory formation (Fig. 12Aii, Supplementary Fig. S18). The TH (helper) and TC (cytotoxic) cell populations also showed a similar higher response with the pre activation of TCs during vaccination (Fig. 12Aiv, Aiii) (Supplementary Fig. S18). The NK (natural killer) and dendritic cell activity was found to be consistent along with higher macrophage activity (Supplementary Fig. S18) demonstrated during the exposure (Fig. 12Av). The generation of a good immune response was supported by the high levels of IFN-γ and IL-2 elicited in the simulation. After the vaccination, an injection of a “live-replicating virus” was simulated at around day 366 in order to check the efficacy of the vaccine. The antigen graph (Fig. 12Ai) shows that after the vaccination, when a live replicating virus is injected, the antigenic surge is virtually absent, indicating an effective immune response mainly due to the protective action of high concentration of specific antibodies. This outcome should be compared with a control simulation that was also performed consisting of an injection of the live virus after 1 year, without prior vaccination. In this case, results indicate that without prior vaccination the host is unable to contain the antigen, though an inefficacious immune response is generated (Fig. 12B, Supplementary Fig. S19). In another control experiment a vaccine construct was designed utilizing randomly generated sequences to see its effect on immune response. As expected, the Immune Simulation results obtained from the randomly generated sequence shows the absence of any immune response thereby confirming the failure of vaccination (Data not shown). The simple reason for this is the lack of antigenic peptides in the random sequence, which in the simulation translates in the absence of antigenic presentation by professional antigen presenting cells.

## Discussion

SARS-CoV-2 has been declared as a global pandemic by World Health Organization affecting people of all age groups. World Health Organization’s announcement on COVID-19 as a global public health emergency has encouraged researchers to develop therapeutics such as drug candidates and vaccines against the disease^54^. The cost effective and time saving immunoinformatic approaches have already helped the researchers to predict potential antigenic epitopes required for the development of a multi-epitope vaccine candidate^55–58^. The distinctive concept of multi-epitope vaccine design as compared to classical single-epitope based vaccine is that, the screening of viral genome to identify immunogenic epitopes results in the elicitation of a highly targeted immune response without any reversal of viral pathogenesis^59^.

In this study, we aim at designing a multi-epitope, prophylactic vaccine targeting the spike protein of SARS-CoV-2, which is one of the major determinants of antigenicity and viral entry into the host cell^3^. Several computational tools were used to construct a multi-epitope vaccine, which has the ability to generate both humoral and cell mediated immunity. The multi-epitope vaccine elicits immune responses based on short immunogenic sequences instead of large proteins or whole genome which is typically used for recombinant vaccine technology. Thus, this approach avoids the excess antigenic load as well as allergenic responses in the host^28,60,61^. The analysis of the entire spectrum of possible antigens can be carried out using immunoinformatics and molecular modelling in order to examine the potential binding with host proteins^55,62–65^. In addition, these multi-epitope vaccines have advantages over traditional and single-epitope vaccines due to the following unique features: (i) multiple MHC Class I and Class II epitopes can be recognized by TCRs from various T cell subsets, (ii) overlapping CTL, HTL and B cell epitopes have the capacity to activate humoral and cellular immune responses simultaneously, (iii) linking an adjuvant to the vaccine ensures a long lasting immune response with enhanced immunogenicity, (iv) the in vitro antigen expression complications as well as the difficulty of culturing the pathogens can also be avoided^66–74^. Designing of multi-epitope vaccines is an emerging area which has already gained importance, and the vaccines designed by this approach, have not only shown in vivo efficacy with protective immunity^75–77^ but also entered phase-I clinical trials^70, \71,78,79^

The present study utilized the potential immunogenic epitopes identified from the SARS-CoV-2 spike protein to construct the multi-epitope vaccine with Cholera Toxin B (CTB) as an adjuvant along with appropriate linkers. Cholera Toxin B, which has been proven to act as a potential viral adjuvant, is linked at the N-terminal of the vaccine construct^80–82^. Glycine rich linker, such as GPGPG, was preferred to link the screened epitopes as it enhances the solubility and enable the adjoining domains to be accessible and act freely^83^. Various immunological filters were used to screen the predicted CTL and HTL epitopes: the epitopes must be antigenic and immunogenic, should bind with multiple MHC class I and MHC class II alleles (promiscuous), and must have overlapping CTL and HTL epitopes. A similar approach was used by Bazhan and his co-workers, where they have designed a T-cell multi epitope vaccine against Ebola virus. The T-cell epitopes were predicted using IEDB—Immune Epitope Database and the vaccine candidate constructed using the suitable epitopes were found to be immunogenic when expressed in mice^84^. Our designed vaccine was predicted to be non-allergen using AllerTOP v.2.0 server which was further verified by AllergenFP v.1.0^85–88^. The other physicochemical properties of the vaccine were analysed using ProtParam tool offered by ExPASy server^89^. The molecular weight of the construct was 44.15 kDa and the instability index was evaluated to be 31.04 which classify the vaccine to be stable. Generally, a protein whose instability index is lesser than 40 is predicted to be stable and values above that predicts the protein as unstable^89^. The theoretical pI of the vaccine was calculated to be 9.96. The GRAVY index of the vaccine was − 0.088, (lower the GRAVY score, better is the solubility), which is reflective of the vaccine’s polar nature and its effective interaction with water, suggesting high solubility^90^. The aliphatic index of 78.74 indicated the protein to be thermostable^91^. The half-life of the vaccine was evaluated to be 30 h (mammalian reticulocytes, in vitro), > 20 h (yeast, in vivo) and > 10 h (*Escherichia coli*, in vivo) which indicates the time taken by the protein to reach 50% of its concentration after its synthesis in the cell. Similarly, Foroutan and his colleagues have also used the same array of in silico analysis in order to assess the allergenecity and physicochemical properties of their designed vaccine candidate against *Toxoplasma gondii*^92^. They have also performed laboratory validation of their vaccine candidate, which revealed that the multi-epitope vaccine was able to trigger strong humoral and cellular responses in mice^92^. The physicochemical properties predicted in our study were comparable to those predicted by Foroutan et al., in their recently published work^92^. In fact, the instability index and aliphatic index of our vaccine candidate was found to be better when compared to the values reported by Foroutan et al.^92^. The structural validation of our vaccine construct performed by Ramachandran plot analysis using RAMPAGE showed that 96.4% of residues were in favoured region, 2.9% were in the allowed region and only 0.4% of the residues were placed in the outlier region thereby, validating the tertiary structure of the vaccine. The ERRAT score of 74.29 further validated the overall quality of our vaccine and Z-score assessment by ProSA web server revealed a score of − 8.1, indicating that the protein falls in the plot which consists of the Z-scores of the already determined structures solved by NMR and X-ray crystallographic experiments^36^.

The spike glycoprotein of SARS-CoV-2, which is one of the structural components of the virus, should be recognized by the Toll-Like Receptor 4 (TLR4) and Toll-Like Receptor 2 (TLR2) expressed in the plasma membrane of the cells^45,93,94^. Human Toll-Like Receptor 4 (TLR4) is expressed in various types of immune cells like monocytes, macrophages, granulocytes and immature dendritic cells^95^. A direct interaction between TLR4 and CTB is responsible for the activation of TLR4 by CTB^96^. This conclusion is strengthened by the fact that the capacity of CTB to induce inflammatory response is lost in TLR4-deficient macrophages^96^. The ELISA-based assays have demonstrated that CTB is able to induce NF-κB activation in TLR4 receptor cells by binding to it directly^96^. In addition, TLR2 is also associated with recognition of viral envelop glycoprotein^93^. The myeloid differentiation factor 88 (MyD88) acts as the primary adaptor for the core TLR2 signalling pathway, which results in NF-κB and mitogen-activated protein kinase (MAPK) activation, leading to secretion of a core panel of cytokines^93^. The interaction pattern of the vaccine with TLR4 and TLR2 was analysed by Molecular Docking Studies (Figs. 6, 7). The docking analysis of TLR4 and the vaccine construct showed that there are 3 salt bridges and 7 hydrogen bonds formed during this interaction. The docked complex shows that the salt bridges were formed between Arg41, Glu68, Asp69 of TLR4 and Asp113, Lys85, Lys82 of vaccine, respectively. Similarly, docking analysis of TLR2 and the vaccine construct also showed that there are 3 salt bridges and 9 hydrogen bonds formed during the interaction. The salt bridges formed in this case were between Asp516, Asp520, Arg547 of TLR4 and Lys85, Lys82, Glu105 of our vaccine, respectively. Several studies on SARS-CoV have shown the importance of TLR4 and TLR2 in generation of an effective immune response. In one of the studies, Totura et al. has demonstrated that TLR4 deficient mice are more susceptible to SARS-CoV infection than the wild type mice^47^. Similarly, Hu et al. conducted a study where they have seen the expression and regulation of Toll-Like Receptors in human monocytic cell upon SARS-CoV infection^48^. The results obtained from their study indicate that the expression of TLR4/TLR2 is upregulated at 24 h after SARS-CoV infection, suggesting its importance in the generation of immune responses^48^. In addition, Dosch et al. have shown that TLR2 present on human macrophages interacts with S protein of SARS-CoV to induce IL-8 production in body^49^. The sensitized TLR2 triggers the release of IL-8 which is an important chemokine, necessary for generating an innate immune response^49^.

The molecular dynamics simulation of the vaccine construct for 10 ns showed that there were very mild fluctuations in the RMSD graph, indicating the vaccine’s stability (Fig. 10). The RMSF graph showed regions with high peaks, indicating the high flexibility of the vaccine construct (Fig. 10). The molecular dynamics simulation (MDS) is one of the most important steps used to check the stability of the vaccine by simulating the vaccine under in vivo conditions. RMSD and RMSF data obtained from our MDS is similar to the studies done by other research groups, where they have checked the stability and flexibility of the vaccine candidate mimicking the in vivo conditions^28,87,88^. To assure an effective expression in *E. coli* host, codon optimization of the designed vaccine was performed and the linear vaccine construct was reverse translated into its specific cDNA sequence. The GC content of it was recorded as 58.53%, therefore showing the possibility of efficient expression of the vaccine candidate in *E. coli* host. Further, insertion of the vaccine in the expression vector pET-28a (+) for in silico cloning was performed so that the vaccine can be expressed in bacterial system. A similar approach was used by Foroutan et al. in order to optimize the codon of their designed vaccine before its in vitro expression^92^. The immune simulation studies confirmed that our designed vaccine was able to elicit specific immune responses required to clear the antigen on secondary exposure (Fig. 12), after the final injection. Our immune simulation study was in fact better than the recently published work on multi-epitope vaccine candidate against SARS-CoV-2 where there was no live replicating virus injected after the vaccination in order to check its effectiveness on a secondary exposure with the antigen^97^.

Similarly, the immunoinformatic strategy of vaccine designing has recently been applied for designing multi-epitope vaccines against *Pseudomonas aeruginosa*^98^, *Klebsiella pneumoniae*^88^, Dengue^99^, Nipah virus^100^, Hendra virus^101^ and Malaria^102^. In addition, similar approach has also been applied for developing vaccine against cancerous antigens^28,103^. The CTL, HTL and IFN-γ epitopes included in the vaccine has the capacity to trigger the stimulation of host's respective immune cells which in turn can cause the activation of other immune cells via complex signalling.

## Materials and methods

### Sequence retrieval and phylogenetic tree construction-

The VIPR database (https://www.viprbrc.org/brc/home.spg?decorator=vipr) was used to retrieve the spike glycoprotein sequences of 7 coronaviruses (HCoV-NL63, HCoV-229E, HCoV-0C43, HKU-1, MERS-CoV, SARS-CoV and SARS-CoV-2) which have previously infected the human population. In addition, spike glycoprotein sequences of different strains of SARS-CoV-2, isolated from 19 different countries (China, Japan, USA, Australia, Finland, Sweden, India, Colombia, Taiwan, Pakistan, Italy, Israel, Iran, Iran, Vietnam, Peru, Brazil, Spain, Nepal and South Korea) around the globe were also retrieved from the VIPR database. Two phylogeny trees were constructed and for both the trees, the MUSCLE tool^104^ was used in order to align the glycoprotein sequences and the alignment file was used to construct the phylogenetic trees with default parameters and 1,000 bootstrap replicates, using the Neighbour Joining algorithm of MEGA 7.0.14^105^

### T cell epitope prediction

#### CTL epitope prediction

9-mer long CTL epitopes were predicted using NetCTL 1.2 server (https://www.cbs.dtu.dk/services/NetCTL/), recognized by the HLA Class I supertypes which are commonly occurring in human population, i.e., A1, A2, A3, A24, A26, B7, B8, B27, B39, B44, B58 and B62^106^. In the NetCTL 1.2 server, the thresholds were set at 0.15, 0.05 and 0.75 for distinctive parameters such as proteasomal C-terminal cleavage, Transporter Associated with Antigen Processing (TAP) and epitope recognition, respectively. NetCTL supports epitope prediction with 54–89% sensitivity and 94–99% specificity. Also, the epitopes recognized by other HLA Class I alleles were detected by Immune Epitope Consensus (IEDB) tool (https://tools.iedb.org/mhci/)^107^.

#### HTL epitope prediction

15-mer long HTL epitopes were predicted using NetMHCII pan 3.2 server (www.cbs.dtu.dk/services/NetMHCIIpan/), which had an affinity to class II HLA alleles^108^. The predicted peptides were classified as strong, intermediate and non-binders with threshold value set at 2, 10 and > 10% respectively, based on the idea of percentile rank as given by NetMHCII pan 3.2 server.

The epitopes were screened on the basis of antigenicity as well as immunogenicity as predicted by VaxiJen v2.0 and IEDB class I immunogenicity web servers, respectively^109,110^. The 3D structure of the spike glycoprotein was modelled using I-TASSER in order to visualize the selected epitopes on the protein surface^111–113^.

### B cell epitope prediction-

The ElliPro tool (https://tools.iedb.org/ellipro/) from IEDB server was used for predicting linear and conformational/discontinuous B cell epitopes with default parameters^114^.

#### IFN-γ epitope prediction

For both humoral and innate immunity, IFN-γ plays important role in antiviral, anti-tumour and immune regulatory activities. Hence, IFN-γ inducing epitopes are important for designing a potential multi-epitope vaccine. From the target protein, IFNepitope server (https://crdd.osdd.net/raghava/ifnepitope/) was used to predict out the IFN-γ epitopes^115^. The server has a maximum accuracy of 81.39% and various approaches such as machine learning strategy, motive-based analysis and accuracy hybrid approach is used for the prediction of the epitopes.

### Population coverage

The IEDB population coverage analysis tool (https://tools.iedb.org/population/) was used in order to check if the epitopes of the designed vaccine had effectively covered the entire world population^44^. As, SARS-CoV-2 is a global pandemic the population coverage was checked for the total world population, United States, Europe, China, South Asia and Oceania. The default parameters were used and the coverage was checked against the HLA class I and HLA class II binding alleles.

### Multi-epitope vaccine construct, structural modelling and validation

The screened CTL, HTL and IFN-γ inducing epitopes from the target glycoprotein were together linked by glycine-proline rich GPGPG linkers. In addition, Cholera Toxin B (CTB) adjuvant was added by EAAAK linker to the N-terminal of the vaccine construct as it can induce regulatory immune responses. trRosetta was used to generate the 3D model of linear vaccine construct^116^. The tertiary structure was validated using ERRAT score^38^ followed by ProSA-web analysis^36^. ProSA-web validates the structure based on Z-score predicted. Further, the overall quality of the generated model of vaccine was determined by Ramachandran plot analysis using RAMPAGE server^117^.

### Physicochemical properties of the vaccine construct-

VaxiJen v2.0^109^ was used to check the antigenicity of the vaccine construct with a threshold value of 0.4. Viral databases were used to extract whole-protein antigenicity prediction models. Each set was made up of 100 identified antigens, and 100 non-antigens. The generated models were evaluated using data sets, utilizing internal leave-one-out cross-validation and external validation. The models implemented in the server worked well in both validations showing 70% to 89% predictive accuracy. Also, the allergenicity of the vaccine was checked using AllerTOP server^85^. This server employs auto-cross-covariance (ACC) grouping of protein sequences into uniform equal-length vectors. This has been applied to peptide study with the various types with quantitative structure–activity relationships (QSAR). The K-nearest neighbour algorithm (kNN, k = 1) is used by the server to identify proteins based on a training set composed of 2,427 identified allergens and 2,427 non-allergens of various species. In addition, the allergenicity of the designed vaccine was cross checked by AllergenFP server (https://ddg-pharmfac.net/AllergenFP/)^86^. Other physicochemical properties like Isoelectric point, molecular weight, instability index, aliphatic index, half-life and GRAVY score of the vaccine was assessed using ExPASy ProtParam server^89^. The vaccine construct was also checked for the presence of any signal peptides and transmembrane helices by SignalP4.1 (https://www.cbs.dtu.dk/services/SignalP/)^118^ and TMHMM server v2.0 (https://www.cbs.dtu.dk/services/TMHMM/)^119^ , respectively.

### Docking with TLR4 dimer, TLR2, MHC class I receptor and MHC class II receptor

For generation of a stable immune response, it is essential for the vaccine to interact with target immune cell receptors. To study such interactions, molecular docking studies are performed. In this study, interactions of the vaccine with TLR4 dimer and TLR2 are studied as they localize on cell surface thereby inducing immune response when activated by the vaccine^120,121^. In addition, the vaccine was also docked with MHC class I and MHC class II receptors. TLR4 hetero-tetramer structure and TLR2 structure were obtained from Protein Data Bank ID 3FXI and ID 2Z7X, respectively whereas, the MHC class I and MHC class II receptors were obtained from PDB ID 1I1Y and 1KG0, respectively.

CPORT^122^ was utilized for predicting the active and passive residues for the interactions. The docking of the vaccine with TLR4, TLR2, MHC class I and MHC class II receptors were performed by HADDOCK 2.4 (https://www.bonvinlab.org/software/haddock2.4/)^123^. The best cluster was chosen from the docked clusters based on lowest HADDOCK score. HADDOCK Refinement Interface was used to refine the chosen cluster. The best structure after refinement from each docked complex were chosen and their binding affinity was calculated using PRODIGY web server^124,125^. Finally, the interacting residues between the vaccine and the TLRs were mapped using PDBsum (https://www.ebi.ac.uk/thornton-srv/databases/pdbsum/Generate.html)^126^.

### Energy minimization and molecular dynamics simulation

GROMACS (GROningen MAchine for Chemical Simulations), a Linux-based program was used for the Molecular Dynamics Simulation (MDS) and energy minimisation^127^. MDS was done for the vaccine structure in order to see how it behaves in the in vivo biological system. OPLS-AA (Optimized Potential for Liquid Simulation-All Atom) force field constrain was used to generate the topology file required for energy minimization and equilibration. An equilibrated three-point water model, spc216 was used as the solvent to simulate the vaccine with periodic boundary conditions. The net charge of the vaccine construct was evaluated, and charged ions were added in order to neutralize the system. The simulation run was performed for 10 ns of the energy minimised structure in order to find the Root Mean Square Deviation (RMSD) of backbone and Root Mean Square Fluctuation (RMSF) of side chain. The graphs were visualized using Xmgrace plotting tool^128^.

### Reverse translation, codon optimization and in silico cloning of the vaccine

The Java Codon Adaptation Tool (JCat) (https://www.jcat.de/) was used for codon optimization and reverse translation which generated the cDNA sequence of the vaccine that can be used for an efficient expression in *E. coli* K-12 strain^129^. The result consists of GC content and codon adaptation index (CAI) score, that can be used to assess protein expression levels. In addition, the optimized multi-epitope vaccine sequence was inserted into the pET-28a (+) vector by SnapGene tool.

### Immune simulation

C-IMMSIM server **(**https://kraken.iac.rm.cnr.it/C-IMMSIM/**)** was used for performing the immune simulation of the vaccine, in order to characterize the immune response profile and immunogenicity of the chimeric peptides^53^. C-IMMSIM is an agent-based model that uses position-specific scoring matrices (PSSM) for peptide prediction derived from machine learning techniques for predicting immune interactions. The minimum recommended time between dose 1 and dose 2 for most of the vaccines currently in use, is 4 weeks^130^. The entire simulation ran for 1,400 time steps which are about 15 months (a time step is about 8 h). Two peptide injections were given four weeks apart at time step 10, 94, 178, 262, 346, 430, 514, 598, 682, 766, 850, 934. Then a live virus was injected at time step 1,100, which is about 12 months after the simulation starts.

## Conclusion

The current global pandemic of COVID-19 caused by SARS-CoV-2 is to date un-controllable with high death rate. No proper medical preventives like vaccines are given to the patients yet for recovery. Application of in silico methods can be used to design an effective vaccine in lesser time and low cost. In this study, immunoinformatic tools are used for constructing a multi-epitope vaccine against SARS-CoV-2 consisting of CTL, HTL and IFN-γ epitopes that can trigger strong immune responses. The designed multi-epitope vaccine was found to be both antigenic and immunogenic. The stability of the designed vaccine was assured by molecular dynamics simulation and a stable interaction of the vaccine with immune receptors was confirmed by Molecular Docking studies. Further, in silico expression studies confirmed the vaccine’s expression in bacterial host and the efficiency of the vaccine to trigger an immune response was validated by Immune Simulation studies.

## Supplementary information

Supplementary file1
