# Supplementary material for: A candidate multi-epitope vaccine against SARS-CoV-2
Source: Sci Rep. 2020 Jul 2;10:10895. doi: 10.1038/s41598-020-67749-1 (PMC7331818; doi:10.1038/s41598-020-67749-1)
Supplement: Supplementary file 1 — Supplementary file1 [file 41598_2020_67749_MOESM1_ESM.pdf]

A candidate multi-epitope vaccine against SARS-CoV-2

Tamalika Kar[1*], Utkarsh Narsaria[1*], Srijita Basak[1*], Debashrito Deb[1*], Filippo Castiglione[2], David M. Mueller[3], Anurag P. Srivastava[1†]

[1]Department of Life Sciences

Garden City University

Bangalore, Karnataka,

India

[2]Institute for Applied Computing

National Research Council of Italy

Via dei Taurini, Rome,

Italy

[3]Center for Genetic Diseases

The Chicago Medical School

Rosalind Franklin University of Medicine and Science

North Chicago,

USA

*These authors contributed equally to this work.

†Corresponding Author

Anurag P. Srivastava: anurag.srivastava@gardencity.university, anuiitkgp@gmail.com

Phone: +91-9108720555

ORCID ID: 0000-0003-3493-1375

SUPPLEMENTARY TABLES

Supplementary Table 1:

All the CTL epitopes as predicted by NetCTL 1.2

CTL Epitope prediction by NetCTL-1.2 Server					
**MHC Super type**	**EPITOPES**	**POSITION**	**PREDICTION SCORE**	**IMMUNOGENIC SCORE**	**ANTIGENICITY SCORE**
MHC supertype A1. Threshold 0.750000	LTDEMIAQY	865	3.6616	0.02757	0.1043
	WTAGAAAYY	258	3.1128	0.15259	0.6306
	TSNQVAVLY	604	3.0758	-0.01327	0.4387
	CVADYSVLY	361	2.5759	-0.09595	-0.0293
	KTSVDCTMY	733	2.3795	-0.11115	1.1824
	STECSNLLL	746	2.3492	-0.20478	0.4871
	GAEHVNNSY	652	1.9960	-0.00296	0.9347
	NIDGYFKIY	196	1.9606	0.04366	-0.2462
	YSSANNCTF	160	1.9531	-0.04954	-0.1036
	WMESEFRVY	152	1.9232	0.14153	0.2698
	SANNCTFEY	162	1.8739	0.13273	-0.0924
	VASQSIIAY	687	1.7978	-0.0709	0.1366
	NSFTRGVYY	30	1.6915	0.19204	-0.1877
	CNDPFLGVY	136	1.3355	0.15232	0.4295
	FTNVYADSF	392	1.3208	-0.00527	-0.5651
	GAAAYYVGY	261	1.2194	0.09963	0.6604
	RISNCVADY	357	1.2032	-0.02787	-0.5375
	ERDISTEIY	465	1.1687	0.17882	-0.2317
	ITDAVDCAL	285	1.1680	0.08501	0.5260
	RVDFCGKGY	1039	1.1403	-0.0578	0.3717
	NATRFASVY	343	1.1138	0.10001	-0.0081
	STQDLFLPF	50	1.0468	0.06828	0.6619
	VSNGTHWFV	1096	0.9574	0.35559	-0.1239
	GTITSGWTF	880	0.9433	0.16268	0.3272
	RSFIEDLLF	815	0.8441	0.27446	-0.5782
	HSAWSHPQF	1257	0.8279	0.0279	0.8569
	ECSNLLLQY	748	0.8171	-0.15849	0.3331
	NSASFSTFK	370	0.8165	-0.09434	0.1232
	ASFSTFKCY	372	0.8085	-0.19397	0.2795
	QLTPTWRVY	628	0.7887	0.31555	1.2119
	LSETKCTLK	296	0.7879	-0.16291	0.6883
	FVFKNIDGY	192	0.7837	-0.0215	-0.1304
	VGGNYNYLY	445	0.7698	-0.0148	0.7432
	VLPFNDGVY	83	0.7675	0.1815	0.4642
	FVSNGTHWF	1095	0.7622	0.16605	0.0807
	YQDVNCTEV	612	0.7502	0.08295	1.6172
MHC supertype A2. Threshold 0.750000	YLQPRTFLL	269	1.5152	0.1305	0.4532
	KIADYNYKL	417	1.4347	-0.10379	1.6639
	SIIAYTMSL	691	1.3658	-0.12935	0.5234
	VLNDILSRL	976	1.3540	0.03	-0.8524
	RLQSLQTYV	1000	1.2727	-0.29331	-0.2167
	FVFLVLLPL	2	1.1947	0.04076	0.8601
	FTISVTTEI	718	1.1808	0.04473	0.8535
	MIAQYTSAL	869	1.1735	-0.18768	0.1114
	FQFCNDPFL	133	1.1416	0.05737	-0.2493
	FLGRSLEVL	1236	1.1019	0.00016	0.6113
	TLDSKTQSL	109	1.0777	-0.52715	1.0685

	SVTTEILPV	721	1.0675	0.2586	0.8441
	VVFLHVTYV	1060	1.0304	0.1278	1.5122
	FLHVTYVPA	1062	1.0258	0.11472	1.3346
	LLFNKVTLA	821	1.0091	-0.11337	0.6150
	NLNESLIDL	1192	0.9825	0.05239	0.6827
	HLMSFPQSA	1048	0.9793	-0.31433	-0.0681
	YQDVNCTEV	612	0.9571	0.08295	1.6172
	ITSGWTFGA	882	0.9541	0.35124	0.4577
	IITTDNTFV	1114	0.9539	0.16833	0.4733
	RLDPPEAEV	983	0.9436	0.17101	0.4496
	KIYSKHTPI	202	0.9329	-0.32094	0.7455
	QLNRALTGI	762	0.9308	0.1302	0.0080
	GLTVLPPLL	857	0.9214	0.01706	0.6621
	VTWFHAIHV	62	0.9144	0.38925	0.5426
	VLYENQKLI	915	0.9028	-0.20427	0.4361
	KQIYKTPPI	786	0.8755	-0.14982	0.2705
	KQLSSNFGA	964	0.8743	-0.21866	0.3753
	KMSECVLGQ	1028	0.8514	0.04385	-0.1017
	RVVVLSFEL	509	0.8445	0.04571	1.1918
	YTNSFTRGV	28	0.8445	0.04545	-0.6177
	VLSFELLHA	512	0.8218	0.1607	1.0776
	YQPYRVVVL	505	0.8143	0.1409	0.5964
	AVDCALDPL	288	0.8102	-0.00715	0.6604
	NVYADSFVI	394	0.7994	0.02696	-0.5617
	SLSSTASAL	937	0.7991	-0.2623	0.7716
	LLALHRSYL	241	0.7663	-0.06002	0.5241
	TSVDCTMYI	734	0.7648	-0.1306	0.7328
	ALNTLVKQL	958	0.7518	-0.18466	-0.5716
MHC supertype A3. Threshold 0.750000	RLFRKSNLK	454	1.7563	-0.28759	-0.2829
	GVYFASTEK	89	1.4615	0.09023	0.7112
	QIYKTPPIK	787	1.4526	-0.12244	-0.0833
	VTYVPAQEK	1065	1.3960	0.02711	0.8132
	TLKSFTVEK	302	1.3483	0.00741	0.0809
	NSASFSTFK	370	1.3454	-0.09434	0.1232
	KVFRSSVLH	41	1.3419	-0.19839	-0.6913
	GVYYHKNNK	142	1.3335	-0.18566	0.8264
	KCYGVSPTK	378	1.2722	-0.06931	1.4199
	VLLSTFLGR	1231	1.2481	-0.01163	0.1933
	TLADAGFIK	827	1.2451	0.28158	0.5781
	EILPVSMTK	725	1.2271	-0.25581	1.6842
	ASANLAATK	1020	1.2193	0.08792	0.7014
	TSNQVAVLY	604	1.1959	-0.01327	0.4387
	RISNCVADY	357	1.1734	-0.02787	-0.5375
	HVSGTNGTK	69	1.1575	0.06339	1.0956
	SVYAWNRKR	349	1.1423	0.16546	0.7650
	CVADYSVLY	361	1.1344	-0.09595	-0.0293
	GIYQTSNFR	311	1.1119	-0.17275	0.5380
	NQFNSAIGK	925	1.0796	0.03934	-0.1202
	GSFCTQLNR	757	1.0783	-0.10063	0.9306
	SSTASALGK	939	1.0678	-0.06186	0.6215
	TLLALHRSY	240	1.0560	0.00244	0.8009
	LQPRTFLLK	270	1.0511	0.18064	0.0371
	QIAPGQTGK	409	1.0483	-0.02194	1.8297
	GTHWFVTQR	1099	1.0342	0.35133	0.0723
	AQALNTLVK	956	1.0252	0.04654	-0.2279
	NSFTRGVYY	30	0.9914	0.19204	-0.1877

	GVLTESNKK	550	0.9827	-0.15423	0.8797
	KSTNLVKNK	529	0.9809	-0.15163	0.8596
	KSNLKPFER	458	0.9792	-0.0764	0.9490
	SVLNDILSR	975	0.9477	0.03075	-0.7223
	ASFSTFKCY	372	0.8899	-0.19397	0.2795
	KTSVDCTMY	733	0.8544	-0.11115	1.1824
	SANNCTFEY	162	0.8377	0.13273	-0.0924
	LSETKCTLK	296	0.8213	-0.16291	0.6883
	FLPFQQFGR	559	0.8186	0.01096	0.4802
	FIEDLLFNK	817	0.8027	0.1286	-0.2768
	RVDFCGKGY	1039	0.7565	-0.0578	0.3717
	KNLREFVFK	187	0.7504	0.35942	-0.5146
MHC supertype A24. Threshold 0.750000	NYNYLYRLF	448	1.9482	0.0171	-0.1480
	PYRVVVLSF	507	1.8786	0.03138	1.0281
	VYSTGSNVF	635	1.8571	-0.11871	-0.3099
	EYVSQPFLM	169	1.7025	-0.18399	0.2605
	YFPLQSYGF	489	1.6931	-0.26661	0.5107
	IYQTSNFRV	312	1.6294	-0.03669	0.3109
	TFEYVSQPF	167	1.6168	-0.19099	0.6641
	VYAWNRKRI	350	1.5755	0.12625	0.5003
	AYSNNSIAI	706	1.5591	-0.08706	0.8274
	PFFSNVTWF	57	1.4947	0.06627	0.6638
	EWVLLSTFL	1229	1.3414	-0.06313	0.4215
	NYLYRLFRK	450	1.3274	0.16168	-0.8611
	CYGVSPTKL	379	1.2262	-0.21224	1.4263
	YYHKNNKSW	144	1.2214	-0.49755	0.4536
	VYDPLQPEL	1137	1.1735	-0.07466	0.4525
	VFKNIDGYF	193	1.0997	0.10041	-0.7572
	IAIPTNFTI	712	1.0788	0.18523	0.7052
	GTITSGWTF	880	1.0715	0.16268	0.3272
	SVLYNSASF	366	1.0673	-0.23299	0.1857
	RFDNPVLPF	78	1.0633	0.01291	0.1828
	EFQFCNDPF	132	1.0541	0.03385	0.0062
	SWMESEFRV	151	1.0450	0.10594	0.6510
	QYGSFCTQL	755	1.0275	-0.12714	1.2906
	VFLVLLPLV	3	0.9838	0.00086	0.4651
	HWFVTQRNF	1101	0.9755	0.0482	0.7460
	KYEQGSGYI	1205	0.9501	-0.18035	0.1781
	QYGDCLGDI	836	0.9087	0.01194	0.0410
	VFVSNGTHW	1094	0.9023	-0.07581	0.3438
	YQPYRVVVL	505	0.9003	0.1409	0.5964
	VFQTRAGCL	642	0.8790	0.08579	1.7094
	VYADSFVIR	395	0.8285	0.09672	-0.2347
	YYPDKVFRS	37	0.8245	-0.02338	-0.3237
	YSSANNCTF	160	0.8021	-0.04954	-0.1036
	QIITTDNTF	1113	0.7939	0.15816	0.4253
	HSAWSHPQF	1257	0.7847	0.0279	0.8569
	STQDLFLPF	50	0.7748	0.06828	0.6619
	IAQYTSALL	870	0.7565	-0.13271	0.0823
MHC supertype A26. Threshold 0.750000	FVFKNIDGY	192	2.2795	-0.0215	-0.1304
	WTAGAAAYY	258	2.0048	0.15259	0.6306
	EAPRDGQAY	1215	1.5430	0.02708	-0.6230
	ECSNLLLQY	748	1.5378	-0.15849	0.3331
	ETKCTLKSF	298	1.5231	-0.37555	0.8720
	CVADYSVLY	361	1.5087	-0.09595	-0.0293
	NGVEGFNCY	481	1.4016	0.22039	0.7783

	STQDLFLPF	50	1.2106	0.06828	0.6619
	SIIAYTMSL	691	1.1486	-0.12935	0.5234
	FVSNGTHWF	1095	1.1411	0.16605	0.0807
	QIITTDNTF	1113	1.1343	0.15816	0.4253
	STASALGKL	940	1.1108	-0.22351	0.5357
	SFKEELDKY	1147	1.0689	0.01053	-1.3338
	LTDEMIAQY	865	1.0671	0.02757	0.1043
	ESNKKFLPF	554	1.0271	-0.33474	1.0278
	DIADTTDAV	568	1.0123	0.15094	1.0904
	FVFLVLLPL	2	1.0076	0.04076	0.8601
	FTISVTTEI	718	1.0014	0.04473	0.8535
	SVLYNSASF	366	1.0009	-0.23299	0.1857
	EVAKNLNES	1188	0.9762	-0.168	0.0468
	FQPTNGVGY	497	0.9325	0.1157	0.3114
	WTFGAGAAL	886	0.9302	0.19798	0.4918
	NSFTRGVYY	30	0.9271	0.19204	-0.1877
	GTITSGWTF	880	0.8856	0.16268	0.3272
	ASFSTFKCY	372	0.8790	-0.19397	0.2795
	DLPQGFSAL	215	0.8620	-0.09372	0.5622
	TSNQVAVLY	604	0.8615	-0.01327	0.4387
	EVFAQVKQI	780	0.8432	-0.24625	0.2447
	NTSNQVAVL	603	0.7916	-0.07701	0.4987
	EVFNATRFA	340	0.7775	0.21821	0.2087
	YVPAQEKNF	1067	0.7664	-0.16856	0.8731
MHC supertype B7. Threshold 0.750000	IPTNFTISV	714	1.5427	0.17229	0.8820
	LPPAYTNSF	24	1.5189	-0.0334	0.3775
	APRDGQAYV	1216	1.4513	-0.00606	-0.4030
	MIAQYTSAL	869	1.2704	-0.18768	0.1114
	GPKKSTNLV	526	1.2245	-0.4235	0.6828
	SPGSASSVA	680	1.1757	-0.3886	0.4280
	SVAYSNNSI	704	1.1296	-0.26033	0.8783
	VVNQNAQAL	951	1.1258	-0.16303	0.4749
	TPTWRVYST	630	1.0987	0.22497	0.4605
	RAAEIRASA	1014	1.0923	0.22813	0.2752
	LPFNDGVYF	84	1.0427	0.11767	0.5593
	QPTESIVRF	321	0.9845	0.14261	-0.5551
	APGQTGKIA	411	0.9845	-0.1401	1.2002
	SVLYNSASF	366	0.9707	-0.23299	0.1857
	IAQYTSALL	870	0.9549	-0.13271	0.0823
	TPINLVRDL	208	0.9266	0.12139	0.3862
	AALQIPFAM	892	0.9235	0.12066	0.7747
	SLSSTASAL	937	0.9090	-0.2623	0.7716
	FPQSAPHGV	1052	0.8956	-0.12931	-0.0058
	KPFERDIST	462	0.8646	0.22569	-0.4114
	IANQFNSAI	923	0.8469	-0.12751	-0.0100
	LPFFSNVTW	56	0.8467	0.04613	1.0808
	AAAYYVGYL	262	0.8439	0.07068	0.4605
	RASANLAAT	1019	0.8310	0.02481	0.7524
	LPPLLTDEM	861	0.8274	0.0882	0.4723
	RVVVLSFEL	509	0.8161	0.04571	1.1918
	RVYSTGSNV	634	0.8131	-0.2413	0.2636
	TPCNGVEGF	478	0.8026	0.15215	-0.3772
	TPGDSSSGW	250	0.7709	-0.40333	0.1330
	KPSKRSFIE	811	0.7689	-0.1994	0.2768
MHC supertype B8. Threshold 0.750000	YLQPRTFLL	269	1.9947	0.1305	0.4532
	LLALHRSYL	241	1.4391	-0.06002	0.5241

	FRKSNLKPF	456	1.3950	-0.44169	0.6280
	FLHVTYVPA	1062	1.3772	0.11472	1.3346
	MIAQYTSAL	869	1.3248	-0.18768	0.1114
	FLGRSLEVL	1236	1.2736	0.00016	0.6113
	YQPYRVVVL	505	1.1348	0.1409	0.5964
	KIYSKHTPI	202	1.1240	-0.32094	0.7455
	FKNLREFVF	186	1.1060	0.25431	0.0523
	VAKNLNESL	1189	1.0615	-0.10556	0.1790
	VIKVCEFQF	127	0.9429	0.04441	-0.1189
	INITRFQTL	233	0.8988	0.16778	0.3934
	YRLFRKSNL	453	0.8782	-0.1818	0.0522
	FNATRFASV	342	0.8751	0.14872	0.5609
	FCTQLNRAL	759	0.8545	-0.05431	0.5159
	FVFLVLLPL	2	0.8252	0.04076	0.8601
	YLYRLFRKS	451	0.7972	0.06796	-0.6217
	NLLLQYGSF	751	0.7682	-0.1991	0.6553
	LITGRLQSL	996	0.7675	-0.10776	0.8238
	LGQSKRVDF	1034	0.7535	-0.31755	2.0590
MHC supertype B27. Threshold 0.750000	GRLQSLQTY	999	1.7162	-0.36678	-0.0743
	YRLFRKSNL	453	1.6065	-0.1818	0.0522
	TRFQTLLAL	236	1.4733	-0.0377	0.3406
	FRKSNLKPF	456	1.3086	-0.44169	0.6280
	TRFASVYAW	345	1.1380	-0.02513	-0.1315
	GQTGKIADY	413	1.0811	0.00796	1.4019
	TRTQLPPAY	20	1.0554	-0.1117	1.2923
	NQFNSAIGK	925	1.0381	0.03934	-0.1202
	VRFPNITNL	327	1.0199	0.1748	1.1141
	ARDLICAQK	846	0.9787	0.04023	0.8157
	KRFDNPVLP	77	0.9728	0.07194	0.2834
	GKYEQGSGY	1204	0.9663	-0.10117	0.1691
	KRSFIEDLL	814	0.9131	0.30019	-0.6422
	FRVQPTESI	318	0.8556	-0.08958	0.9396
	YRFNGIGVT	904	0.8430	0.24249	1.7692
	AQALNTLVK	956	0.8298	0.04654	-0.2279
	SKVGGNYNY	443	0.7843	0.06751	0.9111
	KKFLPFQQF	557	0.7794	-0.0392	0.1647
	QRNFYEPQI	1106	0.7763	0.12931	0.3296
	NRALTGIAV	764	0.7752	0.20642	0.5302
MHC supertype B39. Threshold 0.750000	YQPYRVVVL	505	1.9051	0.1409	0.5964
	TRFQTLLAL	236	1.6214	-0.0377	0.3406
	HHHHHHSAW	1252	1.3068	-0.01176	0.6641
	FEYVSQPFL	168	1.2829	-0.17076	0.6324
	YRLFRKSNL	453	1.2575	-0.1818	0.0522
	VRKDGEWVL	1224	1.2148	0.29063	0.7021
	RKDGEWVLL	1225	1.1905	0.37567	1.2792
	YSSANNCTF	160	1.1779	-0.04954	-0.1036
	FLGRSLEVL	1236	1.1600	0.00016	0.6113
	YLQPRTFLL	269	1.1210	0.1305	0.4532
	QKFNGLTVL	853	1.1098	0.11093	0.2349
	NTSNQVAVL	603	1.1045	-0.07701	0.4987
	YNSASFSTF	369	1.0612	-0.18217	0.3005
	QNAQALNTL	954	1.0313	-0.05898	0.3288
	VRFPNITNL	327	1.0215	0.1748	1.1141
	YSKHTPINL	204	1.0200	0.09845	1.0547
	NHTSPDVDL	1158	1.0185	-0.09599	0.8446
	MIAQYTSAL	869	1.0023	-0.18768	0.1114

	STECSNLLL	746	0.9683	-0.20478	0.4871
	HHSAWSHPQ	1256	0.9525	0.06646	0.3725
	TISVTTEIL	719	0.9188	0.22444	0.4795
	KIADYNYKL	417	0.9011	-0.10379	1.6639
	YHKNNKSWM	145	0.8840	-0.29601	0.3388
	YQDVNCTEV	612	0.8753	0.08295	1.6172
	FQFCNDPFL	133	0.8735	0.05737	-0.2493
	LHSTQDLFL	48	0.8493	-0.04752	0.3504
	ITDAVDCAL	285	0.8468	0.08501	0.5260
	TLDSKTQSL	109	0.8448	-0.52715	1.0685
	HHHHHHHSA	1251	0.8195	0.03564	0.6099
	HSAWSHPQF	1257	0.8099	0.0279	0.8569
	VYDPLQPEL	1137	0.8051	-0.07466	0.4525
	TRFASVYAW	345	0.7632	-0.02513	-0.1315
	TKRFDNPVL	76	0.7519	0.16487	0.1808
MHC supertype B44. Threshold 0.750000	FEYVSQPFL	168	1.8668	-0.17076	0.6324
	AEIRASANL	1016	1.8005	0.00689	0.7082
	GEVFNATRF	339	1.7314	0.22473	-0.1511
	FERDISTEI	464	1.4106	0.10425	-0.7442
	GEWVLLSTF	1228	1.3472	-0.02474	0.4373
	AEVQIDRLI	989	1.2946	0.08452	-0.5562
	IEDLLFNKV	818	1.1386	-0.03602	0.2737
	LEPLVDLPI	223	1.1040	0.03048	-0.0069
	KEIDRLNEV	1206	0.9854	0.15852	0.5300
	YEQGSGYIP	1181	0.9562	-0.05806	0.0027
	SEFRVYSSA	155	0.8732	-0.10948	0.0315
	YECDIPIGA	660	0.8413	0.2561	0.6385
	FQFCNDPFL	133	0.8223	0.05737	-0.2493
MHC supertype B58. Threshold 0.750000	RSFIEDLLF	815	1.9914	0.27446	-0.5782
	HSAWSHPQF	160	1.9696	0.0279	0.8569
	GSAWSHPQF	1257	1.8837	0.0279	0.4085
	YSSANNCTF	1278	1.7958	-0.04954	-0.1036
	HADQLTPTW	625	1.7854	-0.0703	0.3807
	NSIAIPTNF	710	1.6445	0.23071	0.1744
	QSAPHGVVF	1054	1.6400	0.1239	0.2234
	IAIPTNFTI	712	1.5865	0.18523	0.7052
	FAMQMAYRF	898	1.5848	-0.28061	1.0278
	GTITSGWTF	880	1.5724	0.16268	0.3272
	TSNQVAVLY	604	1.4503	-0.01327	0.4387
	KTSVDCTMY	733	1.4355	-0.11115	1.1824
	FTNVYADSF	392	1.4262	-0.00527	-0.5651
	LAGTITSGW	878	1.4057	0.09638	0.3218
	SANNCTFEY	162	1.3729	0.13273	-0.0924
	VFVSNGTHW	1094	1.2876	-0.07581	0.3438
	WTAGAAAYY	258	1.2345	0.15259	0.6306
	RVVVLSFEL	509	1.1931	0.04571	1.1918
	IGIVNNTVY	1130	1.1809	0.12923	0.9758
	VASQSIIAY	687	1.1427	-0.0709	0.1366
	ILDITPCSF	584	1.1291	0.02632	1.1835
	KGIYQTSNF	310	1.0598	-0.18018	0.4627
	ITDAVDCAL	285	0.9517	0.08501	0.5260
	LTDEMIAQY	865	0.9287	0.02757	0.1043
	FTISVTTEI	718	0.9262	0.04473	0.8535
	GAAAYYVGY	261	0.9052	0.09963	0.6604
	LPFFSNVTW	56	0.8818	0.04613	1.0808
	KVTLADAGF	825	0.8650	0.11324	1.3717

	ISSVLNDIL	973	0.8197	0.06743	-0.3334
	QIITTDNTF	1113	0.8053	0.15816	0.4253
	AALQIPFAM	892	0.8018	0.12066	0.7747
	VSNGTHWFV	1096	0.7630	0.35559	-0.1239
	IGAGICASY	666	0.7545	0.06201	0.6368
MHC supertype B62. Threshold 0.750000	TLLALHRSY	240	1.3654	0.00244	0.8009
	YSSANNCTF	160	1.3628	-0.04954	0.1366
	WTAGAAAYY	258	1.3574	0.15259	0.6306
	VASQSIIAY	687	1.3405	-0.0709	0.1366
	QLTPTWRVY	628	1.3281	0.31555	1.2119
	GQTGKIADY	413	1.3104	0.00796	1.4019
	GVYYPDKVF	35	1.2993	-0.15272	0.0652
	FQPTNGVGY	497	1.2969	0.1157	0.3114
	FVSNGTHWF	1095	1.2938	0.16605	0.0807
	LGAENSVAY	699	1.2832	0.00912	0.4173
	QSAPHGVVF	366	1.2612	0.1239	0.2234
	SVLYNSASF	1054	1.2472	-0.23299	0.1857
	WMESEFRVY	152	1.2452	0.14153	0.2698
	YNSASFSTF	369	1.2447	-0.18217	0.3005
	NQKLIANQF	919	1.2266	0.01213	-0.4784
	LQIPFAMQM	894	1.2266	-0.03301	1.0680
	IGAGICASY	666	1.2205	0.06201	0.6368
	QIITTDNTF	1113	1.2195	0.15816	0.4253
	STQDLFLPF	50	1.1921	0.06828	0.6619
	IGIVNNTVY	1130	1.1713	0.12923	0.9758
	YQPYRVVVL	505	1.1670	0.1409	0.5964
	RISNCVADY	357	1.1648	-0.02787	-0.5375
	LVRDLPQGF	212	1.1640	-0.06008	0.1234
	RSFIEDLLF	815	1.1579	0.27446	-0.5782
	ASFSTFKCY	372	1.1576	-0.19397	0.2795
	LVKQLSSNF	962	1.1546	-0.49649	-0.3212
	WTFGAGAAL	886	1.1379	0.19798	0.4918
	NSFTRGVYY	30	1.1316	0.19204	-0.1877
	SANNCTFEY	162	1.1257	0.13273	-0.0924
	GVVFLHVTY	1059	1.1240	0.20837	1.4104
	GAAAYYVGY	261	1.1104	0.09963	0.6604
	KTSVDCTMY	733	1.1039	-0.11115	1.1824
	HSAWSHPQF	1257	1.1001	0.0279	0.8569
	FVFKNIDGY	192	1.0936	-0.0215	1.3263
	LVKNKCVNF	533	1.0866	-0.3062	1.3263
	TSNQVAVLY	604	1.0862	-0.01327	0.4387
	VLTESNKKF	551	1.0563	-0.36184	0.5392
	GTITSGWTF	880	1.0504	0.16268	0.3272
	SQSIIAYTM	689	1.0453	0.26621	0.2614
	KQIYKTPPI	786	1.0425	-0.14982	0.2705
	AGAALQIPF	890	1.0325	0.03807	0.4855
	VLPFNDGVY	83	0.9990	0.1815	0.4642
	GSAWSHPQF	1278	0.9943	0.0279	0.4085
	NLCPFGEVF	334	0.9922	0.22586	0.1999
	FVFLVLLPL	2	0.9849	0.04076	0.8601
	AQKFNGLTV	852	0.9799	0.08672	0.1405
	CVADYSVLY	361	0.9750	-0.09595	-0.0293
	FQGPGHHHH	1245	0.9725	0.10949	-0.2291
	FTNVYADSF	392	0.9602	-0.00527	-0.5651

Supplementary Table 2:

All the HTL epitopes as predicted by NetMHC II pan 3.2

EPITOPES (POSITION)	CORE SEQUENCE	HLA ALLELES II	SCORE	IMMUNOGE NICITY	ANTIGENICIT Y
VLSFELLHAPATVCG (512)	FELLHAPAT	DRB1*01:01	0.30	0.27678	0.4784
IIAYTMSLGAENSVA (692)	YTMSLGAEN	DRB1*01:01	0.40	-0.24911	0.5426
		DRB1*04:01	0.60		
		DRB1*04:05	1.00		
		DRB1*07:01	2.00		
		DRB1*09:01	0.20		
		DQA1*04:01-DQB1*04:02	1.70		
		DQA1*01:02-DQB1*06:02	2.00		
		DPA1*02:01-DPB1*14:01	1.90		
VVLSFELLHAPATVC (511)	FELLHAPAT	DRB1*01:01	0.40	0.17234	0.8618
VVVLSFELLHAPATV (510)	FELLHAPAT	DRB1*01:01	0.40	0.1459	0.8083
LSFELLHAPATVCGP (513)	FELLHAPAT	DRB1*01:01	0.40	0.26501	0.5062
SIIAYTMSLGAENSV (691)	YTMSLGAEN	DRB1*01:01	0.50	-0.1629	0.5691
		DRB1*04:01	0.70		
		DRB1*04:05	1.00		
		DRB1*07:01	1.60		
		DRB1*09:01	0.20		
		DQA1*04:01-DQB1*04:02	1.80		
		DPA1*02:01-DPB1*14:01	1.90		
TRFQTLLALHRSYLT (236)	FQTLLALHR	DRB1*01:01	0.70	-0.11849	0.3262
		DRB1*04:01	1.50		
		DRB1*04:05	0.60		
		DRB1*08:02	1.60		
		DRB1*11:01	0.30		
		DRB1*12:01	0.40		
		DRB1*15:01	0.30		
		DRB4*01:01	1.20		
		DPA1*02:01-DPB1*05:01	0.60		
		DPA1*02:01-DPB1*14:01	0.90		
		DRB5*01:01	0.09		
ITRFQTLLALHRSYL (235)	FQTLLALHR	DRB1*01:01	0.70	0.00374	0.1131
		DRB1*04:01	1.10		
		DRB1*04:05	0.40		
		DRB1*08:02	1.40		
		DRB1*11:01	0.40		
		DRB1*12:01	0.30		
		DRB1*15:01	0.50		
		DRB4*01:01	0.70		
		DPA1*02:01-DPB1*05:01	0.40		
		DPA1*02:01-DPB1*14:01	0.50		
		DRB5*01:01	0.08		
IAYTMSLGAENSVAY (693)	YTMSLGAEN	DRB1*01:01	0.70	-0.24077	0.7072
		DRB1*04:01	1.70		
		DQA1*04:01-DQB1*04:02	2.00		
		DRB1*09:01	0.40		
NITRFQTLLALHRSY (234)	FQTLLALHR	DRB1*01:01	0.70	0.08685	0.1775
		DRB1*04:01	0.70		
		DRB1*04:05	0.20		

		DRB1*08:02	1.20		
		DRB1*11:01	0.50		
		DRB1*12:01	0.70		
		DRB1*15:01	0.40		
		DRB4*01:01	0.50		
		DPA1*02:01-DPB1*05:01	0.40		
		DPA1*02:01-DPB1*14:01	0.50		
		DRB5*01:01	0.09		
RFQTLLALHRSYLTP (237)	FQTLLALHR	DRB1*01:01	0.70	-0.0622	0.5470
		DRB1*04:01	1.20		
		DRB1*08:02	1.90		
		DRB1*11:01	0.50		
		DRB1*12:01	0.50		
		DRB1*15:01	0.25		
		DRB4*01:01	1.40		
		DPA1*02:01-DPB1*05:01	1.00		
		DPA1*02:01-DPB1*14:01	2.00		
		DRB5*01:01	0.17		
QKFNGLTVLPPLLTD (853)	FNGLTVLPP	DRB1*01:01	1.00	0.10217	0.2436
		DRB1*04:01	1.20		
		DRB1*04:05	0.50		
		DPA1*02:01-DPB1*14:01	1.90		
QSIIAYTMSLGAENS (690)	YTMSLGAEN	DRB1*01:01	1.00	0.05707	0.5728
		DRB1*04:01	1.00		
		DRB1*04:05	1.40		
		DRB1*07:01	2.00		
		DRB1*09:01	0.40		
INITRFQTLLALHRS (233)	FQTLLALHR	DRB1*01:01	1.00	0.23526	0.4118
		DRB1*04:01	0.80		
		DRB1*04:05	0.25		
		DRB1*08:02	1.60		
		DRB1*11:01	0.60		
		DRB1*12:01	0.90		
		DRB1*15:01	0.30		
		DRB4*01:01	0.50		
		DPA1*02:01-DPB1*05:01	0.40		
		DPA1*02:01-DPB1*14:01	0.70		
		DRB5*01:01	0.12		
QIPFAMQMAYRFNGI (895)	FAMQMAYRF	DRB1*01:01	1.00	-0.09306	0.9573
		DRB1*12:01	1.20		
		DRB1*15:01	1.50		
		DRB4*01:01	1.20		
		DRB5*01:01	0.70		
SFELLHAPATVCGPK (514)	FELLHAPAT	DRB1*01:01	1.00	0.15681	0.2085
LQIPFAMQMAYRFNG (894)	FAMQMAYRF	DRB1*01:01	1.10	-0.09242	0.7205
		DRB1*09:01	2.00		
		DRB1*12:01	1.40		
		DRB1*15:01	1.50		
		DRB4*01:01	1.10		
		DRB5*01:01	0.70		
AQKFNGLTVLPPLLT (852)	FNGLTVLPP	DRB1*01:01	1.10	0.09382	0.2860
		DRB1*04:01	0.90		
		DRB1*04:05	0.30		
		DPA1*02:01-DPB1*14:01	1.60		
ALQIPFAMQMAYRFN (893)	FAMQMAYRF	DRB1*01:01	1.20	-0.07048	1.0112
		DRB1*09:01	1.60		

		DRB1*12:01	1.10		
		DRB1*15:01	1.40		
		DRB4*01:01	1.30		
		DRB5*01:01	0.90		
VNFNFNGLTGTGVLT (539)	FNGLTGTGV	DRB1*01:01	1.60	0.33025	1.2439
		DRB1*09:01	1.20		
IPFAMQMAYRFNGIG (896)	FAMQMAYRF	DRB1*01:01	1.60	-0.07826	1.2828
		DRB4*01:01	1.80		
		DRB5*01:01	1.10		
GINITRFQTLLALHR (232)	FQTLLALHR	DRB1*01:01	1.60	0.28379	0.5582
	ITRFQTLLA	DRB1*04:01	1.00		
		DRB1*04:05	0.25		
		DRB1*11:01	1.30		
		DRB1*12:01	0.80		
		DRB1*15:01	0.25		
	FQTLLALHR	DRB4*01:01	0.50		
		DRB5*01:01	0.30		
		DPA1*03:01-DPB1*04:02	2.00		
		DPA1*02:01-DPB1*05:01	0.50		
		DPA1*02:01-DPB1*14:01	1.00		
		DPA1*02:01-DPB1*01:01	1.60		
KFNGLTVLPPLLTDE (854)	LTVLPPLLT	DRB1*01:01	1.60	0.09128	0.3296
	FNGLTVLPP	DRB1*04:05	1.30		
NFNFNGLTGTGVLTE (540)	FNGLTGTGV	DRB1*01:01	1.70	0.32538	1.0516
		DRB1*09:01	1.40		
GWTFGAGAALQIPFA (885)	FGAGAALQI	DRB1*01:01	2.00	0.37142	0.4665
		DRB1*09:01	0.20		
		DQA1*03:01-DQB1*03:02	0.60		
		DQA1*04:01-DQB1*04:02	0.40		
		DQA1*01:02-DQB1*06:02	0.60		
		DQA1*05:01-DQB1*03:01	0.10		
SFVIRGDEVRQIAPG (399)	IRGDEVRQI	DRB1*03:01	0.50	0.47944	0.5882
		DRB3*01:01	1.70		
DSFVIRGDEVRQIAP (398)	IRGDEVRQI	DRB1*03:01	0.50	0.52838	0.1792
		DRB3*01:01	1.50		
ADSFVIRGDEVRQIA (397)	IRGDEVRQI	DRB1*03:01	0.50	0.50532	-0.0663
		DRB3*01:01	1.40		
PINLVRDLPQGFSAL (209)	LVRDLPQGF	DRB1*03:01	0.50	-0.00902	0.6086
		DRB3*01:01	1.00		
INLVRDLPQGFSALE (210)	LVRDLPQGF	DRB1*03:01	0.60	-0.00665	0.5842
		DRB3*01:01	1.20		
TPINLVRDLPQGFSA (208)	LVRDLPQGF	DRB1*03:01	0.60	0.03773	0.5531
		DRB3*01:01	1.20		
FVIRGDEVRQIAPGQ (400)	IRGDEVRQI	DRB1*03:01	0.70	0.40205	0.4940
HTPINLVRDLPQGFS (207)	LVRDLPQGF	DRB1*03:01	1.10	0.19178	0.3970
		DRB3*01:01	1.60		
NLVRDLPQGFSALEP (211)	LVRDLPQGF	DRB1*03:01	1.40	0.02455	0.4354
YADSFVIRGDEVRQI (396)	IRGDEVRQI	DRB1*03:01	1.60	0.34189	-0.0699
TSNFRVQPTESIVRF (315)	FRVQPTESI	DRB1*04:01	0.60	0.24726	0.4574
		DRB1*04:05	1.60		
AAEIRASANLAATKM (1015)	IRASANLAA	DRB1*04:01	0.70	0.09651	0.7125
		DRB1*08:02	0.70		
		DRB1*13:01	1.10		
		DPA1*02:01-DPB1*14:01	0.50		
		DQA1*01:02-DQB1*06:02	1.30		
		DRB3*02:02	1.10		
RAAEIRASANLAATK (1014)	IRASANLAA	DRB1*04:01	0.70	0.28398	0.5709
		DRB1*08:02	0.80		
		DRB1*13:01	1.30		

		DPA1*02:01-DPB1*14:01	0.70		
		DQA1*01:02-DQB1*06:02	1.70		
		DRB3*02:02	1.20		
QTSNFRVQPTESIVR (314)	FRVQPTESI	DRB1*04:01	0.80	0.1368	0.4885
SNFRVQPTESIVRFP (316)	FRVQPTESI	DRB1*04:01	0.90	0.26142	0.2635
AEIRASANLAATKMS (1016)	IRASANLAA	DRB1*04:01	1.00	-0.17858	0.8255
		DRB1*08:02	1.10		
		DRB1*13:01	1.50		
		DRB3*02:02	1.50		
		DQA1*01:02-DQB1*06:02	1.30		
		DPA1*02:01-DPB1*14:01	0.60		
CAQKFNGLTVLPPLL (851)	FNGLTVLPP	DRB1*04:01	1.30	-0.08378	0.0846
		DRB1*04:05	0.40		
IRAAEIRASANLAAT (1013)	IRASANLAA	DRB1*04:01	1.40	0.28381	0.6785
		DRB1*08:02	1.20		
		DRB1*13:02	1.90		
		DPA1*02:01-DPB1*14:01	0.80		
		DQA1*01:02-DQB1*06:02	0.30		
		DQA1*05:01-DQB1*03:01	1.00		
DLFLPFFSNVTWFHA (53)	FLPFFSNVT	DRB1*04:01	1.40	0.48085	0.2472
		DRB1*04:05	0.70		
		DPA1*01:03-DPB1*02:01	1.90		
		DPA1*01:03-DPB1*04:01	2.00		
		DRB1*15:01	1.20		
LFLPFFSNVTWFHAI(54)	FFSNVTWFH	DRB1*04:01	1.40	0.4999	0.2477
		DRB3*02:02	2.00		
		DPA1*01:03-DPB1*04:01	1.50		
		DPA1*01:03-DPB1*02:01	1.30		
	FLPFFSNVT	DRB1*04:05	0.80		
		DRB1*15:01	1.20		
QSLLIVNNATNVVIK (115)	LLIVNNATN	DRB1*04:01	1.50	0.3635	0.4343
	LIVNNATNV	DRB1*13:02	0.01		
		DRB3*02:02	0.10		
YQTSNFRVQPTESIV (313)	FRVQPTESI	DRB1*04:01	1.60	-0.00898	0.7563
TQSLLIVNNATNVVI (114)	LLIVNNATN	DRB1*04:01	1.80	0.21031	0.4333
	LIVNNATNV	DRB1*13:02	0.01		
		DRB3*02:02	0.17		
NTLVKQLSSNFGAIS (960)	VKQLSSNFG	DRB1*04:01	1.80	-0.36748	0.2488
ADYSVLYNSASFSTF (363)	YSVLYNSAS	DRB1*04:01	1.80	-0.43901	0.2252
		DRB3*02:02	1.10		
LNTLVKQLSSNFGAI (959)	VKQLSSNFG	DRB1*04:01	1.90	-0.4588	0.0339
QDLFLPFFSNVTWFH (52)	FLPFFSNVT	DRB1*04:01	2.00	0.48528	0.4159
		DRB1*04:05	0.80		
		DRB1*15:01	1.00		
VADYSVLYNSASFST (362)	YSVLYNSAS	DRB1*04:01	2.00	-0.41471	0.2729
		DRB3*02:02	1.90		
ICAQKFNGLTVLPPL (850)	FNGLTVLPP	DRB1*04:05	0.90	-0.13224	0.2298
TQDLFLPFFSNVTWF (51)	FLPFFSNVT	DRB1*04:05	1.60	0.35008	0.3569
		DRB1*15:01	1.40		
IGINITRFQTLLALH (231)	ITRFQTLLA	DRB1*04:05	1.70	0.29879	0.8391
		DRB1*15:01	0.80		
		DRB4*01:01	1.70		
FLPFFSNVTWFHAIH (55)	FFSNVTWFH	DRB1*04:05	1.90	0.60973	0.4883
		DPA1*01:03-DPB1*02:01	1.70		
		DPA1*01:03-DPB1*04:01	1.90		
		DRB3*02:02	1.90		
THWFVTQRNFYEPQI (1100)	FVTQRNFYE	DRB1*04:05	1.90	0.32661	0.3884
PTNFTISVTTEILPV (715)	FTISVTTEI	DRB1*07:01	0.80	0.44474	1.1349
		DQA1*04:01-DQB1*04:02	1.80		

FGEVFNATRFASVYA (338)	FNATRFASV	DRB1*07:01	0.80	0.33709	0.0415
		DRB1*09:01	1.50		
		DPA1*02:01-DPB1*01:01	0.07		
		DPA1*01:03-DPB1*04:01	0.08		
		DPA1*03:01-DPB1*04:02	0.12		
		DPA1*01:03-DPB1*02:01	0.07		
		DPA1*02:01-DPB1*14:01	0.40		
		DPA1*02:01-DPB1*05:01	0.40		
AIPTNFTISVTTEIL (713)	FTISVTTEI	DRB1*07:01	0.90	0.42626	0.6806
		DQA1*04:01-DQB1*04:02	2.00		
GEVFNATRFASVYAW (339)	FNATRFASV	DRB1*07:01	0.90	0.3008	-0.1202
		DRB1*09:01	1.50		
		DPA1*02:01-DPB1*01:01	0.10		
		DPA1*01:03-DPB1*04:01	0.12		
		DPA1*03:01-DPB1*04:02	0.17		
		DPA1*02:01-DPB1*05:01	0.50		
		DPA1*02:01-DPB1*14:01	0.40		
		DQA1*01:02-DQB1*06:02	1.80		
		DPA1*01:03-DPB1*02:01	0.12		
TNFTISVTTEILPVS (716)	FTISVTTEI	DRB1*07:01	0.90	0.39278	1.1691
IPTNFTISVTTEILP (714)	FTISVTTEI	DRB1*07:01	1.00	0.44228	0.8294
		DQA1*04:01-DQB1*04:02	1.80		
PFGEVFNATRFASVY (337)	FNATRFASV	DRB1*07:01	1.10	0.42128	0.0331
		DPA1*02:01-DPB1*01:01	0.12		
		DPA1*01:03-DPB1*04:01	0.12		
		DPA1*03:01-DPB1*04:02	0.20		
		DPA1*02:01-DPB1*05:01	0.60		
		DPA1*02:01-DPB1*14:01	0.80		
		DPA1*01:03-DPB1*02:01	0.12		
EVFNATRFASVYAWN (340)	FNATRFASV	DRB1*07:01	1.20	0.34795	0.0832
		DRB1*09:01	1.60		
		DPA1*02:01-DPB1*01:01	0.20		
		DPA1*01:03-DPB1*04:01	0.25		
		DPA1*03:01-DPB1*04:02	0.30		
		DPA1*02:01-DPB1*05:01	0.90		
		DPA1*02:01-DPB1*14:01	0.50		
		DQA1*01:02-DQB1*06:02	1.40		
		DPA1*01:03-DPB1*02:01	0.30		
VFNATRFASVYAWNR (341)	FNATRFASV	DRB1*07:01	1.20	0.37306	0.0745
		DRB1*09:01	1.30		
		DPA1*01:03-DPB1*02:01	1.20		
		DPA1*03:01-DPB1*04:02	1.30		
		DPA1*01:03-DPB1*04:01	0.90		
		DPA1*02:01-DPB1*14:01	0.60		
		DQA1*01:02-DQB1*06:02	1.30		
		DPA1*02:01-DPB1*01:01	0.90		
LPFFSNVTWFHAIHV (56)	VTWFHAIHV	DRB1*07:01	1.50	0.59565	0.6624
		DPA1*01:03-DPB1*04:01	1.70		
		DPA1*01:03-DPB1*02:01	1.80		
PFFSNVTWFHAIHVS (57)	VTWFHAIHV	DRB1*07:01	1.60	0.51923	0.7656
		DRB1*15:01	1.70		
NFTISVTTEILPVSM (717)	FTISVTTEI	DRB1*07:01	1.70	0.24526	1.2136
YFKIYSKHTPINLVR (200)	YSKHTPINL	DRB1*07:01	2.00	-0.11401	0.8197
QQLIRAAEIRASANL (1010)	IRAAEIRAS	DRB1*08:02	0.25	0.44603	0.1269
		DPA1*02:01-DPB1*14:01	0.25		
		DRB4*01:01	0.25		
		DQA1*01:02-DQB1*06:02	0.06		
		DQA1*05:01-DQB1*03:01	1.10		
QLIRAAEIRASANLA (1011)	IRAAEIRAS	DRB1*08:02	0.40	0.35087	0.2407
		DPA1*02:01-DPB1*14:01	0.40		
		DQA1*05:01-DQB1*03:01	1.00		
		DQA1*01:02-DQB1*06:02	0.07		
		DRB4*01:01	0.50		
LIRAAEIRASANLAA (1012)	IRAAEIRAS	DRB1*08:02	0.60	0.29428	0.2895
		DRB4*01:01	1.10		

		DPA1*02:01-DPB1*14:01	0.50		
		DQA1*01:02-DQB1*06:02	0.07		
		DQA1*05:01-DQB1*03:01	0.90		
VTQQLIRAAEIRASA (1008)	IRAAEIRAS	DRB1*08:02	0.60	0.30342	0.1268
		DRB4*01:01	0.50		
		DPA1*02:01-DPB1*14:01	0.50		
NYNYLYRLFRKSNLK (448)	LYRLFRKSN	DRB1*08:02	1.90	-0.19389	0.1089
		DRB1*11:01	0.12		
		DRB1*15:01	1.60		
		DPA1*02:01-DPB1*05:01	0.40		
		DRB5*01:01	1.30		
YNYLYRLFRKSNLKP (449)	LYRLFRKSN	DRB1*08:02	2.00	-0.32451	0.2254
		DRB1*11:01	0.17		
		DRB1*15:01	1.90		
		DPA1*02:01-DPB1*05:01	0.60		
		DRB5*01:01	1.60		
NYLYRLFRKSNLKPF (450)	LYRLFRKSN	DRB1*08:02	2.00	-0.37964	0.0415
		DRB1*11:01	0.25		
		DRB1*15:01	2.00		
		DRB5*01:01	1.60		
		DPA1*02:01-DPB1*05:01	1.10		
SGWTFGAGAALQIPF (884)	FGAGAALQI	DRB1*09:01	0.25	0.39126	0.2932
		DQA1*03:01-DQB1*03:02	0.50		
		DQA1*04:01-DQB1*04:02	0.30		
		DQA1*01:02-DQB1*06:02	0.90		
		DQA1*05:01-DQB1*03:01	0.12		
WTFGAGAALQIPFAM (886)	FGAGAALQI	DRB1*09:01	0.40	0.33662	0.6670
		DQA1*03:01-DQB1*03:02	0.80		
		DQA1*04:01-DQB1*04:02	0.50		
		DQA1*01:02-DQB1*06:02	0.50		
		DQA1*05:01-DQB1*03:01	0.17		
TSGWTFGAGAALQIP (883)	FGAGAALQI	DRB1*09:01	0.50	0.53555	-0.0178
		DQA1*05:01-DQB1*03:01	0.15		
		DQA1*04:01-DQB1*04:02	0.80		
		DQA1*03:01-DQB1*03:02	1.20		
		DQA1*01:02-DQB1*06:02	2.00		
CVNFNFNGLTGTGVL (538)	FNGLTGTGV	DRB1*09:01	1.60	0.39936	1.3281
ITSGWTFGAGAALQI (882)	FGAGAALQI	DRB1*09:01	0.70	0.44989	0.4483
		DQA1*03:01-DQB1*03:02	1.50		
		DQA1*04:01-DQB1*04:02	1.00		
		DQA1*05:01-DQB1*03:01	0.17		
AALQIPFAMQMAYRF (892)	FAMQMAYRF	DRB1*09:01	1.70	-0.24011	0.9108
		DRB1*12:01	1.40		
		DRB5*01:01	1.70		
		DRB4*01:01	1.80		
SQSIIAYTMSLGAEN (689)	YTMSLGAEN	DRB1*09:01	0.90	0.06264	0.6141
TFGAGAALQIPFAMQ (887)	FGAGAALQI	DRB1*09:01	1.30	0.19539	0.6932
		DQA1*03:01-DQB1*03:02	2.00		
		DQA1*04:01-DQB1*04:02	1.70		
		DQA1*01:02-DQB1*06:02	1.00		
		DQA1*05:01-DQB1*03:01	0.40		
AYTMSLGAENSVAYS (694)	YTMSLGAEN	DRB1*09:01	1.40	-0.26508	0.5815
FNATRFASVYAWNRK (342)	FASVYAWNR	DRB1*09:01	1.90	0.40115	0.4491
		DRB5*01:01	1.70		
		DQA1*01:02-DQB1*06:02	1.80		
		DPA1*02:01-DPB1*14:01	1.20		
FNFNGLTGTGVLTES (541)	FNGLTGTGV	DRB1*09:01	1.90	0.31631	0.7797
SNVTWFHAIHVSGTN (60)	FHAIHVSGT	DRB1*09:01	2.00	0.54861	0.7044
GNYNYLYRLFRKSNL (447)	LYRLFRKSN	DRB1*11:01	0.25	-0.16811	0.1801
		DRB1*15:01	1.90		
		DPA1*02:01-DPB1*05:01	0.50		
		DRB5*01:01	1.70		

GGNYNYLYRLFRKSN (446)	LYRLFRKSN	DRB1*11:01	0.40	-0.10646	0.0207
LYRLFRKSNLKPFER (452)	YRLFRKSNL	DRB1*11:01	1.40	-0.2813	-0.0294
YLYRLFRKSNLKPFE (451)	YRLFRKSNL	DRB1*11:01	0.50	-0.32768	0.0814
RFASVYAWNRKRISN (346)	YAWNRKRIS	DRB1*11:01<br>DRB5*01:01	2.00<br>0.80	0.03511	0.4243
VGGNYNYLYRLFRKS (445)	YRLFRKSNL	DRB1*11:01<br>DPA1*02:01-DPB1*05:01	0.90<br>1.10	0.04407	-0.0193
FQTLLALHRSYLTPG (238)	LLALHRSYL	DRB1*11:01<br>DRB1*12:01<br>DRB5*01:01	1.40<br>1.40<br>1.00	-0.04902	0.5789
SLLIVNNATNVVIKV (116)	IVNNATNVV<br>LIVNNATNV	DRB1*13:02<br>DRB3*02:02	0.01<br>0.12	0.2929	0.4707
LLIVNNATNVVIKVC (117)	IVNNATNVV<br>LIVNNATNV	DRB1*13:02<br>DRB3*02:02	0.01<br>0.20	0.18954	0.0998
KTQSLLIVNNATNVV (113)	LIVNNATNV	DRB1*13:02<br>DRB3*02:02	0.04<br>0.40	0.02483	0.6303
LIVNNATNVVIKVCE (118)	IVNNATNVV	DRB1*13:02<br>DRB3*02:02	0.80<br>0.70	0.02483	-0.1194
SKTQSLLIVNNATNV (112)	LIVNNATNV	DRB1*13:02<br>DRB3*02:02	0.40<br>1.30	-0.06365	0.6256
REGVFVSNGTHWFVT (1091)	FVSNGTHWF	DRB1*13:02<br>DRB3*02:02	1.00<br>0.40	0.47307	0.4461
PREGVFVSNGTHWFV (1090)	FVSNGTHWF	DRB1*13:02<br>DRB3*02:02	1.20<br>0.60	0.45019	0.3043
EGVFVSNGTHWFVTQ (1092)	FVSNGTHWF	DRB1*13:02<br>DRB3*02:02	1.30<br>0.40	0.48972	0.2279
GVFVSNGTHWFVTQR (1093)	FVSNGTHWF	DRB1*13:02<br>DRB3*02:02	1.60<br>0.50	0.34808	0.3243
QTLLALHRSYLTPGD (239)	LALHRSYLT	DRB1*15:01	0.90	-0.03408	0.6708
PIGINITRFQTLLAL (230)	ITRFQTLLA	DRB1*15:01	1.30	0.39848	0.8877
TLLALHRSYLTPGDS (240)	LALHRSYLT	DRB1*15:01	1.50	0.00029	0.5942
LKPFERDISTEIYQA (461)	FERDISTEI	DRB3*01:01	0.25	0.44198	-0.0873
NLKPFERDISTEIYQ (460)	FERDISTEI	DRB3*01:01	0.25	0.41305	0.1834
SNLKPFERDISTEIY (459)	FERDISTEI	DRB3*01:01	0.30	0.2194	0.1589
KPFERDISTEIYQAG (462)	FERDISTEI	DRB3*01:01	0.40	0.36577	-0.2733
KSNLKPFERDISTEI (458)	FERDISTEI	DRB3*01:01	0.80	0.11457	0.4014
PFERDISTEIYQAGS (463)	FERDISTEI	DRB3*01:01	1.10	0.27976	-0.2489
RGVYYPDKVFRSSVL (34)	YYPDKVFRS	DRB3*01:01	1.20	-0.25975	0.1574
RISNCVADYSVLYNS (357)	CVADYSVLY	DRB3*01:01	1.30	-0.15465	-0.2994
KRISNCVADYSVLYN (356)	CVADYSVLY	DRB3*01:01	1.40	-0.21953	-0.0895
ISNCVADYSVLYNSA (358)	CVADYSVLY	DRB3*01:01	1.40	-0.19735	-0.3853
TRGVYYPDKVFRSSV (33)	YYPDKVFRS	DRB3*01:01	1.50	-0.18722	0.1670
RKRISNCVADYSVLY (355)	CVADYSVLY	DRB3*01:01	1.70	-0.10025	-0.0343
SNCVADYSVLYNSAS (359)	CVADYSVLY	DRB3*01:01	1.90	-0.17801	-0.1317

GVYYPDKVFRSSVLH (35)	YYPDKVFRS	DRB3*01:01	1.90	-0.28799	-0.0861
YSVLYNSASFSTFKC (365)	VLYNSASFS	DRB3*02:02	0.80	-0.32952	0.1176
DYSVLYNSASFSTFK (364)	VLYNSASFS	DRB3*02:02	0.90	-0.25753	0.2080
NQKLIANQFNSAIGK (919)	LIANQFNSA	DRB3*02:02	1.00	0.08299	-0.1261
VFVSNGTHWFVTQRN (1094)	FVSNGTHWF	DRB3*02:02	1.10	0.28185	0.3403
QKLIANQFNSAIGKI (920)	LIANQFNSA	DRB3*02:02	1.30	0.0619	-0.3490
ENQKLIANQFNSAIG (918)	LIANQFNSA	DRB3*02:02	1.40	-0.15405	0.0952
QMAYRFNGIGVTQNV (901)	YRFNGIGVT	DRB3*02:02	1.50	0.33788	1.0386
YENQKLIANQFNSAI (917)	LIANQFNSA	DRB3*02:02	1.50	-0.29971	-0.0752
SVLYNSASFSTFKCY (366)	VLYNSASFS	DRB3*02:02	1.80	-0.41032	0.1871
FPREGVFVSNGTHWF (1089)	FVSNGTHWF	DRB3*02:02	1.80	0.40381	0.40381
NSVAYSNNSIAIPTN (703)	AYSNNSIAI	DRB3*02:02	1.90	0.02067	0.3883
MQMAYRFNGIGVTQN (900)	YRFNGIGVT	DRB3*02:02	1.90	0.33641	1.3025
VKQLSSNFGAISSVL (963)	LSSNFGAIS	DRB3*02:02	1.90	-0.33379	0.4397
ENSVAYSNNSIAIPT (702)	AYSNNSIAI	DRB3*02:02	1.90	-0.04019	0.3785
MAYRFNGIGVTQNVL (902)	YRFNGIGVT	DRB3*02:02	2.00	0.3418	0.8498
FIEDLLFNKVTLADA (817)	LLFNKVTLA	DRB3*02:02	2.00	0.04446	0.2786
LVKQLSSNFGAISSV (962)	LSSNFGAIS	DRB3*02:02	2.00	-0.45176	0.4079
TQQLIRAAEIRASAN (1009)	IRAAEIRAS	DRB4*01:01<br>DQA1*05:01-DQB1*03:01<br>DQA1*01:02-DQB1*06:02<br>DPA1*02:01-DPB1*14:01	0.40<br>1.30<br>0.09<br>0.40	0.40501	0.0522
YVTQQLIRAAEIRAS (1007)	IRAAEIRAS	DRB4*01:01<br>DQA1*01:02-DQB1*06:02<br>DPA1*02:01-DPB1*14:01	1.30<br>0.30<br>1.40	0.31796	0.1896
ATRFASVYAWNRKRI (344)	FASVYAWNR	DRB5*01:01<br>DPA1*02:01-DPB1*14:01	0.50<br>1.90	0.19266	0.3489
TRFASVYAWNRKRIS (345)	FASVYAWNR	DRB5*01:01	0.50	0.16921	0.4963
NATRFASVYAWNRKR (343)	FASVYAWNR	DRB5*01:01	0.70	0.25767	0.4062
PFAMQMAYRFNGIGV (897)	FAMQMAYRF	DRB5*01:01	2.00	-0.09118	1.2888
KRSFIEDLLFNKVTL (814)	FIEDLLFNK	DPA1*02:01-DPB1*01:01<br>DPA1*01:03-DPB1*04:01<br>DPA1*03:01-DPB1*04:02<br>DPA1*01:03-DPB1*02:01<br>DPA1*02:01-DPB1*05:01	0.25<br>0.60<br>0.25<br>0.50<br>0.40	0.25342	0.0572
NCTFEYVSQPFLMDL (165)	YVSQPFLMD	DPA1*02:01-DPB1*01:01<br>DPA1*01:03-DPB1*04:01<br>DPA1*03:01-DPB1*04:02<br>DPA1*01:03-DPB1*02:01	0.25<br>0.40<br>0.25<br>0.40	-0.05188	0.5206
SKRSFIEDLLFNKVT (813)	FIEDLLFNK	DPA1*02:01-DPB1*01:01<br>DPA1*01:03-DPB1*02:01<br>DPA1*03:01-DPB1*04:02<br>DPA1*02:01-DPB1*05:01<br>DPA1*01:03-DPB1*04:01	0.30<br>0.70<br>0.30<br>0.50<br>0.60	0.14146	0.0428

CPFGEVFNATRFASV (366)	FNATRFASV	DPA1*02:01-DPB1*01:01	0.30	0.49036	0.2975
CTFEYVSQPFLMDLE (166)	YVSQPFLMD	DPA1*02:01-DPB1*01:01	0.30	-0.15921	0.5700
		DPA1*01:03-DPB1*04:01	0.40		
		DPA1*03:01-DPB1*04:02	0.30		
		DPA1*01:03-DPB1*02:01	0.40		
PSKRSFIEDLLFNKV (812)	FIEDLLFNK	DPA1*02:01-DPB1*01:01	0.40	0.14082	0.0055
		DPA1*01:03-DPB1*02:01	0.80		
		DPA1*03:01-DPB1*04:02	0.40		
		DPA1*02:01-DPB1*05:01	0.70		
		DPA1*01:03-DPB1*04:01	0.80		
TFEYVSQPFLMDLEG (167)	YVSQPFLMD	DPA1*02:01-DPB1*01:01	0.40	-0.2135	0.4481
		DPA1*03:01-DPB1*04:02	0.40		
		DPA1*01:03-DPB1*02:01	0.60		
		DPA1*01:03-DPB1*04:01	0.50		
QPYRVVVLSFELLHA (506)	VLSFELLHA	DPA1*02:01-DPB1*01:01	0.70	0.21018	0.9109
		DPA1*01:03-DPB1*04:01	1.10		
		DPA1*03:01-DPB1*04:02	0.50		
		DPA1*02:01-DPB1*05:01	0.80		
		DPA1*01:03-DPB1*02:01	1.10		
RSFIEDLLFNKVTLA (815)	FIEDLLFNK	DPA1*02:01-DPB1*01:01	0.60	0.23226	-0.0341
		DPA1*01:03-DPB1*04:01	0.90		
		DPA1*03:01-DPB1*04:02	0.50		
		DPA1*02:01-DPB1*05:01	0.80		
		DPA1*01:03-DPB1*02:01	0.90		
NNCTFEYVSQPFLMD (164)	YVSQPFLMD	DPA1*02:01-DPB1*01:01	0.70	-0.0168	0.2040
		DPA1*01:03-DPB1*04:01	0.80		
		DPA1*03:01-DPB1*04:02	0.80		
		DPA1*01:03-DPB1*02:01	0.80		
PYRVVVLSFELLHAP (507)	VLSFELLHA	DPA1*02:01-DPB1*01:01	0.80	0.20726	0.8161
		DPA1*01:03-DPB1*02:01	1.30		
		DPA1*03:01-DPB1*04:02	0.60		
		DPA1*02:01-DPB1*05:01	0.80		
		DPA1*01:03-DPB1*04:01	1.30		
FEYVSQPFLMDLEGK (168)	FNATRFASV	DPA1*02:01-DPB1*01:01	0.80	-0.1967	0.8278
		DPA1*01:03-DPB1*04:01	1.00		
		DPA1*03:01-DPB1*04:02	0.80		
		DPA1*01:03-DPB1*02:01	1.40		
YRVVVLSFELLHAPA (508)	VLSFELLHA	DPA1*02:01-DPB1*01:01	1.20	0.17013	0.7072
		DPA1*01:03-DPB1*04:01	1.70		
		DPA1*03:01-DPB1*04:02	0.80		
		DPA1*02:01-DPB1*05:01	0.90		
		DPA1*01:03-DPB1*02:01	1.70		
KPSKRSFIEDLLFNK (811)	FIEDLLFNK	DPA1*02:01-DPB1*01:01	1.30	0.05508	0.0674
VLLSTFLGRSLEVLF (1231)	FLGRSLEVL	DPA1*02:01-DPB1*01:01	1.30	0.00504	0.4105
		DPA1*01:03-DPB1*04:01	1.50		
		DPA1*03:01-DPB1*04:02	1.00		
		DPA1*01:03-DPB1*02:01	1.60		
YQPYRVVVLSFELLH (505)	RVVVLSFEL	DPA1*02:01-DPB1*01:01	1.50	0.18419	0.9711
		DPA1*01:03-DPB1*04:01	1.80		
		DPA1*03:01-DPB1*04:02	1.20		
		DPA1*02:01-DPB1*05:01	1.90		
		DPA1*01:03-DPB1*02:01	1.80		
WVLLSTFLGRSLEVL (1230)	FLGRSLEVL	DPA1*02:01-DPB1*01:01	1.50	-0.01438	0.5622
		DPA1*01:03-DPB1*04:01	1.50		
		DPA1*03:01-DPB1*04:02	1.10		
		DPA1*02:01-DPB1*05:01	2.00		
		DPA1*01:03-DPB1*02:01	1.90		
SFIEDLLFNKVTLAD (816)	FIEDLLFNK	DPA1*02:01-DPB1*01:01	1.90	0.13189	0.3222
		DPA1*03:01-DPB1*04:02	1.50		
		DPA1*01:03-DPB1*04:01	2.00		
LLSTFLGRSLEVLFQ (1232)	FLGRSLEVL	DPA1*02:01-DPB1*01:01	2.00	0.19546	0.4089
		DPA1*03:01-DPB1*04:02	1.50		
CPFGEVFNATRFASV (336)	FNATRFASV	DPA1*01:03-DPB1*02:01	0.30	0.49036	0.2975
		DPA1*03:01-DPB1*04:02	0.60		
		DPA1*02:01-DPB1*05:01	1.90		

		DPA1*01:03-DPB1*04:01	0.30		
		DPA1*02:01-DPB1*14:01	2.00		
DGEWVLLSTFLGRSL (1227)	VLLSTFLGR	DPA1*01:03-DPB1*02:01	1.80	0.23281	0.6331
		DPA1*03:01-DPB1*04:02	1.40		
		DPA1*02:01-DPB1*05:01	1.70		
		DPA1*01:03-DPB1*04:01	1.50		
GEWVLLSTFLGRSLE (1228)	VLLSTFLGR	DPA1*01:03-DPB1*02:01	2.00	0.00736	0.6378
		DPA1*03:01-DPB1*04:02	1.40		
		DPA1*02:01-DPB1*05:01	1.60		
		DPA1*01:03-DPB1*04:01	1.70		
ANNCTFEYVSQPFLM (163)	FEYVSQPFL	DPA1*01:03-DPB1*02:01	2.00	0.03753	0.1791
EWVLLSTFLGRSLEV (1229)	VLLSTFLGR	DPA1*01:03-DPB1*02:01	2.00	-0.05185	0.7304
		DPA1*03:01-DPB1*04:02	1.50		
		DPA1*02:01-DPB1*05:01	1.70		
		DPA1*01:03-DPB1*04:01	1.70		
RVVVLSFELLHAPAT (509)	VLSFELLHA	DPA1*03:01-DPB1*04:02	1.60	0.15477	0.7485
GYQPYRVVVLSFELL (504)	RVVVLSFEL	DPA1*03:01-DPB1*04:02	1.70	0.13496	1.0740
QGNFKNLREFVFKNI (183)	NLREFVFKN	DPA1*02:01-DPB1*05:01	1.50	0.11512	0.1511
GNFKNLREFVFKNID (184)	NLREFVFKN	DPA1*02:01-DPB1*05:01	1.70	0.0893	0.3930
YYVGYLQPRTFLLKY (265)	LQPRTFLLK	DPA1*02:01-DPB1*05:01	1.90	-0.0341	0.3834
EIRASANLAATKMSE (1017)	IRASANLAA	DPA1*02:01-DPB1*14:01	1.30	-0.41779	0.7813
		DQA1*01:02-DQB1*06:02	1.70		
PQGFSALEPLVDLPI (217)	FSALEPLVD	DQA1*04:01-DQB1*04:02	1.50	0.11614	0.3190
		DQA1*05:01-DQB1*02:01	1.00		
LPQGFSALEPLVDLP (216)	FSALEPLVD	DQA1*05:01-DQB1*02:01	1.30	0.1037	0.1665
		DQA1*04:01-DQB1*04:02	1.00		
		DQA1*03:01-DQB1*03:02	1.50		
DLPQGFSALEPLVDL (215)	FSALEPLVD	DQA1*05:01-DQB1*02:01	1.40	0.0171	0.4812
		DQA1*04:01-DQB1*04:02	0.90		
		DQA1*03:01-DQB1*03:02	1.30		
QGFSALEPLVDLPIG (218)	FSALEPLVD	DQA1*05:01-DQB1*02:01	1.70	0.09449	0.6177
NDLCFTNVYADSFVI (388)	FTNVYADSF	DQA1*05:01-DQB1*02:01	1.70	0.15114	0.6466
DLCFTNVYADSFVIR (389)	FTNVYADSF	DQA1*05:01-DQB1*02:01	1.90	0.2898	0.6743
SSGWTAGAAAYYVGY (255)	TAGAAAYYV	DQA1*05:01-DQB1*03:01	0.10	0.50465	0.4136
		DQA1*04:01-DQB1*04:02	0.80		
		DQA1*01:02-DQB1*06:02	0.30		
		DQA1*03:01-DQB1*03:02	1.10		
SSSGWTAGAAAYYVG (254)	TAGAAAYYV	DQA1*05:01-DQB1*03:01	0.10	0.43682	0.3467
		DQA1*04:01-DQB1*04:02	0.90		
		DQA1*01:02-DQB1*06:02	0.50		
		DQA1*03:01-DQB1*03:02	1.30		
SGWTAGAAAYYVGYL (256)	TAGAAAYYV	DQA1*05:01-DQB1*03:01	0.12	0.35446	0.4157
		DQA1*04:01-DQB1*04:02	0.80		
		DQA1*01:02-DQB1*06:02	0.25		
		DQA1*03:01-DQB1*03:02	1.10		
DSSSGWTAGAAAYYV (253)	WTAGAAAYY	DQA1*05:01-DQB1*03:01	0.15	0.24518	0.3802
		DQA1*04:01-DQB1*04:02	0.60		
		DQA1*01:02-DQB1*06:02	0.90		
		DQA1*03:01-DQB1*03:02	0.90		
GWTAGAAAYYVGYLQ (257)	TAGAAAYYV	DQA1*05:01-DQB1*03:01	0.25	0.25457	0.5669
		DQA1*01:02-DQB1*06:02	0.50		
		DQA1*04:01-DQB1*04:02	1.30		
TNSPGSASSVASQSI (678)	PGSASSVAS	DQA1*05:01-DQB1*03:01	0.40	-0.74891	0.1456
GDSSSGWTAGAAAYY (252)	GWTAGAAAY	DQA1*05:01-DQB1*03:01	0.40	0.08212	0.3045
QTNSPGSASSVASQS	PGSASSVAS	DQA1*05:01-DQB1*03:01	0.40	-0.72184	0.2731

(677)					
NSPGSASSVASQSII (679)	PGSASSVAS	DQA1*05:01-DQB1*03:01<br>DQA1*01:02-DQB1*06:02	0.40<br>1.60	-0.6684	0.0903
SPGSASSVASQSIIA (680)	PGSASSVAS	DQA1*05:01-DQB1*03:01<br>DQA1*01:02-DQB1*06:02	0.50<br>1.10	-0.60082	0.3392
TQTNSPGSASSVASQ (676)	PGSASSVAS	DQA1*05:01-DQB1*03:01	0.60	-0.60279	0.2935
TITSGWTFGAGAALQ (881)	TFGAGAALQ	DQA1*05:01-DQB1*03:01	0.60	0.4141	0.3868
WTAGAAAYYVGYLQP (258)	TAGAAAYYV	DQA1*05:01-DQB1*03:01<br>DQA1*01:02-DQB1*06:02	0.60<br>1.00	0.14658	0.6533
QTQTNSPGSASSVAS (675)	NSPGSASSV	DQA1*05:01-DQB1*03:01	0.80	-0.52587	0.2192
PGSASSVASQSIIAY (681)	SASSVASQS	DQA1*05:01-DQB1*03:01<br>DQA1*01:02-DQB1*06:02	0.80<br>1.00	-0.43277	0.2667
CDIPIGAGICASYQT (662)	PIGAGICAS	DQA1*05:01-DQB1*03:01	0.90	0.15441	0.7935
GTITSGWTFGAGAAL (880)	ITSGWTFGA	DQA1*05:01-DQB1*03:01	0.90	0.48054	0.2715
DIPIGAGICASYQTQ (663)	PIGAGICAS	DQA1*05:01-DQB1*03:01	0.90	0.10996	1.1088
PGDSSSGWTAGAAAY (251)	GWTAGAAAY	DQA1*05:01-DQB1*03:01	0.90	-0.03116	0.2349
ECDIPIGAGICASYQ (661)	PIGAGICAS	DQA1*05:01-DQB1*03:01	1.00	0.33617	0.7566
IPIGAGICASYQTQT (664)	PIGAGICAS	DQA1*05:01-DQB1*03:01	1.10	-0.04506	0.9628
FGAGAALQIPFAMQM (888)	FGAGAALQI	DQA1*05:01-DQB1*03:01<br>DQA1*01:02-DQB1*06:02	1.20<br>1.70	0.05315	0.7854
YECDIPIGAGICASY (660)	PIGAGICAS	DQA1*05:01-DQB1*03:01	1.30	0.37253	0.6775
YTSALLAGTITSGWT (873)	LLAGTITSG	DQA1*05:01-DQB1*03:01	1.40	0.24266	0.3897
QYTSALLAGTITSGW (872)	LLAGTITSG	DQA1*05:01-DQB1*03:01	1.40	0.01785	0.4540
AQYTSALLAGTITSG (871)	YTSALLAGT	DQA1*05:01-DQB1*03:01	1.60	0.06354	0.5382
AGTITSGWTFGAGAA (879)	ITSGWTFGA	DQA1*05:01-DQB1*03:01	1.60	0.54501	0.3368
ALLAGTITSGWTFGA (876)	LLAGTITSG	DQA1*05:01-DQB1*03:01	1.70	0.51671	0.4100
GSASSVASQSIIAYT (682)	SASSVASQS	DQA1*05:01-DQB1*03:01<br>DQA1*01:02-DQB1*06:02	1.70<br>0.90	-0.38583	0.3860
VTQQLIRAAEIRASA (1008)	IRAAEIRAS	DQA1*05:01-DQB1*03:01<br>DQA1*01:02-DQB1*06:02	1.70<br>0.09	0.30342	0.1268
TSALLAGTITSGWTF (874)	LLAGTITSG	DQA1*05:01-DQB1*03:01	1.70	0.34746	0.4136
YQTQTNSPGSASSVA (674)	NSPGSASSV	DQA1*05:01-DQB1*03:01	1.90	-0.60559	0.2297
TQLNRALTGIAVEQD(761)	LTGIAVEQD	DQA1*03:01-DQB1*03:02<br>DQA1*01:02-DQB1*06:02<br>DQA1*04:01-DQB1*04:02	0.30<br>2.00<br>0.30	0.36177	0.4153
QLNRALTGIAVEQDK (762)	LTGIAVEQD	DQA1*03:01-DQB1*03:02<br>DQA1*01:02-DQB1*06:02<br>DQA1*04:01-DQB1*04:02	0.50<br>1.90<br>0.40	0.36543	0.3420
LNRALTGIAVEQDKN (763)	LTGIAVEQD	DQA1*03:01-DQB1*03:02<br>DQA1*04:01-DQB1*04:02	0.70<br>0.60	0.20525	0.4710
NRALTGIAVEQDKNT (764)	LTGIAVEQD	DQA1*03:01-DQB1*03:02<br>DQA1*04:01-DQB1*04:02	1.10<br>0.90	0.10004	0.5298
RALTGIAVEQDKNTQ (765)	LTGIAVEQD	DQA1*03:01-DQB1*03:02<br>DQA1*04:01-DQB1*04:02	1.70<br>1.50	0.09688	0.5206
RDLPQGFSALEPLVD (214)	PQGFSALEP	DQA1*03:01-DQB1*03:02<br>DQA1*04:01-DQB1*04:02	1.90<br>1.40	-0.01254	0.5446
VFLHVTYVPAQEKNF	HVTYVPAQE	DQA1*04:01-DQB1*04:02	1.80	-0.03348	1.0339

(1061)					
GVVFLHVTYVPAQEK (1059)	HVTYVPAQE	DQA1*04:01-DQB1*04:02	2.00	0.25327	1.1043

Supplementary Table 3:

Overlapping HTL and CTL epitopes

EPITOPES	CLASS I SUPERTYPE and ALLELES	CLASS II ALLELES
INITRFQTLLALHRS	B8,B27,B39, HLA-A*30:01, HLA-B*08:01, HLA-A*31:01	DRB1*01:01, DRB1*04:01, DRB1*04:05, DRB1*08:02, DRB1*11:01, DRB1*12:01, DRB1*15:01, DRB4*01:01, DPA1*02:01-DPB1*05:01, DPA1*02:01-DPB1*14:01, DRB5*01:01
GINITRFQTLLALHR	B8,B27,B39 HLA-A*30:01, HLA-B*08:01, HLA-A*31:01	DRB1*01:01, DRB1*04:01, DRB1*04:05, DRB1*11:01, DRB1*12:01. DRB1*15:01, DRB4*01:01, DRB5*01:01, DPA1*03:01-DPB1*04:02, DPA1*02:01-DPB1*05:01, DPA1*02:01-DPB1*14:01, DPA1*02:01-DPB1*01:01
GWTFGAGAALQIPFA	A26,B62 HLA-A*68:02, HLA-A*26:01, HLA-B*51:01, HLA-A*30:01, HLA-B*07:02, HLA-B*15:01,	DRB1*01:01, DRB1*09:01, DQA1*03:01-DQB1*03:02, DQA1*04:01-DQB1*04:02, DQA1*01:02-DQB1*06:02, DQA1*05:01-DQB1*03:01
IRAAEIRASANLAAT	B7,B44, HLA-B*40:01, HLA-B*44:03, HLA-B*44:02, HLA-B*07:02	DRB1*04:01, DRB1*08:02, DRB1*13:02, DPA1*02:01-DPB1*14:01, DQA1*01:02-DQB1*06:02, DQA1*05:01-DQB1*03:01
AAEIRASANLAATKM	A3,B7,B44, HLA-B*40:01, HLA-B*44:03, HLA-B*44:02, HLA-A*11:01, HLA-A*03:01	DRB1*04:01, DRB1*08:02, DRB1*13:01, DPA1*02:01-DPB1*14:01, DQA1*01:02-DQB1*06:02, DRB3*02:02
WTFGAGAALQIPFAM	A26,B7,B58,B62 HLA-B*35:01, HLA-A*68:02, HLA-A*26:01, HLA-B*51:01, HLA-A*30:01, HLA-B*07:02, HLA-B*15:01	DRB1*09:01, DQA1*03:01-DQB1*03:02, DQA1*04:01-DQB1*04:02, DQA1*01:02-DQB1*06:02, DQA1*05:01-DQB1*03:01
QPYRVVVLSFELLHA	A2,A24,B7,B58,B62 HLA-A*23:01, HLA-A*24:02, HLA-A*02:03, HLA-A*02:06, HLA-A*32:01	DPA1*02:01-DPB1*01:01, DPA1*01:03-DPB1*04:01, DPA1*03:01-DPB1*04:02, DPA1*02:01-DPB1*05:01, DPA1*01:03-DPB1*02:01
PYRVVVLSFELLHAP	A2,A24,B7,B58,B62 HLA-A*23:01, HLA-A*24:02, HLA-A*02:03, HLA-A*02:06, HLA-A*32:01	DPA1*02:01-DPB1*01:01, DPA1*01:03-DPB1*02:01, DPA1*03:01-DPB1*04:02, DPA1*02:01-DPB1*05:01, DPA1*01:03-DPB1*04:01

Supplementary Table 4:

IFN Gamma Epitopes as predicted by IFNepitope Server.

EPITOPES	SCORE	ANTIGENICITY
TRFASVYAWNRKRIS	0.73155567	0.4963
GINITRFQTLLALHR	0.10772613	0.5582
INITRFQTLLALHRS	-0.00071534627	0.4118
PINLVRDLPQGFSAL	-0.68072819	0.6086
INLVRDLPQGFSALE	-1.1224405	0.4441

Supplementary Figures

Supplementary Figure S1: Phylogenetic analysis of spike glycoprotein of SARS-CoV-2, isolated from 19 different countries (China, Japan, USA, Australia, Finland, Sweden, India, Colombia, Taiwan, Pakistan, Italy, Israel, Iran, Iran, Vietnam, Peru, Brazil, Spain, Nepal, South Korea) around the globe indicating very low diversity.

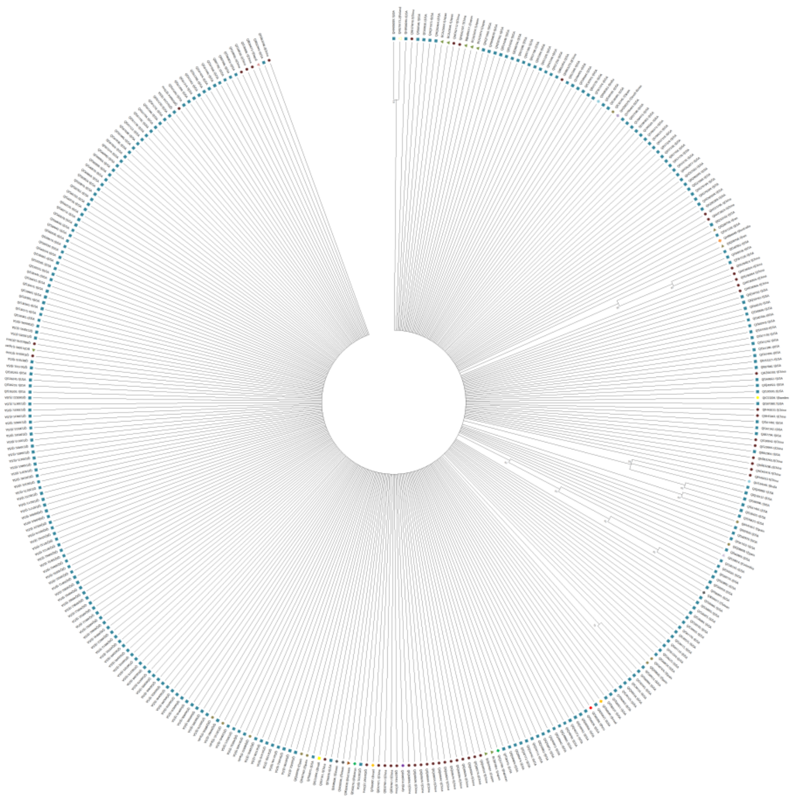

Supplementary Figure S2: Signal Peptide

Measure	Position	Value		
max. C	19	0.107		
max. Y	70	0.106		
max. S	60	0.111		
mean S	1-69	0.097		
D	1-69	0.101	0.450	NO

Name=Sequence SP='NO' D=0.101 D-cutoff=0.450 Networks=SignalP-noTM

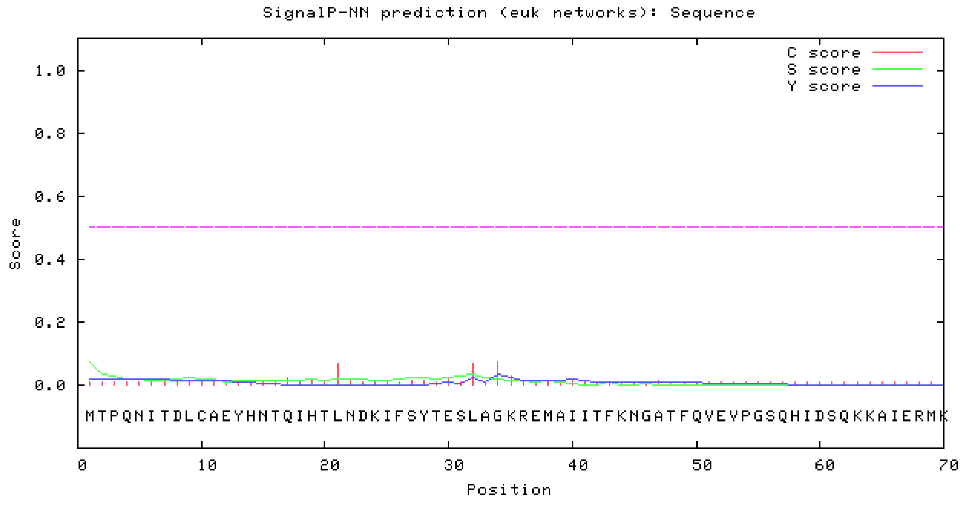

Supplementary Figure S3: Transmembrane Helix

- Length: 422
- Number of predicted TMHs : 0
- Exp number of AAs in TMHs : 0.26093
- Exp number, first 60 AAs : 3e-05
- Total prob of N-in : 0.01678
- TMHMM2.0 outside 1 422

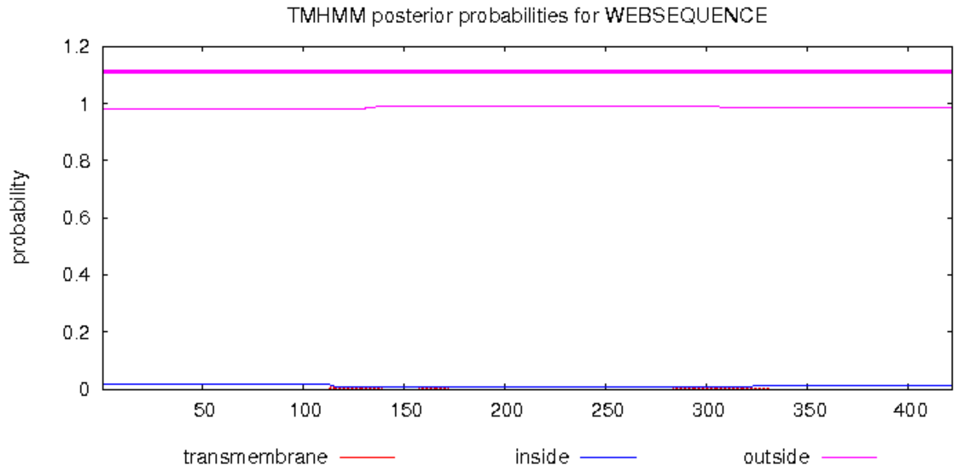

Supplementary Figure S4: B Cell Epitopes

A. Continuous Epitopes predicted by ElliPro and visualized using PyMol. Epitopes are shown in blue colour and the vaccine is shown in hot pink colour

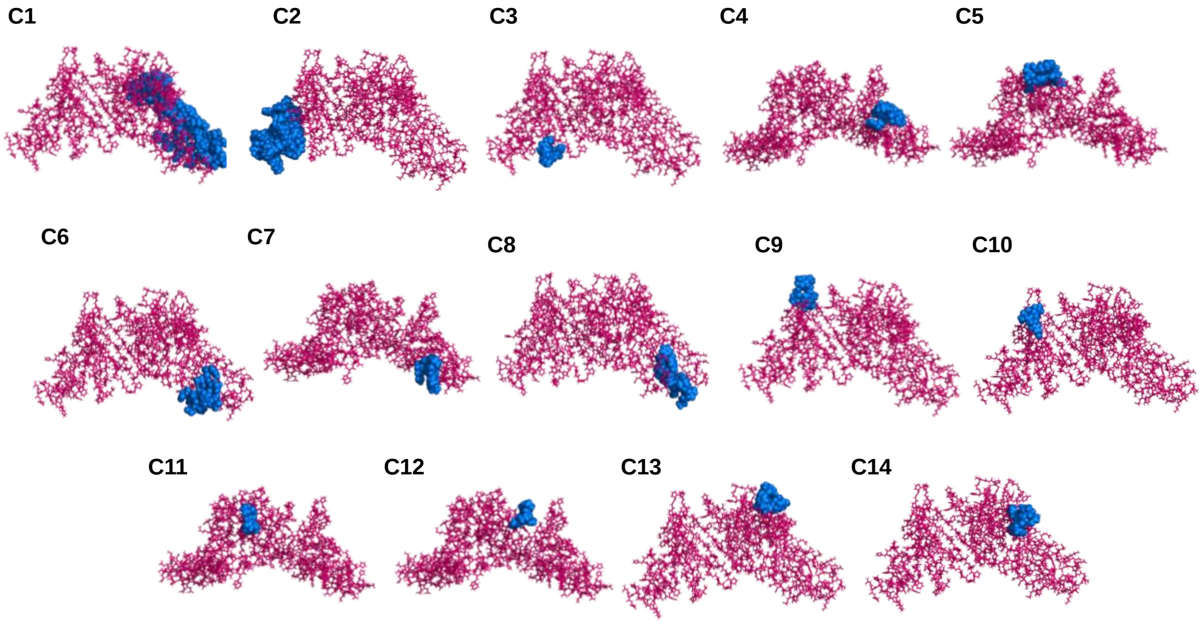

B. Discontinuous Epitopes predicted by ElliPro and visualized using PyMol. Epitopes are shown in green colour and the vaccine is shown in red colour.

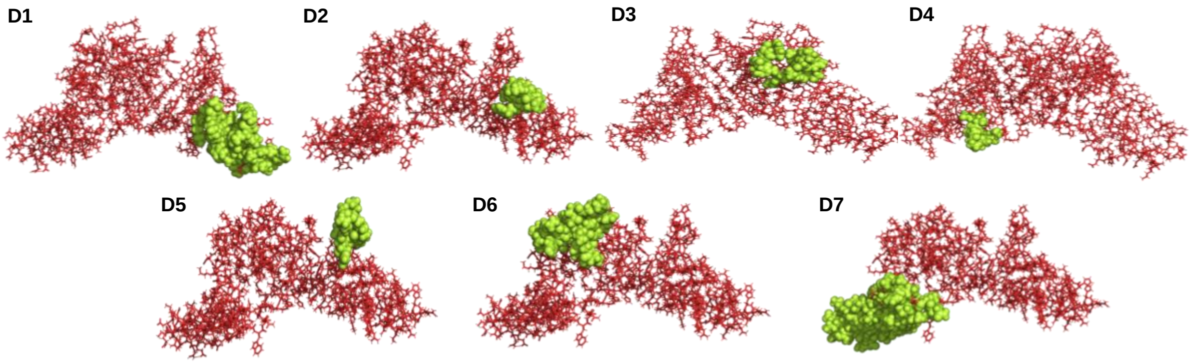

Supplementary Figure S5: Plots showing population coverage.

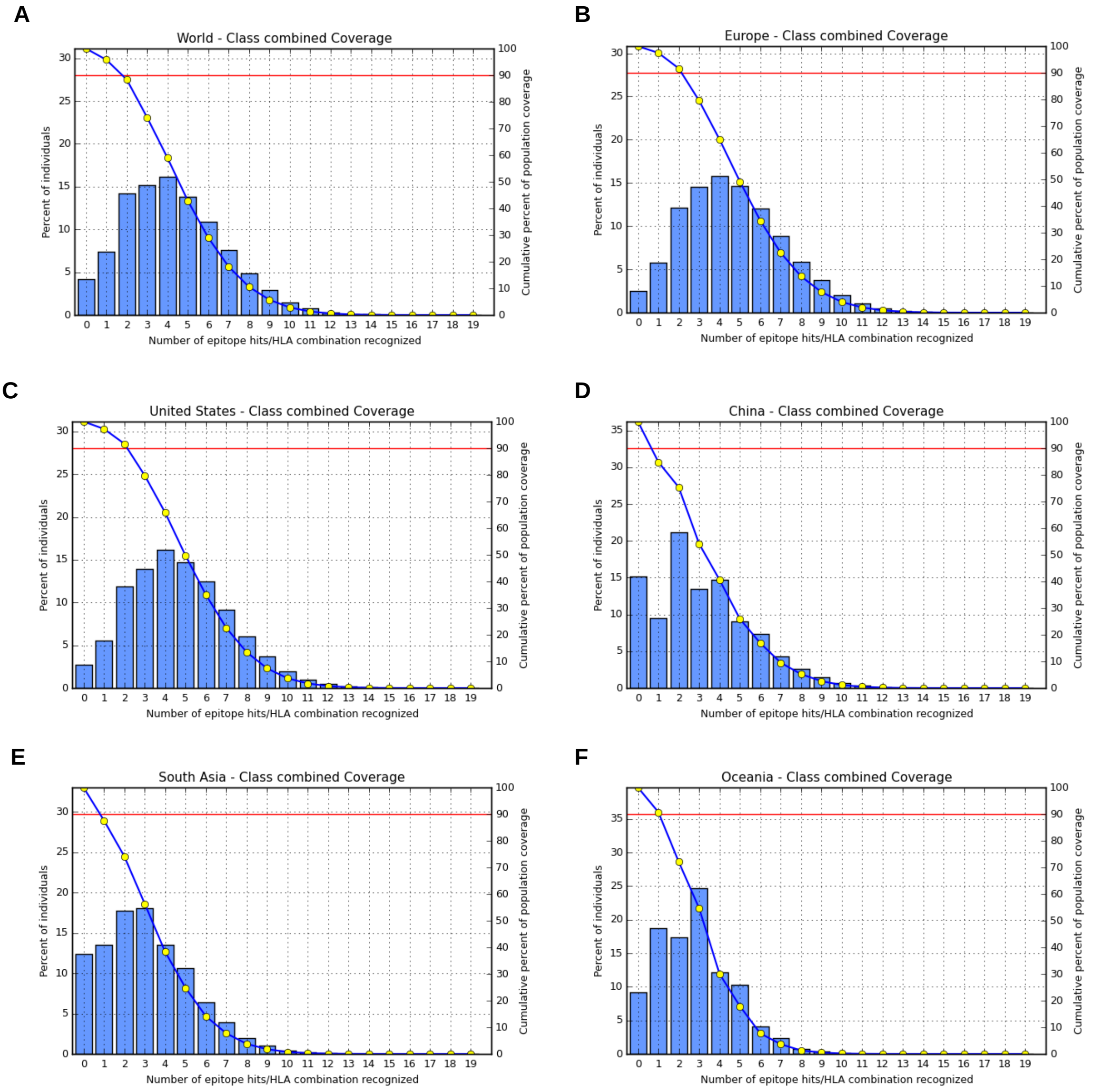

Supplementary Figure S6: Ramachandran plot analysis of vaccine-TLR4 docked complex.

Number of residues in favoured region (~98.0% expected) : 1734 (91.7%)
Number of residues in allowed region (~2.0% expected) : 140 (7.4%)
Number of residues in outlier region : 17 (0.9%)

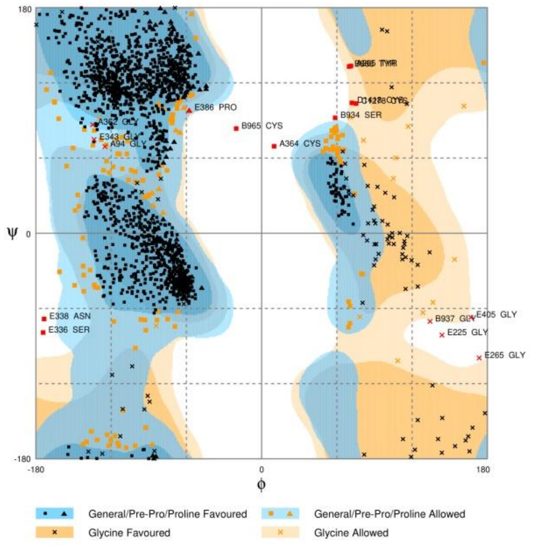

:

Supplementary Figure S7: The results and graphics presented below are based on HADDOCK generated water-refined models of TLR 4 with vaccine construct.

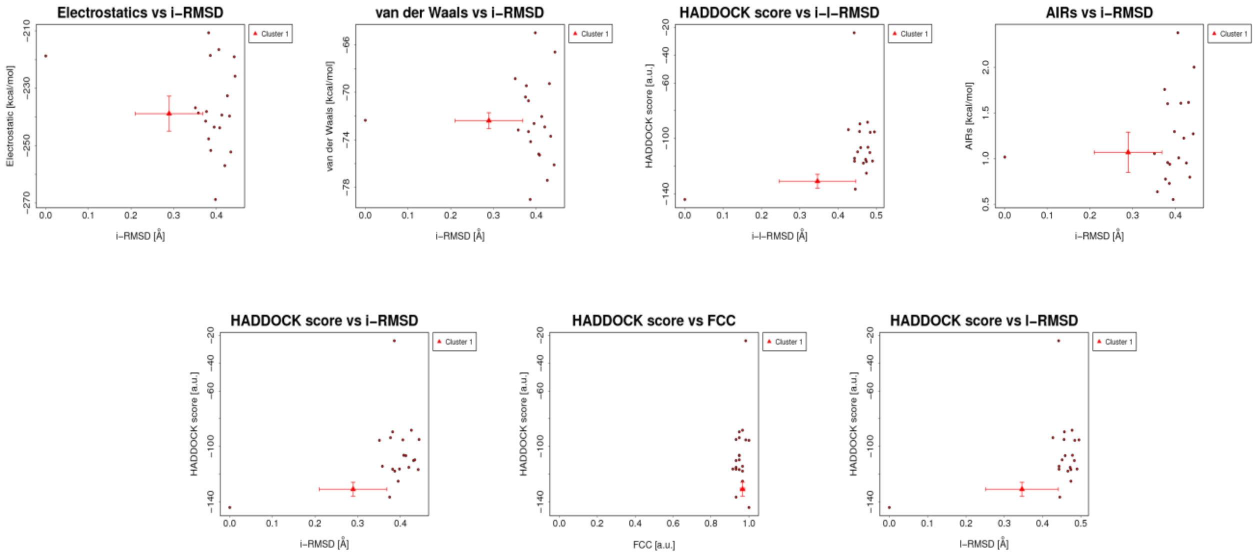

i-RMSD -> interface-RMSD calculated on the backbone (CA,C,N,O,P) atoms of all residues involved in intermolecular contact using a 10Å cutoff.

l-RMSD -> ligand-RMSD calculated on the backbone atoms (CA,C,N,O,P) of all (N>1) molecules after fitting on the backbone atoms of the first (N=1) molecule.

FCC -> Fraction of common contacts. The intermolecular contacts are defined based on the best HADDOCK model using a 5Å cutoff.

a.u. -> Arbitrary Units.

The cluster averages and standard deviations are indicated by colored dots with associated error bars. The average values are calculated on the best 4 structures of each clusters (based on the HADDOCK score).

Supplementary Figure S8: Overview of docked complex TLR4 and vaccine. Hydrogen bonds are shown with black dashes and orange dashes indicate the salt bridges.

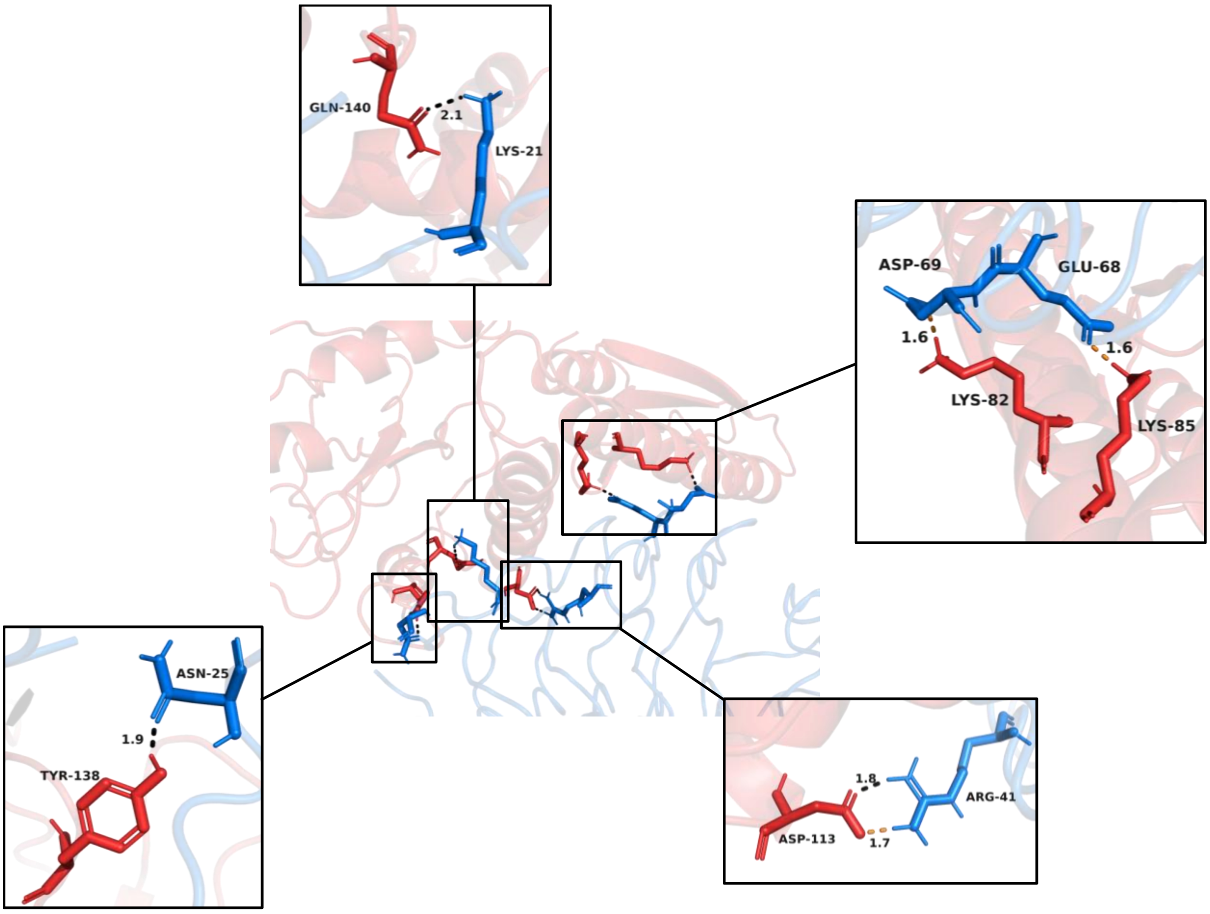

Supplementary Figure S9: Ramachandran plot analysis of vaccine-TLR2 docked complex.

Number of residues in favoured region	(~98.0% expected)	:	854 (88.3%)
Number of residues in allowed region	(~2.0% expected)	:	107 (11.1%)
Number of residues in outlier region		:	6 (0.6%)

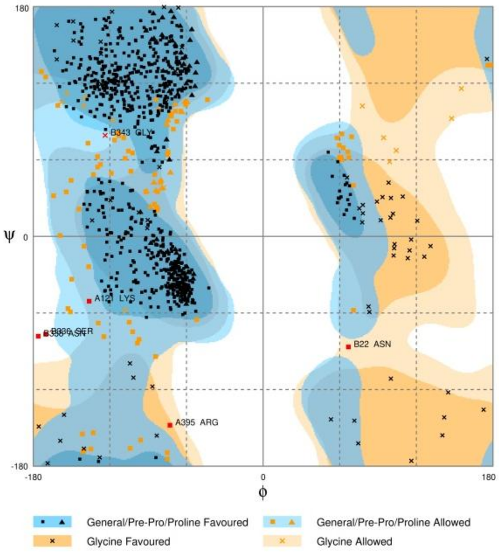

Supplementary Figure S10: The results and graphics presented below are based on HADDOCK generated water-refined models of TLR2 with vaccine construct.

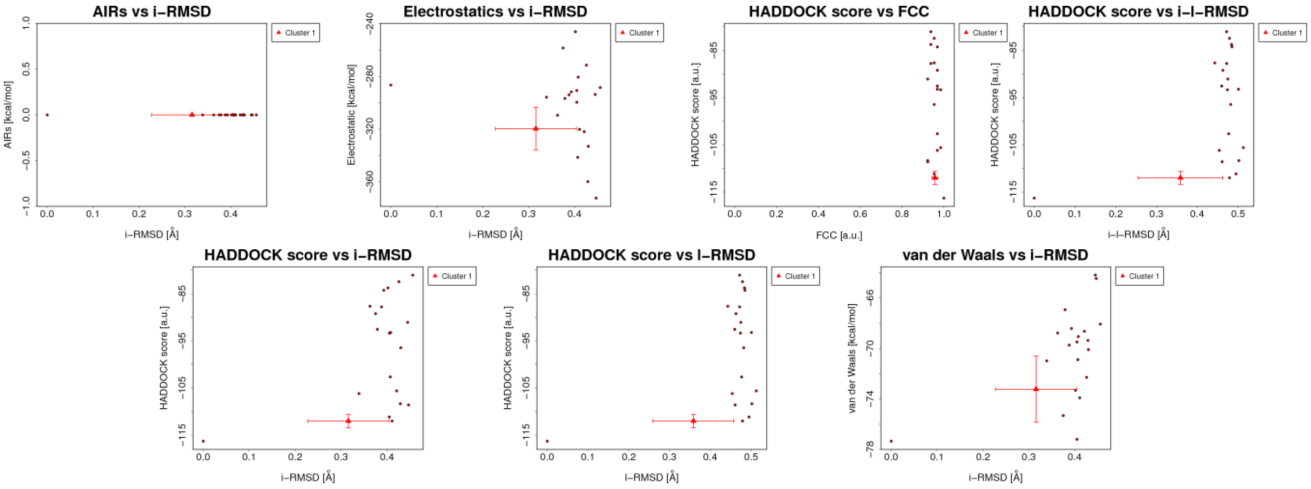

i-RMSD -> interface-RMSD calculated on the backbone (CA,C,N,O,P) atoms of all residues involved in intermolecular contact using a 10Å cutoff.

l-RMSD -> ligand-RMSD calculated on the backbone atoms (CA,C,N,O,P) of all (N>1) molecules after fitting on the backbone atoms of the first (N=1) molecule.

FCC -> Fraction of common contacts. The intermolecular contacts are defined based on the best HADDOCK model using a 5Å cutoff.

a.u. -> Arbitrary Units.

The cluster averages and standard deviations are indicated by colored dots with associated error bars. The average values are calculated on the best 4 structures of each clusters (based on the HADDOCK score).

Supplementary Figure S11: Overview of docked complex TLR2 and vaccine. Hydrogen bonds are shown with black dashes and green dashes indicate salt bridges.

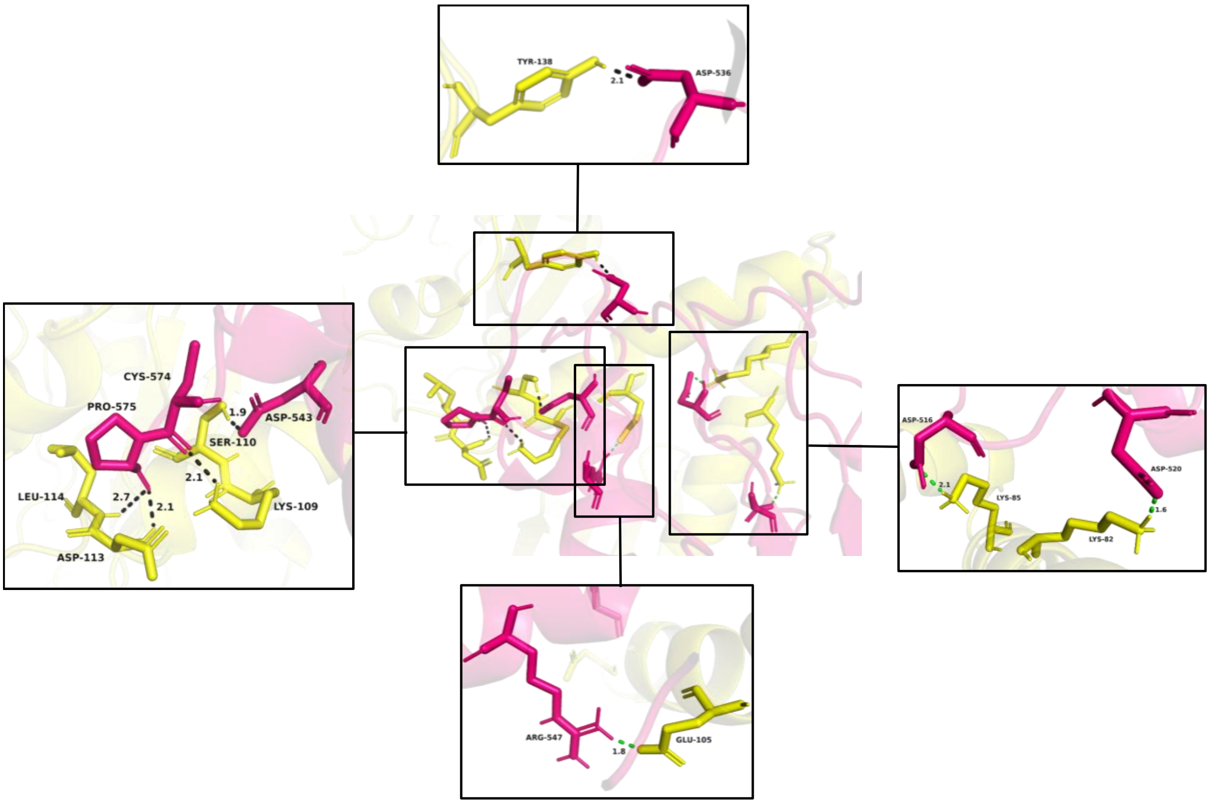

Supplementary Figure S12: Ramachandran plot analysis of vaccine-MHC Class I receptor docked complex.

Number of residues in favoured region	(~98.0% expected)	:	637 (91.9%)
Number of residues in allowed region	(~2.0% expected)	:	46 (6.6%)
Number of residues in outlier region		:	10 (1.4%)

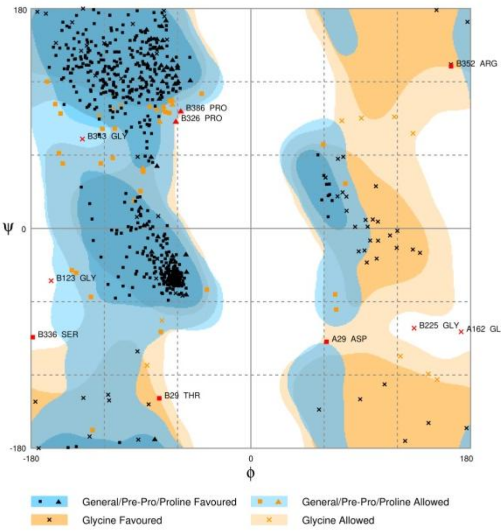

Supplementary Figure S13: The results and graphics presented below are based on HADDOCK generated water-refined models of docked vaccine MHC Class I receptor.

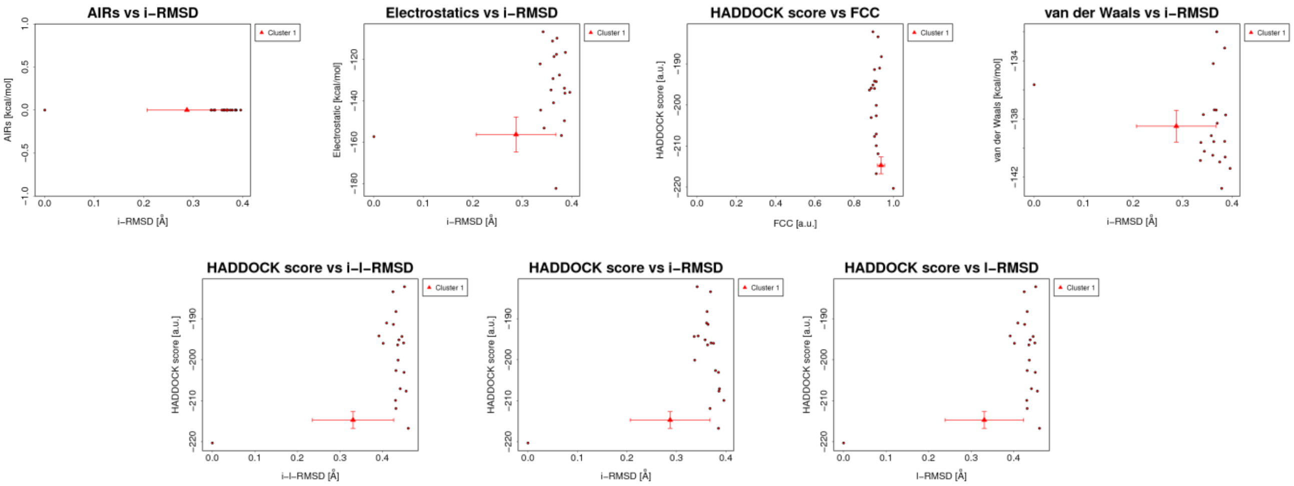

Supplementary Figure S14: Overview of docked vaccine MHC Class I receptor complex. Hydrogen bonds are shown with black dashes and yellow dashes indicate salt bridges.

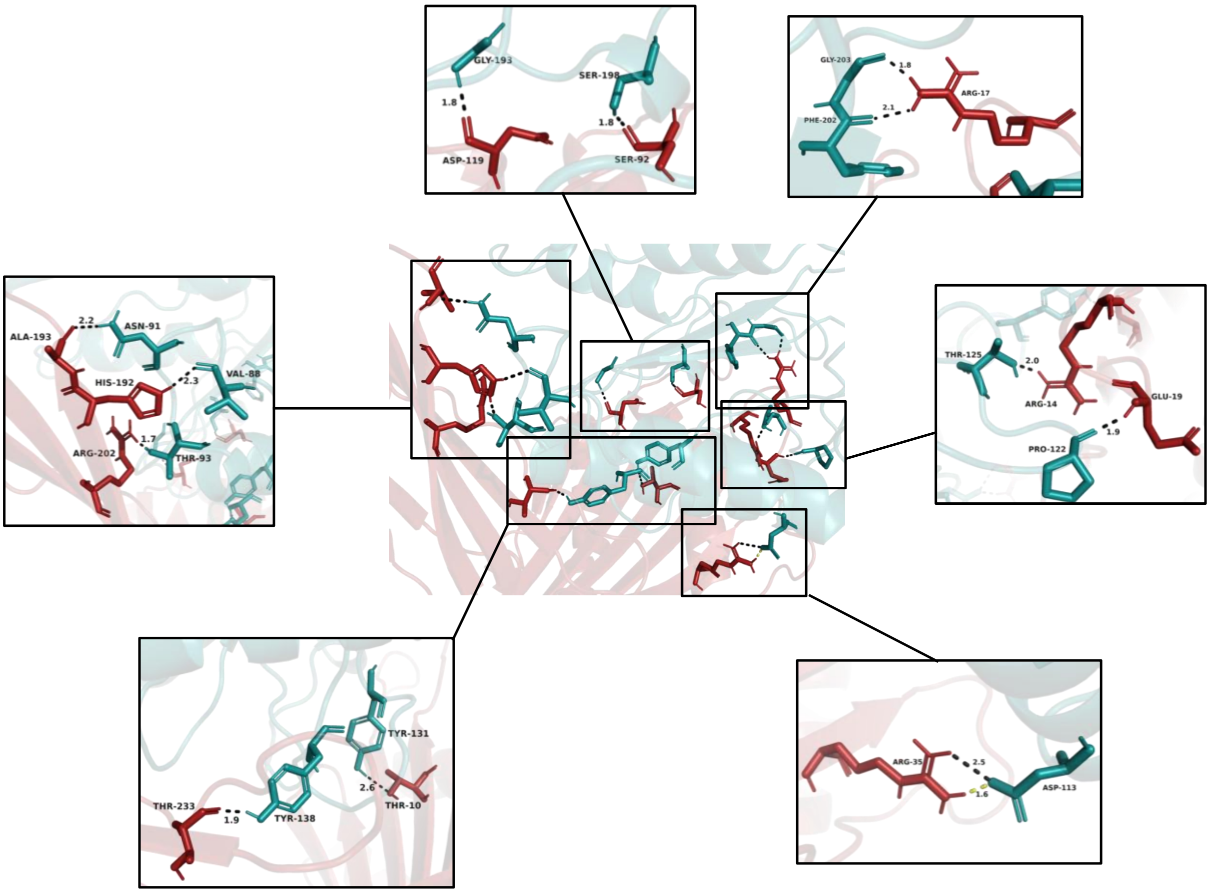

Supplementary Figure S15: Ramachandran plot analysis of vaccine-MHC II docked complex.

Number of residues in favoured region (~98.0% expected)	: 723 (92.2%)
Number of residues in allowed region (~2.0% expected)	: 54 (6.9%)
Number of residues in outlier region	: 7 (0.9%)

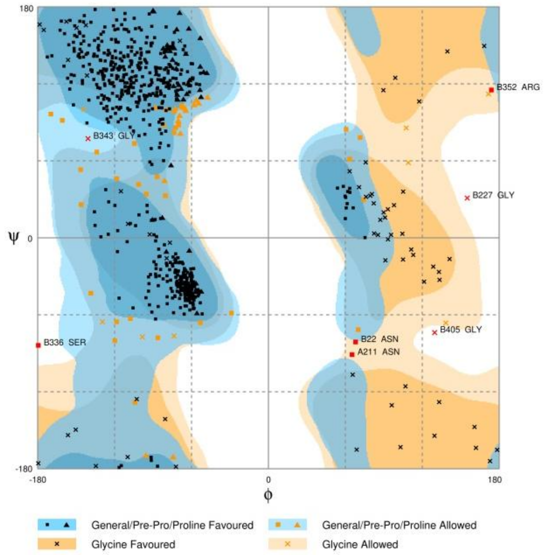

Supplementary Figure S16: The results and graphics presented below are based on HADDOCK generated water-refined models of docked vaccine MHC Class II receptor.

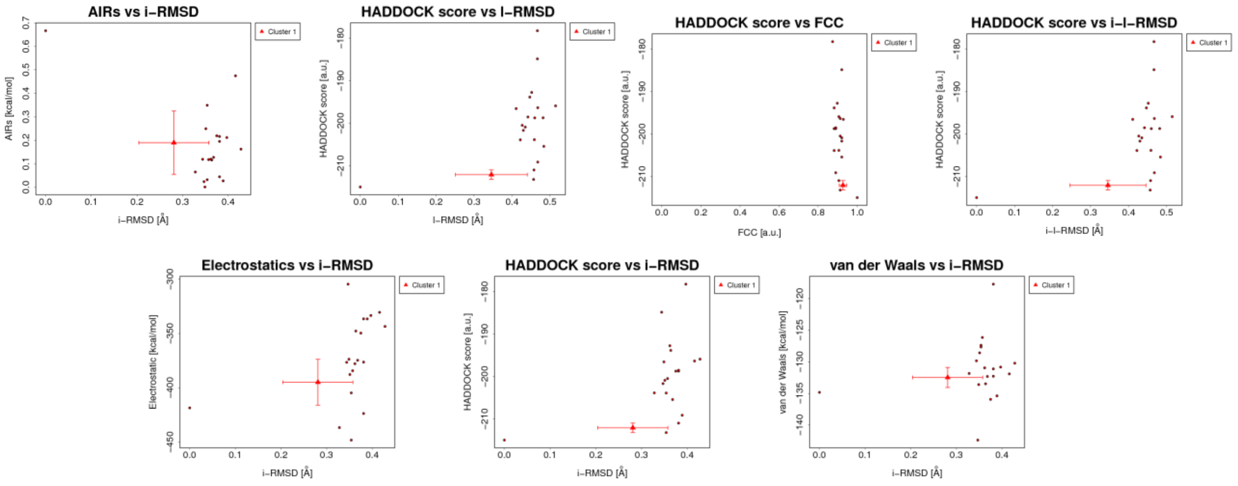

Supplementary Figure S17: Overview of docked vaccine MHC Class II receptor complex. Hydrogen bonds are shown with black dashes and pink dashes indicate salt bridges.

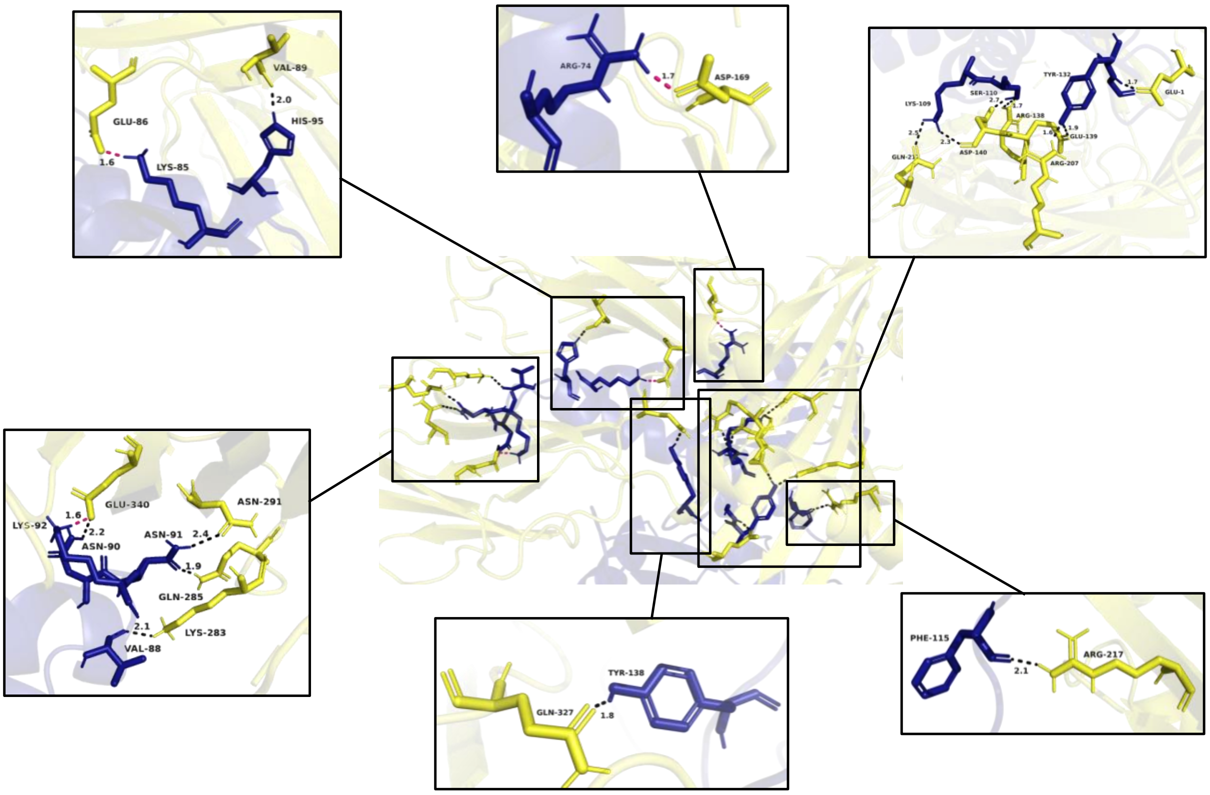

Supplementary Figure S18: Plots showing detailed dynamics of immune simulation variables.

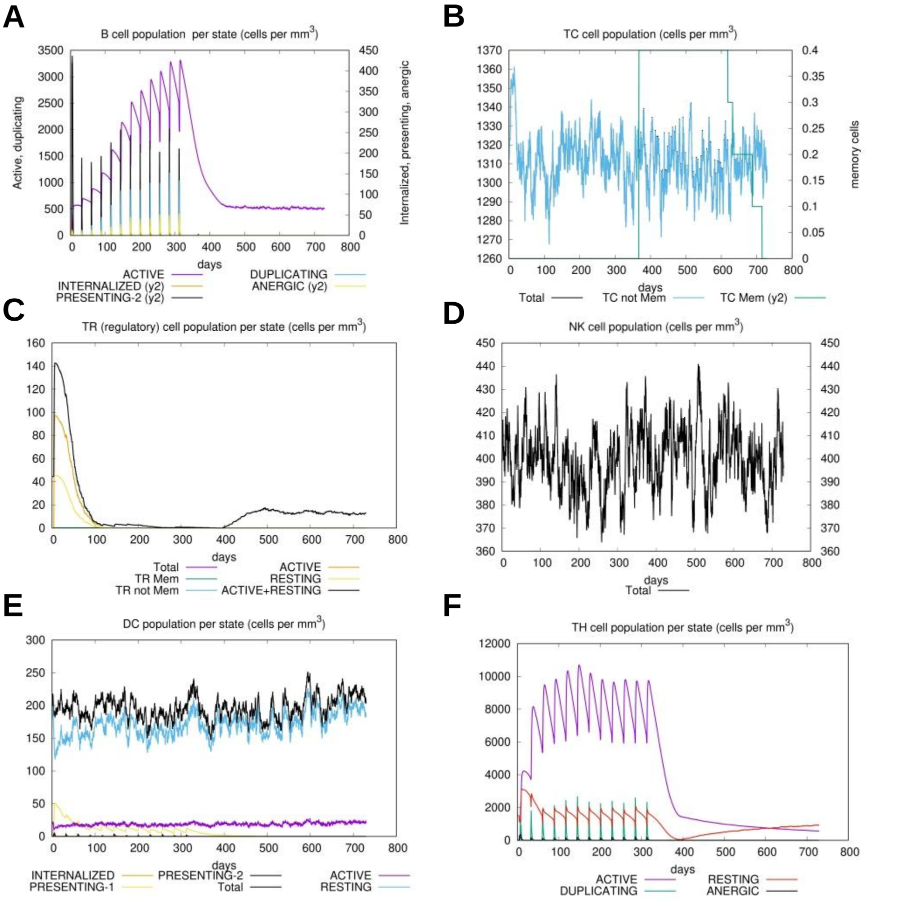

Supplementary Figure S19: Plots of immune simulation variables in the control case corresponding to a “Live virus” which infects the host at day 365 without previous vaccination.

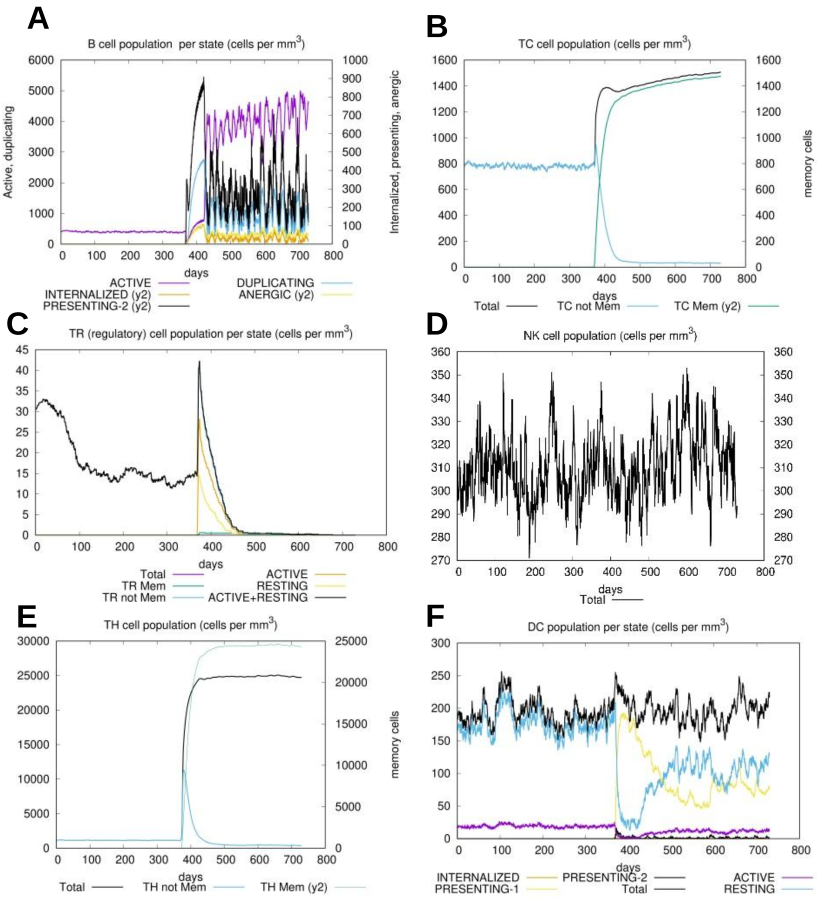

Supplementary materials

Supplementary material SM 1: Physico-chemical property of the candidate vaccine

Number of amino acids: 422

Molecular weight: 44153.58

Theoretical pI: 9.96

Amino acid composition:

Ala (A)	47	11.1%
Arg (R)	21	5.0%
Asn (N)	15	3.6%
Asp (D)	6	1.4%
Cys (C)	2	0.5%
Gln (Q)	17	4.0%
Glu (E)	13	3.1%
Gly (G)	62	14.7%
His (H)	12	2.8%
Ile (I)	28	6.6%
Leu (L)	34	8.1%
Lys (K)	12	2.8%
Met (M)	6	1.4%
Phe (F)	20	4.7%
Pro (P)	46	10.9%
Ser (S)	17	4.0%
Thr (T)	32	7.6%
Trp (W)	6	1.4%
Tyr (Y)	11	2.6%
Val (V)	15	3.6%
Pyl (O)	0	0.0%
Sec (U)	0	0.0%

(B)	0	0.0%
(Z)	0	0.0%
(X)	0	0.0%

Total number of negatively charged residues (Asp + Glu): 19
Total number of positively charged residues (Arg + Lys): 33

Atomic composition:

Carbon	C	2006
Hydrogen	H	3101
Nitrogen	N	559
Oxygen	O	553
Sulfur	S	8

Formula: $C_{2006}H_{3101}N_{559}O_{553}S_{8}$
Total number of atoms: 6227

Extinction coefficients:

Extinction coefficients are in units of M-1 cm-1, at 280 nm measured in water.
Ext. coefficient 49515
Abs 0.1% (=1 g/l) 1.121, assuming all pairs of Cys residues form cystines
Ext. coefficient 49390
Abs 0.1% (=1 g/l) 1.119, assuming all Cys residues are reduced

Estimated half-life:

The N-terminal of the sequence considered is M (Met).

The estimated half-life is: 30 hours (mammalian reticulocytes, in vitro).
>20 hours (yeast, in vivo).
>10 hours (Escherichia coli, in vivo).

Instability index:

The instability index (II) is computed to be 31.04
This classifies the protein as stable.

Aliphatic index: 78.74
Grand average of hydropathicity (GRAVY): -0.088

Supplementary material SM 2:
Vaccine-TLR 4/MD2 PDBsum interacting molecules

Hydrogen bonds

	<----- A T O M 1 ----->						<----- A T O M 2 ----->					
	Atom no.	Atom name	Res name	Res no.	Chain		Atom no.	Atom name	Res name	Res no.	Chain	Distance
1.	200	NZ	LYS	21	A	<-->	15749	OE1	GLN	140	B	2.97
2.	236	OD1	ASN	25	A	<-->	15730	OH	TYR	138	B	2.77
3.	394	NH1	ARG	41	A	<-->	15527	OD1	ASP	113	B	2.70
4.	397	NH2	ARG	41	A	<-->	15528	OD2	ASP	113	B	2.69
5.	423	N	GLY	44	A	<-->	15431	SD	MET	102	B	3.23
6.	663	OE1	GLU	68	A	<-->	15272	NZ	LYS	85	B	2.60
7.	672	OD1	ASP	69	A	<-->	15241	NZ	LYS	82	B	2.67

Non-bonded contacts

	<----- A T O M 1 ----->						<----- A T O M 2 ----->					
	Atom no.	Atom name	Res name	Res no.	Chain		Atom no.	Atom name	Res name	Res no.	Chain	Distance
1.	1	N	GLU	1	A	<-->	15711	CG	PRO	136	B	3.57

2. 155 O LEU 17 A <--> 15697 CA PRO 134 B 3.85
3. 155 O LEU 17 A <--> 15702 O PRO 134 B 3.87
4. 155 O LEU 17 A <--> 15698 CB PRO 134 B 3.55
5. 165 C ASN 18 A <--> 15698 CB PRO 134 B 3.78
6. 166 O ASN 18 A <--> 15698 CB PRO 134 B 3.32
7. 169 CA PHE 19 A <--> 15698 CB PRO 134 B 3.65
8. 172 CD1 PHE 19 A <--> 15698 CB PRO 134 B 3.64
9. 172 CD1 PHE 19 A <--> 15699 CG PRO 134 B 3.64
10. 192 O TYR 20 A <--> 15465 CB ALA 106 B 3.43
11. 182 CB TYR 20 A <--> 15497 OG SER 110 B 3.70
12. 183 CG TYR 20 A <--> 15497 OG SER 110 B 3.64
13. 184 CD1 TYR 20 A <--> 15497 OG SER 110 B 3.77
14. 189 OH TYR 20 A <--> 15537 CD2 LEU 114 B 3.40
15. 197 CG LYS 21 A <--> 15465 CB ALA 106 B 3.76
16. 198 CD LYS 21 A <--> 15749 OE1 GLN 140 B 3.55
17. 199 CE LYS 21 A <--> 15440 O ALA 103 B 3.45
18. 199 CE LYS 21 A <--> 15471 CB ALA 107 B 3.71
19. 199 CE LYS 21 A <--> 15748 CD GLN 140 B 3.87
20. 199 CE LYS 21 A <--> 15749 OE1 GLN 140 B 3.25
21. 199 CE LYS 21 A <--> 15750 NE2 GLN 140 B 3.67
22. 200 NZ LYS 21 A <--> 15471 CB ALA 107 B 3.74
23. 200 NZ LYS 21 A <--> 15748 CD GLN 140 B 3.89
24. 200 NZ LYS 21 A <--> 15749 OE1 GLN 140 B 2.97
25. 212 CD1 ILE 22 A <--> 15432 CE MET 102 B 3.80
26. 216 CA PRO 23 A <--> 15730 OH TYR 138 B 3.86
27. 222 N ASP 24 A <--> 15730 OH TYR 138 B 3.68
28. 227 OD1 ASP 24 A <--> 15730 OH TYR 138 B 3.66
29. 231 N ASN 25 A <--> 15730 OH TYR 138 B 3.56
30. 234 CB ASN 25 A <--> 15730 OH TYR 138 B 3.39
31. 235 CG ASN 25 A <--> 15730 OH TYR 138 B 3.44
32. 236 OD1 ASN 25 A <--> 15729 CZ TYR 138 B 3.76
33. 236 OD1 ASN 25 A <--> 15730 OH TYR 138 B 2.77
34. 393 CZ ARG 41 A <--> 15526 CG ASP 113 B 3.86
35. 393 CZ ARG 41 A <--> 15527 OD1 ASP 113 B 3.40
36. 393 CZ ARG 41 A <--> 15528 OD2 ASP 113 B 3.57
37. 394 NH1 ARG 41 A <--> 15484 CG LYS 109 B 3.32
38. 394 NH1 ARG 41 A <--> 15487 NZ LYS 109 B 3.66
39. 394 NH1 ARG 41 A <--> 15526 CG ASP 113 B 3.44
40. 394 NH1 ARG 41 A <--> 15527 OD1 ASP 113 B 2.70
41. 394 NH1 ARG 41 A <--> 15528 OD2 ASP 113 B 3.59
42. 397 NH2 ARG 41 A <--> 15526 CG ASP 113 B 3.36
43. 397 NH2 ARG 41 A <--> 15527 OD1 ASP 113 B 3.29
44. 397 NH2 ARG 41 A <--> 15528 OD2 ASP 113 B 2.69
45. 409 CE1 HIS 42 A <--> 15206 CD2 LEU 78 B 3.49
46. 423 N GLY 44 A <--> 15431 SD MET 102 B 3.23
47. 425 CA GLY 44 A <--> 15431 SD MET 102 B 3.79
48. 428 N SER 45 A <--> 15270 CD LYS 85 B 3.69
49. 428 N SER 45 A <--> 15271 CE LYS 85 B 3.76
50. 435 O SER 45 A <--> 15270 CD LYS 85 B 3.36
51. 435 O SER 45 A <--> 15271 CE LYS 85 B 3.81

52.	435	O	SER	45	A	<-->	15400	CB	ALA	98	B	3.31
53.	431	CB	SER	45	A	<-->	15270	CD	LYS	85	B	3.69
54.	432	OG	SER	45	A	<-->	15270	CD	LYS	85	B	3.89
55.	439	CB	TYR	46	A	<-->	15400	CB	ALA	98	B	3.60
56.	441	CD1	TYR	46	A	<-->	15376	CD2	HIS	95	B	3.45
57.	442	CD2	TYR	46	A	<-->	15400	CB	ALA	98	B	3.77
58.	443	CE1	TYR	46	A	<-->	15376	CD2	HIS	95	B	3.77
59.	443	CE1	TYR	46	A	<-->	15378	NE2	HIS	95	B	3.81
60.	446	OH	TYR	46	A	<-->	15284	CD2	LEU	86	B	3.79
61.	452	CA	SER	47	A	<-->	15432	CE	MET	102	B	3.61
62.	453	CB	SER	47	A	<-->	15432	CE	MET	102	B	3.87
63.	473	CB	PHE	49	A	<-->	15381	O	HIS	95	B	3.70
64.	473	CB	PHE	49	A	<-->	15373	CB	HIS	95	B	3.44
65.	474	CG	PHE	49	A	<-->	15380	C	HIS	95	B	3.65
66.	474	CG	PHE	49	A	<-->	15381	O	HIS	95	B	3.31
67.	474	CG	PHE	49	A	<-->	15373	CB	HIS	95	B	3.82
68.	475	CD1	PHE	49	A	<-->	15381	O	HIS	95	B	3.23
69.	476	CD2	PHE	49	A	<-->	15380	C	HIS	95	B	3.83
70.	476	CD2	PHE	49	A	<-->	15381	O	HIS	95	B	3.87
71.	476	CD2	PHE	49	A	<-->	15373	CB	HIS	95	B	3.83
72.	476	CD2	PHE	49	A	<-->	15382	N	ALA	96	B	3.90
73.	477	CE1	PHE	49	A	<-->	15381	O	HIS	95	B	3.74
74.	477	CE1	PHE	49	A	<-->	15384	CA	ALA	96	B	3.89
75.	477	CE1	PHE	49	A	<-->	15406	CB	ALA	99	B	3.60
76.	478	CE2	PHE	49	A	<-->	15384	CA	ALA	96	B	3.63
77.	478	CE2	PHE	49	A	<-->	15385	CB	ALA	96	B	3.77
78.	479	CZ	PHE	49	A	<-->	15384	CA	ALA	96	B	3.55
79.	479	CZ	PHE	49	A	<-->	15385	CB	ALA	96	B	3.86
80.	645	CG2	THR	66	A	<-->	15206	CD2	LEU	78	B	3.67
81.	662	CD	GLU	68	A	<-->	15238	CG	LYS	82	B	3.89
82.	662	CD	GLU	68	A	<-->	15272	NZ	LYS	85	B	3.21
83.	663	OE1	GLU	68	A	<-->	15238	CG	LYS	82	B	3.61
84.	663	OE1	GLU	68	A	<-->	15270	CD	LYS	85	B	3.60
85.	663	OE1	GLU	68	A	<-->	15271	CE	LYS	85	B	3.20
86.	663	OE1	GLU	68	A	<-->	15272	NZ	LYS	85	B	2.60
87.	664	OE2	GLU	68	A	<-->	15231	CB	ALA	81	B	3.68
88.	664	OE2	GLU	68	A	<-->	15272	NZ	LYS	85	B	3.06
89.	671	CG	ASP	69	A	<-->	15241	NZ	LYS	82	B	3.59
90.	672	OD1	ASP	69	A	<-->	15240	CE	LYS	82	B	3.40
91.	672	OD1	ASP	69	A	<-->	15241	NZ	LYS	82	B	2.67
92.	717	OG	SER	74	A	<-->	15376	CD2	HIS	95	B	3.46
93.	859	CB	SER	90	A	<-->	15160	NE	ARG	74	B	3.89
94.	860	OG	SER	90	A	<-->	15159	CD	ARG	74	B	3.29
95.	860	OG	SER	90	A	<-->	15160	NE	ARG	74	B	3.43

Salt bridges

	<----- A T O M 1 ----->						<----- A T O M 2 ----->					
	Atom no.	Atom name	Res name	Res no.	Chain		Atom no.	Atom name	Res name	Res no.	Chain	Distance
1.	397	NH2	ARG	41	A	<-->	15528	OD2	ASP	113	B	2.69

2.	663	OE1	GLU	68	A	<-->	15272	NZ	LYS	85	B	2.60
3.	673	OD2	ASP	69	A	<-->	15241	NZ	LYS	82	B	2.67

Supplementary material SM 3:
Vaccine TLR 2 PDBsum interacting molecules

Hydrogen bonds

	<----- A T O M 1 ----->						<----- A T O M 2 ----->					
	Atom no.	Atom name	Res name	Res no.	Chain		Atom no.	Atom name	Res name	Res no.	Chain	Distance
1.	4767	OD2	ASP	516	A	<-->	6190	NZ	LYS	85	B	2.69
2.	4802	OD2	ASP	520	A	<-->	6159	NZ	LYS	82	B	2.68
3.	4977	OD2	ASP	536	A	<-->	6648	OH	TYR	138	B	2.99
4.	5015	NE	ARG	541	A	<-->	6326	O	ALA	99	B	2.91
5.	5021	NH2	ARG	541	A	<-->	6326	O	ALA	99	B	2.99
6.	5041	OD2	ASP	543	A	<-->	6415	OG	SER	110	B	2.63
7.	5347	O	CYS	574	A	<-->	6405	NZ	LYS	109	B	2.91
8.	5356	O	PRO	575	A	<-->	6440	N	ASP	113	B	2.94
9.	5356	O	PRO	575	A	<-->	6449	N	LEU	114	B	2.93

Non-bonded contacts

	<----- A T O M 1 ----->						<----- A T O M 2 ----->					
	Atom no.	Atom name	Res name	Res no.	Chain		Atom no.	Atom name	Res name	Res no.	Chain	Distance
1.	4535	CG2	THR	491	A	<-->	6291	CB	HIS	95	B	3.80
2.	4535	CG2	THR	491	A	<-->	6292	CG	HIS	95	B	3.53
3.	4535	CG2	THR	491	A	<-->	6293	ND1	HIS	95	B	3.46
4.	4746	N	VAL	514	A	<-->	6349	SD	MET	102	B	3.68
5.	4746	N	VAL	514	A	<-->	6350	CE	MET	102	B	3.59
6.	4752	C	VAL	514	A	<-->	6349	SD	MET	102	B	3.80
7.	4753	O	VAL	514	A	<-->	6348	CG	MET	102	B	3.30
8.	4753	O	VAL	514	A	<-->	6349	SD	MET	102	B	3.31
9.	4753	O	VAL	514	A	<-->	6350	CE	MET	102	B	3.40
10.	4761	N	ASP	516	A	<-->	6189	CE	LYS	85	B	3.57
11.	4769	O	ASP	516	A	<-->	6189	CE	LYS	85	B	3.22
12.	4769	O	ASP	516	A	<-->	6190	NZ	LYS	85	B	3.28
13.	4765	CG	ASP	516	A	<-->	6189	CE	LYS	85	B	3.40
14.	4765	CG	ASP	516	A	<-->	6190	NZ	LYS	85	B	3.14
15.	4766	OD1	ASP	516	A	<-->	6189	CE	LYS	85	B	3.08
16.	4766	OD1	ASP	516	A	<-->	6190	NZ	LYS	85	B	3.16
17.	4766	OD1	ASP	516	A	<-->	6342	C	SER	101	B	3.51
18.	4766	OD1	ASP	516	A	<-->	6343	O	SER	101	B	3.41
19.	4766	OD1	ASP	516	A	<-->	6344	N	MET	102	B	3.37
20.	4766	OD1	ASP	516	A	<-->	6346	CA	MET	102	B	3.07
21.	4766	OD1	ASP	516	A	<-->	6347	CB	MET	102	B	3.86
22.	4766	OD1	ASP	516	A	<-->	6348	CG	MET	102	B	3.62
23.	4767	OD2	ASP	516	A	<-->	6189	CE	LYS	85	B	3.48
24.	4767	OD2	ASP	516	A	<-->	6190	NZ	LYS	85	B	2.69
25.	4767	OD2	ASP	516	A	<-->	6343	O	SER	101	B	3.85

26.	4767	OD2	ASP	516	A	<-->	6373	CB	GLU	105	B	3.40
27.	4799	CB	ASP	520	A	<-->	6158	CE	LYS	82	B	3.86
28.	4800	CG	ASP	520	A	<-->	6123	CD1	LEU	78	B	3.82
29.	4800	CG	ASP	520	A	<-->	6159	NZ	LYS	82	B	3.71
30.	4801	OD1	ASP	520	A	<-->	6123	CD1	LEU	78	B	3.48
31.	4802	OD2	ASP	520	A	<-->	6158	CE	LYS	82	B	3.40
32.	4802	OD2	ASP	520	A	<-->	6159	NZ	LYS	82	B	2.68
33.	4810	CD	ARG	521	A	<-->	6158	CE	LYS	82	B	3.83
34.	4977	OD2	ASP	536	A	<-->	6647	CZ	TYR	138	B	3.83
35.	4977	OD2	ASP	536	A	<-->	6648	OH	TYR	138	B	2.99
36.	4990	CB	SER	538	A	<-->	6645	CE1	TYR	138	B	3.66
37.	4990	CB	SER	538	A	<-->	6647	CZ	TYR	138	B	3.58
38.	4991	OG	SER	538	A	<-->	6648	OH	TYR	138	B	3.82
39.	4997	CA	CYS	539	A	<-->	6415	OG	SER	110	B	3.87
40.	4998	C	CYS	539	A	<-->	6415	OG	SER	110	B	3.46
41.	4999	O	CYS	539	A	<-->	6385	O	ALA	106	B	3.87
42.	4999	O	CYS	539	A	<-->	6383	CB	ALA	106	B	3.50
43.	4999	O	CYS	539	A	<-->	6415	OG	SER	110	B	3.38
44.	5000	CB	CYS	539	A	<-->	6415	OG	SER	110	B	3.33
45.	5000	CB	CYS	539	A	<-->	6668	NE2	GLN	140	B	3.38
46.	5001	SG	CYS	539	A	<-->	6618	CD	PRO	134	B	3.66
47.	5002	N	PRO	540	A	<-->	6668	NE2	GLN	140	B	3.45
48.	5007	C	PRO	540	A	<-->	6383	CB	ALA	106	B	3.84
49.	5008	O	PRO	540	A	<-->	6352	O	MET	102	B	3.75
50.	5008	O	PRO	540	A	<-->	6355	CA	ALA	103	B	3.25
51.	5008	O	PRO	540	A	<-->	6356	CB	ALA	103	B	3.60
52.	5004	CB	PRO	540	A	<-->	6666	CD	GLN	140	B	3.79
53.	5004	CB	PRO	540	A	<-->	6667	OE1	GLN	140	B	3.25
54.	5004	CB	PRO	540	A	<-->	6668	NE2	GLN	140	B	3.77
55.	5005	CG	PRO	540	A	<-->	6643	CD1	TYR	138	B	3.78
56.	5005	CG	PRO	540	A	<-->	6668	NE2	GLN	140	B	3.89
57.	5006	CD	PRO	540	A	<-->	6668	NE2	GLN	140	B	3.32
58.	5009	N	ARG	541	A	<-->	6383	CB	ALA	106	B	3.61
59.	5011	CA	ARG	541	A	<-->	6352	O	MET	102	B	3.86
60.	5011	CA	ARG	541	A	<-->	6383	CB	ALA	106	B	3.87
61.	5024	C	ARG	541	A	<-->	6350	CE	MET	102	B	3.82
62.	5024	C	ARG	541	A	<-->	6383	CB	ALA	106	B	3.61
63.	5025	O	ARG	541	A	<-->	6352	O	MET	102	B	3.56
64.	5025	O	ARG	541	A	<-->	6347	CB	MET	102	B	3.37
65.	5025	O	ARG	541	A	<-->	6350	CE	MET	102	B	3.25
66.	5025	O	ARG	541	A	<-->	6383	CB	ALA	106	B	3.54
67.	5013	CG	ARG	541	A	<-->	6350	CE	MET	102	B	3.77
68.	5015	NE	ARG	541	A	<-->	6326	O	ALA	99	B	2.91
69.	5017	CZ	ARG	541	A	<-->	6326	O	ALA	99	B	3.37
70.	5021	NH2	ARG	541	A	<-->	6325	C	ALA	99	B	3.62
71.	5021	NH2	ARG	541	A	<-->	6326	O	ALA	99	B	2.99
72.	5021	NH2	ARG	541	A	<-->	6327	N	ILE	100	B	3.82
73.	5021	NH2	ARG	541	A	<-->	6329	CA	ILE	100	B	3.43
74.	5031	CG2	ILE	542	A	<-->	6350	CE	MET	102	B	3.77
75.	5035	N	ASP	543	A	<-->	6383	CB	ALA	106	B	3.64

76. 5038 CB ASP 543 A <--> 6382 CA ALA 106 B 3.76
77. 5038 CB ASP 543 A <--> 6385 O ALA 106 B 3.45
78. 5038 CB ASP 543 A <--> 6383 CB ALA 106 B 3.64
79. 5039 CG ASP 543 A <--> 6385 O ALA 106 B 3.80
80. 5039 CG ASP 543 A <--> 6402 CG LYS 109 B 3.66
81. 5039 CG ASP 543 A <--> 6415 OG SER 110 B 3.47
82. 5041 OD2 ASP 543 A <--> 6385 O ALA 106 B 3.23
83. 5041 OD2 ASP 543 A <--> 6409 C LYS 109 B 3.41
84. 5041 OD2 ASP 543 A <--> 6401 CB LYS 109 B 3.45
85. 5041 OD2 ASP 543 A <--> 6402 CG LYS 109 B 3.29
86. 5041 OD2 ASP 543 A <--> 6411 N SER 110 B 3.04
87. 5041 OD2 ASP 543 A <--> 6413 CA SER 110 B 3.32
88. 5041 OD2 ASP 543 A <--> 6414 CB SER 110 B 3.49
89. 5041 OD2 ASP 543 A <--> 6415 OG SER 110 B 2.63
90. 5054 OH TYR 544 A <--> 6123 CD1 LEU 78 B 3.75
91. 5054 OH TYR 544 A <--> 6124 CD2 LEU 78 B 3.64
92. 5081 NE ARG 547 A <--> 6403 CD LYS 109 B 3.78
93. 5083 CZ ARG 547 A <--> 6403 CD LYS 109 B 3.70
94. 5084 NH1 ARG 547 A <--> 6377 OE2 GLU 105 B 3.38
95. 5084 NH1 ARG 547 A <--> 6403 CD LYS 109 B 3.77
96. 5087 NH2 ARG 547 A <--> 6434 OE1 GLN 112 B 3.78
97. 5347 O CYS 574 A <--> 6405 NZ LYS 109 B 2.91
98. 5351 CA PRO 575 A <--> 6443 CB ASP 113 B 3.51
99. 5355 C PRO 575 A <--> 6417 C SER 110 B 3.81
100. 5355 C PRO 575 A <--> 6418 O SER 110 B 3.13
101. 5355 C PRO 575 A <--> 6447 C ASP 113 B 3.76
102. 5355 C PRO 575 A <--> 6443 CB ASP 113 B 3.59
103. 5355 C PRO 575 A <--> 6449 N LEU 114 B 3.31
104. 5355 C PRO 575 A <--> 6451 CA LEU 114 B 3.90
105. 5355 C PRO 575 A <--> 6452 CB LEU 114 B 3.66
106. 5355 C PRO 575 A <--> 6455 CD2 LEU 114 B 3.84
107. 5356 O PRO 575 A <--> 6410 O LYS 109 B 3.28
108. 5356 O PRO 575 A <--> 6413 CA SER 110 B 3.73
109. 5356 O PRO 575 A <--> 6417 C SER 110 B 3.25
110. 5356 O PRO 575 A <--> 6418 O SER 110 B 2.88
111. 5356 O PRO 575 A <--> 6440 N ASP 113 B 2.94
112. 5356 O PRO 575 A <--> 6442 CA ASP 113 B 3.25
113. 5356 O PRO 575 A <--> 6447 C ASP 113 B 3.37
114. 5356 O PRO 575 A <--> 6443 CB ASP 113 B 3.11
115. 5356 O PRO 575 A <--> 6449 N LEU 114 B 2.93
116. 5356 O PRO 575 A <--> 6451 CA LEU 114 B 3.89
117. 5356 O PRO 575 A <--> 6452 CB LEU 114 B 3.81
118. 5352 CB PRO 575 A <--> 6455 CD2 LEU 114 B 3.59
119. 5353 CG PRO 575 A <--> 6605 OH TYR 132 B 3.33
120. 5354 CD PRO 575 A <--> 6603 CE2 TYR 132 B 3.61
121. 5354 CD PRO 575 A <--> 6604 CZ TYR 132 B 3.89
122. 5354 CD PRO 575 A <--> 6605 OH TYR 132 B 3.32

Salt bridges

<----- A T O M 1 -----> <----- A T O M 2 ----->

	Atom no.	Atom name	Res name	Res no.	Chain		Atom no.	Atom name	Res name	Res no.	Chain	Distance
1.	4766	OD1	ASP	516	A	<-->	6190	NZ	LYS	85	B	2.69
2.	4802	OD2	ASP	520	A	<-->	6159	NZ	LYS	82	B	2.68
3.	5084	NH1	ARG	547	A	<-->	6377	OE2	GLU	105	B	3.38

Supplementary material SM 4:
Vaccine-MHC I PDBsum interacting molecules

Hydrogen bonds

	Atom no.	Atom name	Res name	Res no.	Chain		Atom no.	Atom name	Res name	Res no.	Chain	Distance
1.	102	OG1	THR	10	A	<-->	4002	OH	TYR	131	B	3.34
2.	140	NH1	ARG	14	A	<-->	3958	OG1	THR	125	B	2.80
3.	172	NH2	ARG	17	A	<-->	4624	O	PHE	202	B	2.86
4.	172	NH2	ARG	17	A	<-->	4629	O	GLY	203	B	2.71
5.	182	N	GLU	19	A	<-->	3932	O	PRO	122	B	2.86
6.	346	NH1	ARG	35	A	<-->	3857	OD2	ASP	113	B	3.22
7.	349	NH2	ARG	35	A	<-->	3857	OD2	ASP	113	B	2.59
8.	924	O	SER	92	A	<-->	4578	OG	SER	198	B	2.71
9.	1213	O	ASP	119	A	<-->	4527	N	GLY	193	B	2.78
10.	1951	NE2	HIS	192	A	<-->	3631	O	VAL	88	B	3.16
11.	1960	O	ALA	193	A	<-->	3665	ND2	ASN	91	B	3.17
12.	2041	NH1	ARG	202	A	<-->	3687	OG1	THR	93	B	2.74
13.	2354	O	THR	233	A	<-->	4059	OH	TYR	138	B	2.80

Non-bonded contacts

<----- A T O M 1 -----> <----- A T O M 2 ----->

	Atom no.	Atom name	Res name	Res no.	Chain		Atom no.	Atom name	Res name	Res no.	Chain	Distance
1.	82	CE2	PHE	8	A	<-->	4040	CG	PRO	136	B	3.74
2.	83	CZ	PHE	8	A	<-->	4040	CG	PRO	136	B	3.65
3.	101	CB	THR	10	A	<-->	4000	CE2	TYR	131	B	3.64
4.	101	CB	THR	10	A	<-->	4002	OH	TYR	131	B	3.73
5.	102	OG1	THR	10	A	<-->	4002	OH	TYR	131	B	3.34
6.	104	CG2	THR	10	A	<-->	4000	CE2	TYR	131	B	3.64
7.	104	CG2	THR	10	A	<-->	4001	CZ	TYR	131	B	3.85
8.	104	CG2	THR	10	A	<-->	4002	OH	TYR	131	B	3.45
9.	130	O	SER	13	A	<-->	4563	CD1	TRP	197	B	3.21
10.	130	O	SER	13	A	<-->	4565	NE1	TRP	197	B	3.36
11.	133	CA	ARG	14	A	<-->	4565	NE1	TRP	197	B	3.59
12.	134	CB	ARG	14	A	<-->	4565	NE1	TRP	197	B	3.84
13.	135	CG	ARG	14	A	<-->	4565	NE1	TRP	197	B	3.56
14.	136	CD	ARG	14	A	<-->	3937	O	GLY	123	B	3.89
15.	136	CD	ARG	14	A	<-->	3958	OG1	THR	125	B	3.26
16.	137	NE	ARG	14	A	<-->	3937	O	GLY	123	B	3.67

17.	137	NE	ARG	14	A	<-->	3958	OG1	THR	125	B	3.89
18.	139	CZ	ARG	14	A	<-->	3935	CA	GLY	123	B	3.86
19.	139	CZ	ARG	14	A	<-->	3936	C	GLY	123	B	3.82
20.	139	CZ	ARG	14	A	<-->	3937	O	GLY	123	B	3.19
21.	139	CZ	ARG	14	A	<-->	3958	OG1	THR	125	B	3.73
22.	140	NH1	ARG	14	A	<-->	3936	C	GLY	123	B	3.60
23.	140	NH1	ARG	14	A	<-->	3937	O	GLY	123	B	2.79
24.	140	NH1	ARG	14	A	<-->	3958	OG1	THR	125	B	2.80
25.	143	NH2	ARG	14	A	<-->	3935	CA	GLY	123	B	3.75
26.	143	NH2	ARG	14	A	<-->	3937	O	GLY	123	B	3.82
27.	154	O	PRO	15	A	<-->	4621	CE2	PHE	202	B	3.79
28.	176	O	ARG	17	A	<-->	3925	O	GLY	121	B	3.14
29.	176	O	ARG	17	A	<-->	3927	CA	PRO	122	B	3.50
30.	176	O	ARG	17	A	<-->	3931	C	PRO	122	B	3.52
31.	176	O	ARG	17	A	<-->	3932	O	PRO	122	B	3.59
32.	164	CG	ARG	17	A	<-->	4569	CZ2	TRP	197	B	3.75
33.	166	NE	ARG	17	A	<-->	4569	CZ2	TRP	197	B	3.65
34.	166	NE	ARG	17	A	<-->	4571	CH2	TRP	197	B	3.64
35.	168	CZ	ARG	17	A	<-->	4629	O	GLY	203	B	3.75
36.	172	NH2	ARG	17	A	<-->	4624	O	PHE	202	B	2.86
37.	172	NH2	ARG	17	A	<-->	4628	C	GLY	203	B	3.49
38.	172	NH2	ARG	17	A	<-->	4629	O	GLY	203	B	2.71
39.	172	NH2	ARG	17	A	<-->	4635	C	PRO	204	B	3.90
40.	172	NH2	ARG	17	A	<-->	4636	O	PRO	204	B	3.74
41.	179	CA	GLY	18	A	<-->	3932	O	PRO	122	B	3.34
42.	180	C	GLY	18	A	<-->	3932	O	PRO	122	B	3.56
43.	182	N	GLU	19	A	<-->	3932	O	PRO	122	B	2.86
44.	184	CA	GLU	19	A	<-->	3932	O	PRO	122	B	3.88
45.	207	CZ	ARG	21	A	<-->	3979	O	ALA	128	B	3.45
46.	208	NH1	ARG	21	A	<-->	3886	CD1	LEU	116	B	3.34
47.	208	NH1	ARG	21	A	<-->	3978	C	ALA	128	B	3.53
48.	208	NH1	ARG	21	A	<-->	3979	O	ALA	128	B	3.17
49.	208	NH1	ARG	21	A	<-->	3977	CB	ALA	128	B	3.32
50.	211	NH2	ARG	21	A	<-->	3979	O	ALA	128	B	3.78
51.	211	NH2	ARG	21	A	<-->	4009	CB	TYR	132	B	3.78
52.	211	NH2	ARG	21	A	<-->	4010	CG	TYR	132	B	3.84
53.	211	NH2	ARG	21	A	<-->	4012	CD2	TYR	132	B	3.78
54.	232	CG1	ILE	23	A	<-->	3998	CD2	TYR	131	B	3.83
55.	234	CD1	ILE	23	A	<-->	4011	CD1	TYR	132	B	3.71
56.	247	CG1	VAL	25	A	<-->	4024	O	GLY	133	B	3.61
57.	308	CB	GLN	32	A	<-->	4031	O	PRO	134	B	3.65
58.	309	CG	GLN	32	A	<-->	4031	O	PRO	134	B	3.83
59.	310	CD	GLN	32	A	<-->	4031	O	PRO	134	B	3.66
60.	311	OE1	GLN	32	A	<-->	4027	CB	PRO	134	B	3.71
61.	312	NE2	GLN	32	A	<-->	4026	CA	PRO	134	B	3.63
62.	340	CB	ARG	35	A	<-->	4016	OH	TYR	132	B	3.85
63.	342	CD	ARG	35	A	<-->	4016	OH	TYR	132	B	3.60
64.	345	CZ	ARG	35	A	<-->	3857	OD2	ASP	113	B	3.33
65.	346	NH1	ARG	35	A	<-->	3857	OD2	ASP	113	B	3.22
66.	349	NH2	ARG	35	A	<-->	3855	CG	ASP	113	B	3.65

67.	349	NH2	ARG	35	A	<-->	3857	OD2	ASP	113	B	2.59
68.	369	CB	ASP	37	A	<-->	4015	CZ	TYR	132	B	3.74
69.	369	CB	ASP	37	A	<-->	4016	OH	TYR	132	B	3.77
70.	372	OD2	ASP	37	A	<-->	4010	CG	TYR	132	B	3.27
71.	372	OD2	ASP	37	A	<-->	4011	CD1	TYR	132	B	3.25
72.	372	OD2	ASP	37	A	<-->	4012	CD2	TYR	132	B	3.43
73.	372	OD2	ASP	37	A	<-->	4013	CE1	TYR	132	B	3.39
74.	372	OD2	ASP	37	A	<-->	4014	CE2	TYR	132	B	3.57
75.	372	OD2	ASP	37	A	<-->	4015	CZ	TYR	132	B	3.54
76.	385	CA	ASP	39	A	<-->	3950	CZ3	TRP	124	B	3.82
77.	390	C	ASP	39	A	<-->	3950	CZ3	TRP	124	B	3.89
78.	391	O	ASP	39	A	<-->	3887	CD2	LEU	116	B	3.22
79.	391	O	ASP	39	A	<-->	3950	CZ3	TRP	124	B	3.22
80.	386	CB	ASP	39	A	<-->	3886	CD1	LEU	116	B	3.73
81.	386	CB	ASP	39	A	<-->	3887	CD2	LEU	116	B	3.79
82.	386	CB	ASP	39	A	<-->	3950	CZ3	TRP	124	B	3.85
83.	389	OD2	ASP	39	A	<-->	3886	CD1	LEU	116	B	3.70
84.	389	OD2	ASP	39	A	<-->	3937	O	GLY	123	B	3.74
85.	479	NH2	ARG	48	A	<-->	4027	CB	PRO	134	B	3.53
86.	911	O	ALA	90	A	<-->	4620	CE1	PHE	202	B	3.19
87.	911	O	ALA	90	A	<-->	4622	CZ	PHE	202	B	3.58
88.	923	C	SER	92	A	<-->	4578	OG	SER	198	B	3.37
89.	924	O	SER	92	A	<-->	4577	CB	SER	198	B	3.35
90.	924	O	SER	92	A	<-->	4578	OG	SER	198	B	2.71
91.	920	CB	SER	92	A	<-->	4572	C	TRP	197	B	3.82
92.	920	CB	SER	92	A	<-->	4573	O	TRP	197	B	3.39
93.	920	CB	SER	92	A	<-->	4563	CD1	TRP	197	B	3.54
94.	920	CB	SER	92	A	<-->	4578	OG	SER	198	B	3.66
95.	921	OG	SER	92	A	<-->	4563	CD1	TRP	197	B	3.17
96.	1212	C	ASP	119	A	<-->	4519	O	GLY	191	B	3.86
97.	1213	O	ASP	119	A	<-->	4519	O	GLY	191	B	3.14
98.	1213	O	ASP	119	A	<-->	4521	CA	PRO	192	B	3.49
99.	1213	O	ASP	119	A	<-->	4525	C	PRO	192	B	3.60
100.	1213	O	ASP	119	A	<-->	4527	N	GLY	193	B	2.78
101.	1213	O	ASP	119	A	<-->	4529	CA	GLY	193	B	3.73
102.	1216	CA	GLY	120	A	<-->	4518	C	GLY	191	B	3.81
103.	1216	CA	GLY	120	A	<-->	4519	O	GLY	191	B	3.58
104.	1216	CA	GLY	120	A	<-->	4520	N	PRO	192	B	3.88
105.	1216	CA	GLY	120	A	<-->	4521	CA	PRO	192	B	3.74
106.	1217	C	GLY	120	A	<-->	4521	CA	PRO	192	B	3.63
107.	1218	O	GLY	120	A	<-->	4521	CA	PRO	192	B	3.83
108.	1218	O	GLY	120	A	<-->	4522	CB	PRO	192	B	3.82
109.	1218	O	GLY	120	A	<-->	4523	CG	PRO	192	B	3.71
110.	1908	CE1	HIS	188	A	<-->	3703	CG	HIS	95	B	3.59
111.	1908	CE1	HIS	188	A	<-->	3704	ND1	HIS	95	B	3.81
112.	1908	CE1	HIS	188	A	<-->	3705	CD2	HIS	95	B	3.72
113.	1909	NE2	HIS	188	A	<-->	3702	CB	HIS	95	B	3.80
114.	1909	NE2	HIS	188	A	<-->	3703	CG	HIS	95	B	3.62
115.	1909	NE2	HIS	188	A	<-->	3705	CD2	HIS	95	B	3.59
116.	1925	CB	THR	190	A	<-->	3689	CG2	THR	93	B	3.73

117.	1926	OG1	THR	190	A	<-->	3689	CG2	THR	93	B	3.35
118.	1928	CG2	THR	190	A	<-->	3696	CD	PRO	94	B	3.69
119.	1946	CB	HIS	192	A	<-->	3664	OD1	ASN	91	B	3.21
120.	1947	CG	HIS	192	A	<-->	3664	OD1	ASN	91	B	3.48
121.	1948	ND1	HIS	192	A	<-->	3682	O	LYS	92	B	3.84
122.	1949	CD2	HIS	192	A	<-->	3661	CA	ASN	91	B	3.58
123.	1949	CD2	HIS	192	A	<-->	3668	C	ASN	91	B	3.87
124.	1949	CD2	HIS	192	A	<-->	3664	OD1	ASN	91	B	3.36
125.	1950	CE1	HIS	192	A	<-->	3628	CG1	VAL	88	B	3.81
126.	1950	CE1	HIS	192	A	<-->	3681	C	LYS	92	B	3.66
127.	1950	CE1	HIS	192	A	<-->	3682	O	LYS	92	B	3.10
128.	1951	NE2	HIS	192	A	<-->	3631	O	VAL	88	B	3.16
129.	1951	NE2	HIS	192	A	<-->	3628	CG1	VAL	88	B	3.68
130.	1951	NE2	HIS	192	A	<-->	3661	CA	ASN	91	B	3.89
131.	1951	NE2	HIS	192	A	<-->	3670	N	LYS	92	B	3.82
132.	1951	NE2	HIS	192	A	<-->	3682	O	LYS	92	B	3.64
133.	1959	C	ALA	193	A	<-->	3665	ND2	ASN	91	B	3.88
134.	1960	O	ALA	193	A	<-->	3665	ND2	ASN	91	B	3.17
135.	1963	CA	VAL	194	A	<-->	3665	ND2	ASN	91	B	3.83
136.	2035	CB	ARG	202	A	<-->	3687	OG1	THR	93	B	3.69
137.	2036	CG	ARG	202	A	<-->	3687	OG1	THR	93	B	3.84
138.	2037	CD	ARG	202	A	<-->	3687	OG1	THR	93	B	3.06
139.	2038	NE	ARG	202	A	<-->	3687	OG1	THR	93	B	3.81
140.	2040	CZ	ARG	202	A	<-->	3687	OG1	THR	93	B	3.70
141.	2041	NH1	ARG	202	A	<-->	3687	OG1	THR	93	B	2.74
142.	2061	CD1	TRP	204	A	<-->	3689	CG2	THR	93	B	3.56
143.	2063	NE1	TRP	204	A	<-->	3689	CG2	THR	93	B	3.67
144.	2063	NE1	TRP	204	A	<-->	3714	CB	ALA	96	B	3.73
145.	2065	CE2	TRP	204	A	<-->	3711	N	ALA	96	B	3.81
146.	2065	CE2	TRP	204	A	<-->	3713	CA	ALA	96	B	3.89
147.	2067	CZ2	TRP	204	A	<-->	3709	C	HIS	95	B	3.63
148.	2067	CZ2	TRP	204	A	<-->	3710	O	HIS	95	B	3.50
149.	2067	CZ2	TRP	204	A	<-->	3711	N	ALA	96	B	3.55
150.	2067	CZ2	TRP	204	A	<-->	3713	CA	ALA	96	B	3.32
151.	2067	CZ2	TRP	204	A	<-->	3714	CB	ALA	96	B	3.88
152.	2069	CH2	TRP	204	A	<-->	3710	O	HIS	95	B	3.49
153.	2083	CD1	LEU	206	A	<-->	3704	ND1	HIS	95	B	3.81
154.	2083	CD1	LEU	206	A	<-->	3706	CE1	HIS	95	B	3.87
155.	2084	CD2	LEU	206	A	<-->	3704	ND1	HIS	95	B	3.75
156.	2331	CB	VAL	231	A	<-->	4284	O	PRO	162	B	3.29
157.	2331	CB	VAL	231	A	<-->	4287	CA	GLY	163	B	3.68
158.	2332	CG1	VAL	231	A	<-->	4284	O	PRO	162	B	3.75
159.	2332	CG1	VAL	231	A	<-->	4287	CA	GLY	163	B	3.87
160.	2333	CG2	VAL	231	A	<-->	4284	O	PRO	162	B	3.50
161.	2336	N	GLU	232	A	<-->	4287	CA	GLY	163	B	3.72
162.	2340	CG	GLU	232	A	<-->	4288	C	GLY	163	B	3.76
163.	2340	CG	GLU	232	A	<-->	4290	N	PRO	164	B	3.63
164.	2354	O	THR	233	A	<-->	4056	CE1	TYR	138	B	3.84
165.	2354	O	THR	233	A	<-->	4058	CZ	TYR	138	B	3.76
166.	2354	O	THR	233	A	<-->	4059	OH	TYR	138	B	2.80

167.	2367	NH2	ARG	234	A	<-->	3716	O	ALA	96	B	3.39
168.	2376	CD	PRO	235	A	<-->	4056	CE1	TYR	138	B	3.54
169.	2384	O	ALA	236	A	<-->	3758	CB	MET	102	B	3.75
170.	2421	CE2	PHE	241	A	<-->	4059	OH	TYR	138	B	3.66
171.	2431	OE1	GLN	242	A	<-->	3735	CB	ALA	99	B	3.17
172.	2432	NE2	GLN	242	A	<-->	3761	CE	MET	102	B	3.55

Salt bridges

	<----- A T O M 1 ----->						<----- A T O M 2 ----->					
	Atom no.	Atom name	Res name	Res no.	Chain		Atom no.	Atom name	Res name	Res no.	Chain	Distance
1.	349	NH2	ARG	35	A	<-->	3857	OD2	ASP	113	B	2.59

Number of salt bridges: 1
Number of hydrogen bonds: 13
Number of non-bonded contacts: 172

Supplementary material SM 5:
Vaccine-MHC II PDBsum interacting molecules

Hydrogen bonds

	<----- A T O M 1 ----->						<----- A T O M 2 ----->					
	Atom no.	Atom name	Res name	Res no.	Chain		Atom no.	Atom name	Res name	Res no.	Chain	Distance
1.	8	OE2	GLU	1	A	<-->	4922	N	GLY	133	B	2.69
2.	833	OE2	GLU	86	A	<-->	4503	NZ	LYS	85	B	2.64
3.	860	O	VAL	89	A	<-->	4609	NE2	HIS	95	B	2.83
4.	1324	NH2	ARG	138	A	<-->	4728	OG	SER	110	B	3.20
5.	1335	OE1	GLU	139	A	<-->	4918	OH	TYR	132	B	2.56
6.	1347	O	ASP	140	A	<-->	4718	NZ	LYS	109	B	3.14
7.	1345	OD2	ASP	140	A	<-->	4728	OG	SER	110	B	2.65
8.	1649	OD1	ASP	169	A	<-->	4397	NH2	ARG	74	B	2.72
9.	2069	NH2	ARG	207	A	<-->	4918	OH	TYR	132	B	2.75
10.	2122	OE1	GLN	212	A	<-->	4718	NZ	LYS	109	B	2.97
11.	2176	NH2	ARG	217	A	<-->	4782	O	PHE	115	B	2.87
12.	2849	NZ	LYS	283	A	<-->	4533	O	VAL	88	B	3.01
13.	2871	NE2	GLN	285	A	<-->	4566	OD1	ASN	91	B	2.85
14.	2933	OD1	ASN	291	A	<-->	4567	ND2	ASN	91	B	3.30
15.	3270	OE1	GLN	327	A	<-->	4961	OH	TYR	138	B	2.76
16.	3401	OE2	GLU	340	A	<-->	4556	ND2	ASN	90	B	2.95
17.	3401	OE2	GLU	340	A	<-->	4579	NZ	LYS	92	B	2.66

Non-bonded contacts

<----- A T O M 1 -----> <----- A T O M 2 ----->

	Atom no.	Atom name	Res name	Res no.	Chain		Atom no.	Atom name	Res name	Res no.	Chain	Distance
1.	1	N	GLU	1	A	<-->	4903	CZ	TYR	131	B	3.88
2.	1	N	GLU	1	A	<-->	5407	CA	GLY	189	B	3.74
3.	10	O	GLU	1	A	<-->	5414	CD	PRO	190	B	3.89
4.	4	CB	GLU	1	A	<-->	4926	O	GLY	133	B	3.48
5.	5	CG	GLU	1	A	<-->	4907	O	TYR	131	B	3.87
6.	5	CG	GLU	1	A	<-->	4926	O	GLY	133	B	3.69
7.	6	CD	GLU	1	A	<-->	4922	N	GLY	133	B	3.61
8.	6	CD	GLU	1	A	<-->	4926	O	GLY	133	B	3.60
9.	8	OE2	GLU	1	A	<-->	4907	O	TYR	131	B	3.73
10.	8	OE2	GLU	1	A	<-->	4910	CA	TYR	132	B	3.26
11.	8	OE2	GLU	1	A	<-->	4920	C	TYR	132	B	3.43
12.	8	OE2	GLU	1	A	<-->	4922	N	GLY	133	B	2.69
13.	8	OE2	GLU	1	A	<-->	4924	CA	GLY	133	B	3.72
14.	8	OE2	GLU	1	A	<-->	4925	C	GLY	133	B	3.66
15.	8	OE2	GLU	1	A	<-->	4926	O	GLY	133	B	3.55
16.	20	O	GLU	2	A	<-->	4939	N	PRO	136	B	3.39
17.	20	O	GLU	2	A	<-->	4940	CA	PRO	136	B	3.62
18.	20	O	GLU	2	A	<-->	4941	CB	PRO	136	B	3.53
19.	20	O	GLU	2	A	<-->	4942	CG	PRO	136	B	3.78
20.	20	O	GLU	2	A	<-->	4943	CD	PRO	136	B	3.30
21.	36	CB	VAL	4	A	<-->	4928	CA	PRO	134	B	3.76
22.	38	CG2	VAL	4	A	<-->	4928	CA	PRO	134	B	3.58
23.	240	CA	ASP	25	A	<-->	4933	O	PRO	134	B	3.67
24.	245	C	ASP	25	A	<-->	4933	O	PRO	134	B	3.72
25.	246	O	ASP	25	A	<-->	4933	O	PRO	134	B	3.83
26.	241	CB	ASP	25	A	<-->	4936	CA	GLY	135	B	3.89
27.	242	CG	ASP	25	A	<-->	4936	CA	GLY	135	B	3.71
28.	242	CG	ASP	25	A	<-->	4943	CD	PRO	136	B	3.63
29.	243	OD1	ASP	25	A	<-->	4934	N	GLY	135	B	3.73
30.	243	OD1	ASP	25	A	<-->	4936	CA	GLY	135	B	3.56
31.	244	OD2	ASP	25	A	<-->	4943	CD	PRO	136	B	3.32
32.	815	CG	PRO	84	A	<-->	4390	CD	ARG	74	B	3.74
33.	815	CG	PRO	84	A	<-->	4391	NE	ARG	74	B	3.68
34.	816	CD	PRO	84	A	<-->	4390	CD	ARG	74	B	3.40
35.	816	CD	PRO	84	A	<-->	4391	NE	ARG	74	B	3.69
36.	831	CD	GLU	86	A	<-->	4503	NZ	LYS	85	B	3.37
37.	832	OE1	GLU	86	A	<-->	4661	CG	MET	102	B	3.56
38.	832	OE1	GLU	86	A	<-->	4662	SD	MET	102	B	3.45
39.	833	OE2	GLU	86	A	<-->	4502	CE	LYS	85	B	3.58
40.	833	OE2	GLU	86	A	<-->	4503	NZ	LYS	85	B	2.64
41.	860	O	VAL	89	A	<-->	4607	CD2	HIS	95	B	3.58
42.	860	O	VAL	89	A	<-->	4608	CE1	HIS	95	B	3.83
43.	860	O	VAL	89	A	<-->	4609	NE2	HIS	95	B	2.83
44.	866	CD1	LEU	90	A	<-->	4607	CD2	HIS	95	B	3.81

45. 1018 CE2 PHE 106 A <--> 4663 CE MET 102 B 3.68
46. 1019 CZ PHE 106 A <--> 4663 CE MET 102 B 3.77
47. 1037 OD2 ASP 108 A <--> 4662 SD MET 102 B 3.58
48. 1037 OD2 ASP 108 A <--> 4663 CE MET 102 B 3.26
49. 1046 CE LYS 109 A <--> 4662 SD MET 102 B 3.38
50. 1047 NZ LYS 109 A <--> 4662 SD MET 102 B 3.66
51. 1324 NH2 ARG 138 A <--> 4695 CA ALA 106 B 3.59
52. 1324 NH2 ARG 138 A <--> 4697 C ALA 106 B 3.81
53. 1324 NH2 ARG 138 A <--> 4698 O ALA 106 B 3.49
54. 1324 NH2 ARG 138 A <--> 4696 CB ALA 106 B 3.38
55. 1324 NH2 ARG 138 A <--> 4728 OG SER 110 B 3.20
56. 1338 O GLU 139 A <--> 4768 CD2 LEU 114 B 3.33
57. 1332 CB GLU 139 A <--> 4727 CB SER 110 B 3.65
58. 1332 CB GLU 139 A <--> 4728 OG SER 110 B 3.66
59. 1332 CB GLU 139 A <--> 4918 OH TYR 132 B 3.89
60. 1333 CG GLU 139 A <--> 4918 OH TYR 132 B 3.90
61. 1334 CD GLU 139 A <--> 4918 OH TYR 132 B 3.52
62. 1334 CD GLU 139 A <--> 4930 CG PRO 134 B 3.74
63. 1335 OE1 GLU 139 A <--> 4917 CZ TYR 132 B 3.36
64. 1335 OE1 GLU 139 A <--> 4918 OH TYR 132 B 2.56
65. 1339 N ASP 140 A <--> 4728 OG SER 110 B 3.89
66. 1347 O ASP 140 A <--> 4717 CE LYS 109 B 3.89
67. 1347 O ASP 140 A <--> 4718 NZ LYS 109 B 3.14
68. 1347 O ASP 140 A <--> 4759 OD2 ASP 113 B 3.88
69. 1342 CB ASP 140 A <--> 4722 C LYS 109 B 3.50
70. 1342 CB ASP 140 A <--> 4723 O LYS 109 B 3.46
71. 1342 CB ASP 140 A <--> 4714 CB LYS 109 B 3.82
72. 1342 CB ASP 140 A <--> 4717 CE LYS 109 B 3.51
73. 1342 CB ASP 140 A <--> 4718 NZ LYS 109 B 3.71
74. 1342 CB ASP 140 A <--> 4724 N SER 110 B 3.59
75. 1342 CB ASP 140 A <--> 4726 CA SER 110 B 3.67
76. 1343 CG ASP 140 A <--> 4724 N SER 110 B 3.86
77. 1343 CG ASP 140 A <--> 4728 OG SER 110 B 3.53
78. 1345 OD2 ASP 140 A <--> 4698 O ALA 106 B 3.35
79. 1345 OD2 ASP 140 A <--> 4722 C LYS 109 B 3.75
80. 1345 OD2 ASP 140 A <--> 4714 CB LYS 109 B 3.61
81. 1345 OD2 ASP 140 A <--> 4724 N SER 110 B 3.20
82. 1345 OD2 ASP 140 A <--> 4726 CA SER 110 B 3.51
83. 1345 OD2 ASP 140 A <--> 4727 CB SER 110 B 3.61
84. 1345 OD2 ASP 140 A <--> 4728 OG SER 110 B 2.65
85. 1365 CD1 LEU 142 A <--> 4717 CE LYS 109 B 3.69
86. 1648 CG ASP 169 A <--> 4397 NH2 ARG 74 B 3.45
87. 1649 OD1 ASP 169 A <--> 4391 NE ARG 74 B 3.88
88. 1649 OD1 ASP 169 A <--> 4393 CZ ARG 74 B 3.72
89. 1649 OD1 ASP 169 A <--> 4397 NH2 ARG 74 B 2.72
90. 1650 OD2 ASP 169 A <--> 4397 NH2 ARG 74 B 3.46
91. 1676 CD2 LEU 172 A <--> 4471 CE LYS 82 B 3.85
92. 1676 CD2 LEU 172 A <--> 4472 NZ LYS 82 B 3.80
93. 1945 CD1 PHE 196 A <--> 4900 CD2 TYR 131 B 3.76
94. 1945 CD1 PHE 196 A <--> 4902 CE2 TYR 131 B 3.51

95.	1947	CE1	PHE	196	A	<-->	4900	CD2	TYR	131	B	3.78
96.	2065	CZ	ARG	207	A	<-->	4918	OH	TYR	132	B	3.88
97.	2066	NH1	ARG	207	A	<-->	4768	CD2	LEU	114	B	3.37
98.	2069	NH2	ARG	207	A	<-->	4918	OH	TYR	132	B	2.75
99.	2122	OE1	GLN	212	A	<-->	4718	NZ	LYS	109	B	2.97
100.	2170	NE	ARG	217	A	<-->	4853	CH2	TRP	124	B	3.70
101.	2172	CZ	ARG	217	A	<-->	4782	O	PHE	115	B	3.70
102.	2172	CZ	ARG	217	A	<-->	4789	CD2	LEU	116	B	3.80
103.	2173	NH1	ARG	217	A	<-->	4782	O	PHE	115	B	3.84
104.	2176	NH2	ARG	217	A	<-->	4782	O	PHE	115	B	2.87
105.	2176	NH2	ARG	217	A	<-->	4789	CD2	LEU	116	B	3.84
106.	2223	CG1	VAL	222	A	<-->	4834	O	PRO	122	B	3.76
107.	2223	CG1	VAL	222	A	<-->	4830	CB	PRO	122	B	3.70
108.	2224	CG2	VAL	222	A	<-->	4834	O	PRO	122	B	3.50
109.	2224	CG2	VAL	222	A	<-->	4837	CA	GLY	123	B	3.68
110.	2259	CB	ARG	226	A	<-->	4851	CZ2	TRP	124	B	3.85
111.	2265	NH1	ARG	226	A	<-->	4831	CG	PRO	122	B	3.61
112.	2268	NH2	ARG	226	A	<-->	4847	NE1	TRP	124	B	3.85
113.	2847	CD	LYS	283	A	<-->	4566	OD1	ASN	91	B	3.41
114.	2848	CE	LYS	283	A	<-->	4533	O	VAL	88	B	3.84
115.	2849	NZ	LYS	283	A	<-->	4533	O	VAL	88	B	3.01
116.	2868	CG	GLN	285	A	<-->	4566	OD1	ASN	91	B	3.81
117.	2869	CD	GLN	285	A	<-->	4566	OD1	ASN	91	B	3.79
118.	2871	NE2	GLN	285	A	<-->	4566	OD1	ASN	91	B	2.85
119.	2930	CA	ASN	291	A	<-->	4567	ND2	ASN	91	B	3.61
120.	2937	C	ASN	291	A	<-->	4567	ND2	ASN	91	B	3.58
121.	2933	OD1	ASN	291	A	<-->	4567	ND2	ASN	91	B	3.30
122.	2939	N	LEU	292	A	<-->	4567	ND2	ASN	91	B	3.30
123.	2942	CB	LEU	292	A	<-->	4571	O	ASN	91	B	3.40
124.	2942	CB	LEU	292	A	<-->	4567	ND2	ASN	91	B	3.60
125.	2943	CG	LEU	292	A	<-->	4571	O	ASN	91	B	3.84
126.	2945	CD2	LEU	292	A	<-->	4571	O	ASN	91	B	3.49
127.	3055	O	ILE	305	A	<-->	5195	CG	PRO	164	B	3.86
128.	3055	O	ILE	305	A	<-->	5196	CD	PRO	164	B	3.25
129.	3189	CD	LYS	317	A	<-->	5754	OD1	ASN	229	B	3.70
130.	3190	CE	LYS	317	A	<-->	5754	OD1	ASN	229	B	3.84
131.	3221	CG2	VAL	321	A	<-->	4578	CE	LYS	92	B	3.61
132.	3227	CB	SER	322	A	<-->	5182	CB	PRO	162	B	3.55
133.	3238	CG2	THR	323	A	<-->	4615	CA	ALA	96	B	3.65
134.	3238	CG2	THR	323	A	<-->	4617	C	ALA	96	B	3.85
135.	3238	CG2	THR	323	A	<-->	4618	O	ALA	96	B	3.27
136.	3238	CG2	THR	323	A	<-->	4637	CB	ALA	99	B	3.59
137.	3243	CA	GLY	324	A	<-->	5186	O	PRO	162	B	3.51
138.	3244	C	GLY	324	A	<-->	5186	O	PRO	162	B	3.87
139.	3269	CD	GLN	327	A	<-->	4961	OH	TYR	138	B	3.65
140.	3270	OE1	GLN	327	A	<-->	4960	CZ	TYR	138	B	3.68
141.	3270	OE1	GLN	327	A	<-->	4961	OH	TYR	138	B	2.76
142.	3381	SD	MET	338	A	<-->	4589	OG1	THR	93	B	3.52
143.	3382	CE	MET	338	A	<-->	4589	OG1	THR	93	B	3.29
144.	3399	CD	GLU	340	A	<-->	4556	ND2	ASN	90	B	3.72

```
145.   3399  CD  GLU  340    A   <--> 4579  NZ  LYS   92    B      3.11
146.   3400  OE1 GLU  340    A   <--> 4556  ND2 ASN   90    B      3.75
147.   3400  OE1 GLU  340    A   <--> 4579  NZ  LYS   92    B      3.14
148.   3401  OE2 GLU  340    A   <--> 4560  O   ASN   90    B      3.09
149.   3401  OE2 GLU  340    A   <--> 4556  ND2 ASN   90    B      2.95
150.   3401  OE2 GLU  340    A   <--> 4576  CG  LYS   92    B      3.63
151.   3401  OE2 GLU  340    A   <--> 4578  CE  LYS   92    B      3.51
152.   3401  OE2 GLU  340    A   <--> 4579  NZ  LYS   92    B      2.66
```

Salt bridges

```
      <----- A T O M  1 ----->       <----- A T O M  2 ----->

      Atom Atom Res  Res              Atom Atom Res  Res
       no. name name no.  Chain        no. name name no.  Chain  Distance
  1.    833  OE2 GLU   86    A   <--> 4503  NZ  LYS   85    B      2.64
  2.   1649  OD1 ASP  169    A   <--> 4397  NH2 ARG   74    B      2.72
  3.   3401  OE2 GLU  340    A   <--> 4579  NZ  LYS   92    B      2.66
```

Number of salt bridges: 3
Number of hydrogen bonds: 17
Number of non-bonded contacts: 152

Supplementary material SM 6:
cDNA sequence of the vaccine construct:

ATGACCCCGCAGAACATCACCGACCTGTGCGCTGAATACCACAACACCCAGATC
CACACCCTGAACGACAAAATCTTCTCTTACACCGAATCTCTGGCTGGTAAACGTG
AAATGGCTATCATCACCTTCAAAAACGGTGCTACCTTCCAGGTTGAAGTTCCGGG
TTCTCAGCACATCGACTCTCAGAAAAAAGCTATCGAACGTATGAAAGACACCCT
GCGTATCGCTTACCTGACCGAAGCTAAAGTTGAAAAACTGTGCGTTTGGAACAA
CAAAACCCCGCACGCTATCGCTGCTATCTCTATGGCTAACGAAGCTGCTGCTAAA
TCTACCCAGGACCTGTTCCTGCCGTTCGGTCCGGGTCCGGGTTGGACCGCTGGTG
CTGCTGCTTACTACGGTCCGGGTCCGGGTTACCTGCAGCCGCGTACCTTCCTGCT
GGGTCCGGGTCCGGGTTACCAGCCGTACCGTGTTGTTGTTCTGGGTCCGGGTCCG
GGTTTCACCATCTCTGTTACCACCGAAATCGGTCCGGGTCCGGGTCAGATCATCA
CCACCGACAACACCTTCGGTCCGGGTCCGGGTCACTCTGCTTGGTCTCACCCGCA
GTTCGGTCCGGGTCCGGGTGGTATCAACATCACCCGTTTCCAGACCCTGCTGGCT
CTGCACCGTGGTCCGGGTCCGGGTATCAACATCACCCGTTTCCAGACCCTGCTGG
CTCTGCACCGTTCTGGTCCGGGTCCGGGTCAGCCGTACCGTGTTGTTGTTCTGTCT
TTCGAACTGCTGCACGCTGGTCCGGGTCCGGGTCCGTACCGTGTTGTTGTTCTGTC
TTTCGAACTGCTGCACGCTCCGGGTCCGGGTCCGGGTGGTTGGACCTTCGGTGCT
GGTGCTGCTCTGCAGATCCCGTTCGCTGGTCCGGGTCCGGGTTGGACCTTCGGTG
CTGGTGCTGCTCTGCAGATCCCGTTCGCTATGGGTCCGGGTCCGGGTATCCGTGC
TGCTGAAATCCGTGCTTCTGCTAACCTGGCTGCTACCGGTCCGGGTCCGGGTGCT
GCTGAAATCCGTGCTTCTGCTAACCTGGCTGCTACCAAAATGGGTCCGGGTCCGG

GTACCCGTTTCGCTTCTGTTTACGCTTGGAACCGTAAACGTATCTCTGGTCCGGGT
CCGGGTGGTATCAACATCACCCGTTTCCAGACCCTGCTGGCTCTGCACCGTGGTC
CGGGTCCGGGTATCAACATCACCCGTTTCCAGACCCTGCTGGCTCTGCACCGTTC
T
